# Assembly strategy for thieno[3,2-*b*]thiophenes via a disulfide intermediate derived from 3-nitrothiophene-2,5-dicarboxylate

**DOI:** 10.3762/bjoc.21.191

**Published:** 2025-11-11

**Authors:** Roman A Irgashev

**Affiliations:** 1 Postovsky Institute of Organic Synthesis, Ural Branch of the Russian Academy of Sciences, Ekaterinburg, 620137, Russia

**Keywords:** aromatic nucleophilic substitution, disulfide derivative, 3-nitrothiophene, organic disulfides, thieno[3,2-*b*]thiophene, thiophene ring closure

## Abstract

A versatile synthetic route to thieno[3,2-*b*]thiophenes was elaborated from dimethyl 3-nitrothiophene-2,5-dicarboxylate. Nucleophilic substitution of the nitro group with sulfur nucleophiles, including thioacetate or disulfide anions as well as thioacetamide, yielded bis(thiophen-3-yl)disulfide and sulfide derivatives. The disulfide served as a suitable precursor for the preparation of 3-alkylthio-substituted thiophene-2,5-dicarboxylates by its one-pot reduction–alkylation using NaBH_4_ in DMF followed by an alkylating agent. Base-promoted cyclization of electron-deficient 3-alkylthio derivatives furnished 2-aryl-, 2-aroyl-, and 2-cyano-substituted thieno[3,2-*b*]thiophenes, bearing a 3-hydroxy group. This protocol broadens access to functionalized thieno[3,2-*b*]thiophenes with potential applications in pharmaceutical and materials chemistry.

## Introduction

Thieno[3,2-*b*]thiophene (TT) derivatives are a valuable class of fused heteroaromatic compounds characterized by rigid planar π-conjugated backbones that provide narrow band gaps, high charge carrier mobility, and intermolecular π–π stacking interactions, making them particularly attractive for the design of organic materials. Indeed, the use of TT frameworks for semiconductors has led to significant progress in organic field-effect transistors (OFETs), organic photovoltaics, organic light-emitting diodes, and chemical sensors [1–3]. For instance, TT-containing copolymers have shown high hole mobility, strong absorption in the visible region, and improved morphological stability [4–6]. Recent reports have shown that the development of alkyl-supported TT-based semiconductors has led to high-performance, air-stable OFETs [7], while integration of TT frameworks into donor–acceptor architectures has enabled the development of materials for various types of organic solar cells [8–13]. TT-extended phthalocyanines and related dyes have also been applied in gas-sensing devices, benefiting from their π-stacked alignment and electron-rich character [14].

On the other hand, the biological potential of TT molecules has attracted increasing interest as numerous TT-based compounds were proposed as promising candidates in medicinal chemistry due to their diverse pharmacological profiles including antitumor, antiviral, antimicrobial, enzymatic and phototherapeutic activities [15]. Among them, TT-2-carboxanilides exhibit notable DNA intercalation and antiproliferative effects [16–17], while TT–BODIPY derivatives were shown to efficiently generate singlet oxygen and demonstrate light-induced cytotoxicity, highlighting their promise as photodynamic therapeutic agents [18]. Near-infrared TT–DPP-based dyes have also been developed for photothermal and photodynamic therapy, where their π-conjugated frameworks contribute to both imaging and therapeutic functions [19]. Some other TT-based compounds have been reported to inhibit specific carbonic anhydrase isoforms [20], protein tyrosine phosphatase 1B [21], as well as to act as agonists of the GPR35 receptor [22], further underscoring the value of the TT scaffold in enzyme- and receptor-targeted drug discovery.

**Scheme 1:**
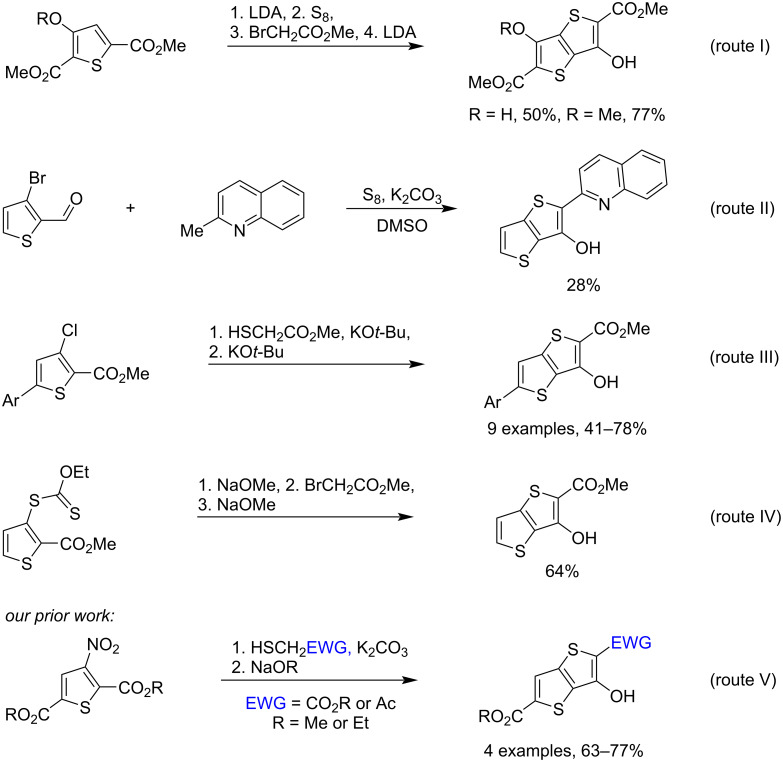
The synthetic routes to 3-hydroxy-substituted TT derivatives.

Given the established practical significance of thieno[3,2-*b*]thiophene compounds, the construction of molecules featuring the TT core remains a key objective in organic synthesis. A widely adopted strategy for building the TT scaffold involves the annulation of a second thiophene ring onto a suitably functionalized thiophene precursor. Most commonly, this approach starts from halogenated thiophenes such as 3-bromo- or 3-chlorothiophenes, which undergo subsequent cyclization to furnish the TT framework [2,23–24]. Despite the wide structural diversity of TT derivatives, 3-hydroxy-substituted analogues remain rare, with only a few synthetic strategies described for their preparation, as illustrated in Scheme 1. One such route involves sulfur insertion into a thiophen-3-yl lithium derivative, followed by electrophilic trapping of the thiolate and base-induced cyclization to afford the 3-hydroxy-TT [25]. Route II is represented by the single example of a one-pot reaction of 2-methylquinoline and 3-bromothiophene-2-carbaldehyde in the presence of elemental S and K_2_CO_3_ in DMSO to give the desired product [26]. Route III, previously elaborated in our group, utilizes the nucleophilic substitution of the Cl atom in 3-chlorothiophene-2-carboxylates by methyl thioglycolate in the presence of KO*t*-Bu, followed by KO*t*-Bu-mediated cyclization to the 3-hydroxy-TTs [27]. In route IV, cleavage of the ethyl xanthate group in the starting substrate by NaOMe generates a thiolate intermediate, which undergoes S-alkylation and subsequent NaOMe-promoted cyclization to afford the 3-hydroxy-TT [28]. In our recent works, it was presented an effective strategy for synthesizing TTs from the activated 3-nitrosubstituted thiophenes [29–30]. This strategy provides an effective access to the 3-hydroxy-TTs via the nucleophilic substitution of the nitro group in 3-nitrothiophene precursors using S-nucleophiles, such as alkyl thioglycolates or mercaptoacetone (Scheme 1, route V).

**Scheme 2:**
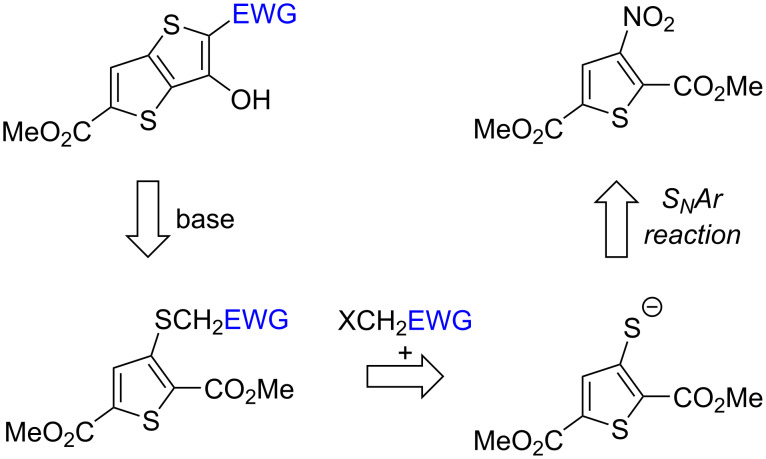
The present retrosynthetic plan for constructing TT molecules.

Herein, we wish to report a modified strategy based on route IV involving the stepwise construction of the TT framework. This approach is accomplished via the nucleophilic substitution of the nitro group with a S-nucleophile to obtain the thiophene-3-thiolate or its equivalent, followed by further S-alkylation and base-promoted cyclization to form the 3-hydroxy-TT molecules (Scheme 2).

## Results and Discussion

**Scheme 3:**
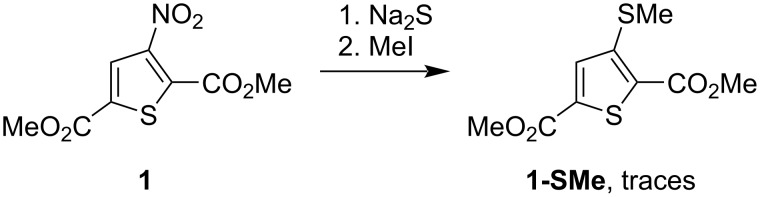
An attempt to nucleophilically substitute the NO_2_ group in ester **1**.

We began our study by investigating the reaction of dimethyl 3-nitrothiophene-2,5-dicarboxylate (**1**) with Na_2_S, inspired by Beck’s reported synthesis of 2-substituted-3-aminobenzo[*b*]thiophenes via the nucleophilic substitution of the nitro group in 2-nitrobenzonitriles using Na_2_S in a DMF/water medium, followed by alkylation of the resulting 2-cyanophenylthiolates and subsequent cyclization [31]. However, in our case, the reaction of ester **1** with Na_2_S in either acetone or a DMF/water mixture predominantly led to degradation of the starting material. Subsequent treatment with iodomethane gave a complex mixture of compounds, in which only trace amounts of the expected derivative **1-SMe** were detected by GC–MS analysis (Scheme 3). In this regard, the reaction of 3-nitrothiophene **1** with Na_2_S turned out to be an unsuitable route for nucleophilic substitution of its nitro group.

**Scheme 4:**
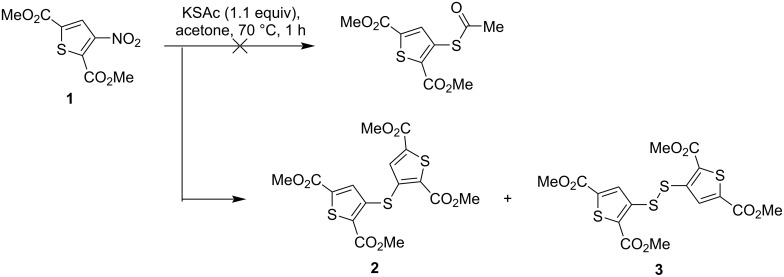
The reaction of ester **1** with potassium thioacetate.

Next, we explored an alternative route using potassium thioacetate (KSAc) as a softer nucleophile. Unexpectedly, the reaction of ester **1** with KSAc in acetone gave a mixture of products other than the desired 3-AcS-substituted thiophene, promoting further study of these structures and reaction mechanism. ^1^H NMR spectroscopy of the obtained mixture provided the first key information about its nature, namely the presence of two major products **2** and **3**, both of which showed three characteristic singlets with signal integration 1:3:3. For compound **2**, these signals appeared at 7.40, 3.91, and 3.89 ppm, whereas for compound **3**, the signals were observed at 7.83, 3.97, and 3.87 ppm. These data strongly suggest the absence of a proton at the C-3 position of the thiophene ring and the presence of two methoxycarbonyl groups. Further isolation of compounds **2** and **3** in analytically pure form allowed their structure to be established thanks to elemental analysis and HRMS. It was found that compounds **2** and **3** are dimeric derivatives of the starting thiophene, linked by sulfur bridges located at the C-3 position instead of the nitro group. Compound **2** was identified as a bis(thiophen-3-yl)sulfide derivative and compound **3** as a bis(thiophen-3-yl)disulfide derivative, both with methoxycarbonyl groups at the C-2 and C-5 positions of each thiophene ring (Scheme 4).

**Scheme 5:**
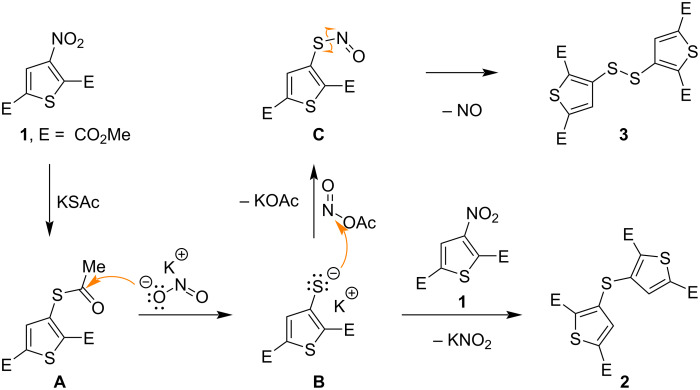
A probable mechanism for the formation of compounds **2** and **3**.

The formation of these unexpected products, sulfide **2** and disulfide **3**, necessitates consideration of a reaction mechanism beyond straightforward nucleophilic substitution of the nitro group in ester **1**. Although the initial reaction probably involves a nucleophilic attack by the S-nucleophile on the activated thiophene ring, resulting in displacement of the nitro group, the subsequent fate of the intermediate appears to be critical. Based on our observations and literature data, a probable reaction mechanism was proposed. The initial nucleophilic substitution of the nitro group in ester **1** by the AcS^−^ anion yields the *S*-acetyl derivative (intermediate **A**) and NO_2_^−^ anion. The key step leading to the observed products is the reaction of the *S*-acetyl intermediate **A** with the generated NO_2_^−^ anion, resulting in the formation of a thiophene-3-thiolate species (intermediate **B**) and acetyl nitrite. Thiolate **B** then acts as a nucleophile toward starting ester **1**, resulting in the formation of sulfide **2**. Simultaneously, thiolate **B** reacts with acetyl nitrite to form the *S*-nitrosothiol intermediate **C** and the acetate anion. *S*-Nitrosothiol **C** subsequently undergoes decomposition, losing nitrogen oxide to give disulfide **3** (Scheme 5). This proposed mechanism, in particular the conversion of the *S*-acetyl compound to the thiolate and then to the disulfide with the participation of nitrite, is consistent with the previously reported synthesis of bis(per-*O*-acetylated glycosyl)disulfides from *S*-acetyl glycosides and KNO_2_, providing a literature precedent for similar transformations [32].

In addition to potassium thioacetate, the reactivity of ester **1** was investigated with other related S-nucleophiles, such as potassium ethyl xanthate (KSC(S)OEt), sodium diethyldithiocarbamate (NaSC(S)NEt_2_) as well as thioacetamide in the presence of K_2_CO_3_ and Na_2_S_2_ (Table 1, entries 2–5). In each case, the reaction resulted in the formation of a mixture of sulfide **2** and disulfide **3**, although the ratio of products varied considerably depending on the nature of the used S-nucleophile. Thus, the reaction with KSAc gave a **2**/**3** ratio of 1:5.82 (Table 1, entry 1), which greatly favored disulfide formation. The use of KSC(S)OEt and NaSC(S)NEt_2_ gave a **2**/**3** ratio of 1:0.92 and 1:0.8, respectively, indicating almost equivalent amounts of both products (Table 1, entries 2 and 3). The reaction with thioacetamide promoted the formation of sulfide **2** in a **2**/**3** ratio of 1:0.28 (Table 1, entry 4).

**Table 1:** The reaction of ester **1** with S-nucleophiles to form sulfide **2** and disulfide **3**.

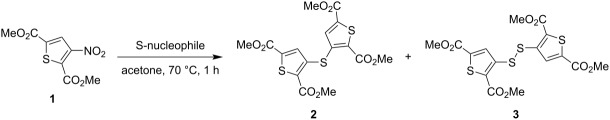			

entry	S-nucleophile	**2**/**3** ratio^a^	yield

1	KSAc (1.1 equiv)	1:5.82	**3**, 75%^b^
2	KSC(S)OEt (1.1 equiv)	1:0.92	**2** + **3**, 33%/**2**, 15%^b^
3	NaSC(S)NEt_2_ (1.1 equiv)	1:0.80	**2** + **3**, 27%
4	MeC(S)NH_2_ (1.2 equiv)/K_2_CO_3_	1:0.28	**2**, 51%^b^
5^c^	Na_2_S_2_ (0.527 equiv)	1:19.91	**3**, 71%^b^

^a^According to ^1^H NMR spectroscopy. ^b^The yield of product isolated in analytically pure form. ^c^A mixture of DMF/water/acetone was used in this experiment.

The most pronounced preference for disulfide formation was observed in the reaction with Na_2_S_2_, where the **2**/**3** ratio reached 1:19.91, indicating the almost exclusive formation of disulfide **3** (Table 1, entry 5). This result is consistent with a mechanism in which the nucleophilic substitution of the nitro group in ester **1** occurs via attack by the disulfide anion, wherein each sulfur atom reacts with one molecule **1**, resulting in the formation of product **3**. The minor presence of sulfide **2** in the product mixture can be attributed to partial decomposition of an intermediate aryl disulfide anion, which can fragment under the reaction conditions to release thiolate species capable of reacting separately with ester **1** to form compound **2**.

Disulfide **3** was found to be an accessible and stable precursor of dimethyl 3-mercaptothiophene-2,5-dicarboxylate, a molecule that is suitable for S-alkylation. In this regard, reductive cleavage of the S–S bond in disulfide **3** followed by in situ alkylation was investigated for the one-pot synthesis of 3-alkylthio-substituted thiophene-2,5-dicarboxylates. We first focused our efforts on optimizing the conditions of this two-step process to determine suitable reducing agents and solvents (Table 2). Thus, 4-(chloromethyl)benzonitrile was used as a model alkylating agent, NaBH_4_ and Na_2_S_2_O_4_ were used as mild and accessible reducing agents, and K_2_CO_3_ was originally considered as a base for the alkylation step. In our first experiment, NaBH_4_ in methanol was used to reduce disulfide **3** (Table 2, entry 1). However, methanol proved to be an ineffective solvent due to the rapid decomposition of NaBH_4_ and poor solubility of substrate **3**. Most of the starting material was recovered unchanged after complete decomposing NaBH_4_. In contrast, when ethanol (Table 2, entry 2) or isopropanol (Table 2, entry 3) was used as solvent, the reduction of disulfide **3** with NaBH_4_ proceeded more efficiently at reflux for 2 h. The reaction mixture was then treated with K_2_CO_3_ and the alkylating agent at room temperature. The product was identified as the desired 3-benzylthio-substituted thiophene-2,5-dicarboxylate, but it was obtained as a mixture of ester forms, namely dimethyl and diethyl esters in ethanol, and dimethyl and diisopropyl esters in isopropanol. These results indicate that the reaction with alcohol solvents resulted in partial transesterification of the methoxycarbonyl groups, while reductive cleavage of the S–S bond and the subsequent S-alkylation reaction were successful. To suppress the side reaction and improve reduction efficiency, we next employed DMF as a polar aprotic solvent. In Table 2, entry 4, reduction of disulfide **3** in DMF at 75 °C with NaBH_4_ was complete within 15 min. The excess reductant was quenched with methanol, and the reaction mixture was then treated with K_2_CO_3_ and the alkylating agent for 20 min, which allowed to obtain the desired product in 45% yield. A partial saponification of the ester groups was observed under these conditions, which contributed to the reduced yield. To address this, in a subsequent experiment (Table 2, entry 5), the alkylation step was performed without K_2_CO_3_, while DMF was retained as a solvent. The reaction proceeded cleanly, and product **4a** was isolated in 88% yield. This result confirms that under these conditions, base-promoted side reactions, probably involving ester hydrolysis or alkylating agent decomposition, can be avoided by omitting the external base. In addition, Na_2_S_2_O_4_ was investigated as a reductant in a DMF/water 9:1 (v/v) mixture at room temperature (Table 2, entry 6). In the presence of both K_2_CO_3_ and the alkylating agent, the reaction proceeded smoothly over 24 h to afford the desired product in 60% yield. In contrast, when the reaction was performed without K_2_CO_3_ (Table 2, entry 7), no product was obtained, indicating that a basic medium is essential not only for alkylation but also to enable effective S–S bond reduction by Na_2_S_2_O_4_ under these conditions. These experiments show that the optimal conditions for the one-pot synthesis of 3-(alkylthio)thiophene-2,5-dicarboxylates involve NaBH_4_-mediated reduction of substrate **3** in DMF, and next alkylation in the absence of added base (Table 2, entry 5).

**Table 2:** Optimization of the reaction conditions for the reduction–alkylation of disulfide **3**.

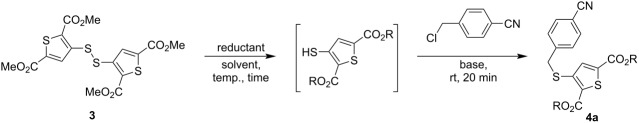						

entry^a^	reductant	solvent	temp.	time	base	yield

1	NaBH_4_	MeOH	rt	30 min	–	nr
2	NaBH_4_	EtOH	reflux	2 h	K_2_CO_3_	R = Me / R = Et^b^
3	NaBH_4_	iPrOH	reflux	2 h	K_2_CO_3_	R = Me / R = iPr^b^
4^c^	NaBH_4_	DMF/MeOH	75 °C	15 min	K_2_CO_3_	**4a**, R = Me, 45%^d^
5^c^	NaBH_4_	DMF/MeOH	75 °C	15 min	–	**4a**, R = Me, 88%^d^
6	Na_2_S_2_O_4_	DMF/H_2_O	rt	24 h	K_2_CO_3_	**4a**, R = Me, 60%^d^
7	Na_2_S_2_O_4_	DMF/H_2_O	rt	24 h	–	nr

^a^Sulfide **3** (0.4 mmol), NaBH_4_ (2.5 equiv) or Na_2_S_2_O_4_ (3 equiv) and 4-(chloromethyl)benzonitrile (2.2 equiv) were used in these experiments. ^b^A mixture of 3-RS-substituted dialkyl thiophene-2,5-dicarboxylates was obtained. ^c^The oil bath temperature is indicated. ^d^The yield of product isolated in analytically pure form.

A number of benzyl halides and other electrophiles were used under optimal reaction conditions to obtain various 3-(alkylthio)thiophene-2,5-dicarboxylates. Among them, compounds **4b**–**g**, bearing benzylthio substituents, were prepared using different benzyl-type alkylating agents. It was found that the reaction tolerated both electron-donating and electron-withdrawing groups on the aromatic ring, and the yields for this series ranged from 76% to 92%. Piperonyl mesylate was successfully used in the synthesis of compound **4g** (92% yield), while thiophene-2-ylmethyl mesylate was used for the preparation of compound **4h** (77% yield). For product **4b**, methyl 4-(bromomethyl)benzoate was employed as the alkylating agent. In all other cases, benzyl chlorides were used for the alkylation.

**Scheme 6:**
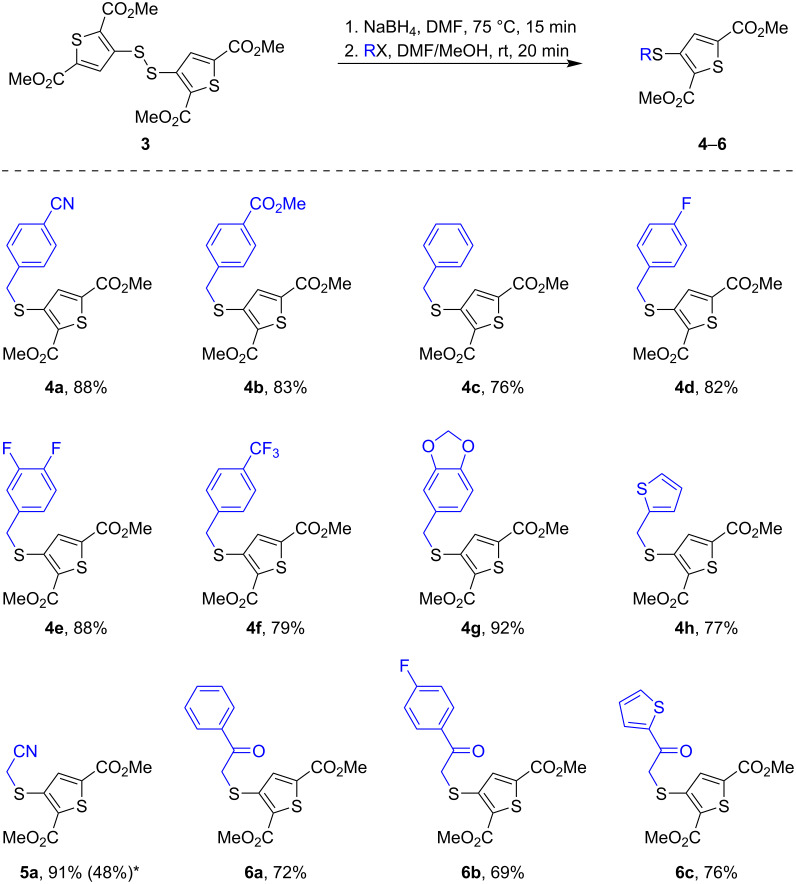
The synthesis of 3-(alkylthio)thiophene-2,5-dicarboxylates **4**–**6**, yields, and scope of products. *From BrCH_2_CN (from ClCH_2_CN).

The alkylation with chloroacetonitrile afforded 3-(cyanomethyl)thio derivative **5a** in 48% yield, shown in parenthesis in Scheme 6, while bromoacetonitrile significantly improved the result, allowing product **5a** to be obtained in 91% yield. Furthermore, we extended our approach to phenacyl-type alkylators to access 3-(phenacyl)thio compounds **6a** and **6b**, as well as their 3-[(thiophen-2-yl)carbonylmethyl]thio analog **6c**. The corresponding phenacyl chlorides reacted smoothly to afford products **6a** and **6b** in 62% and 69% yields, whereas compound **6c** was prepared in 76% yield using 2-(chloroacetyl)thiophene as the alkylating agent. This shows that the reaction tolerates a broader class of electrophiles, making it suitable for accessing precursors of TT molecules (Scheme 6).

**Scheme 7:**
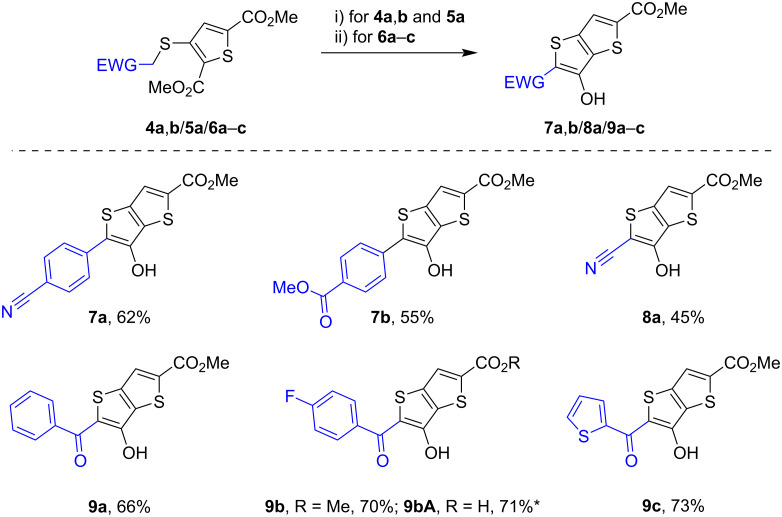
The synthesis of TT derivatives, yields, and scope of products. Conditions: i) LiH (5 equiv), DMF, rt, 24 h. ii) Mg(OMe)_2_ (5 equiv), PhMe/MeOH, 90 °C, 2 h. *NaOMe (2 equiv), MeOH/THF, 75 °C, 2 h.

For the next synthesis of TT derivatives, the base-promoted cyclization of the obtained thiophene-2,5-dicarboxylates **4**–**6** was studied for those derivatives having electron-withdrawing groups in the alkylthio fragment to ensure the closure of the thiophene ring. To establish the optimal conditions for the cyclization of compounds **4**–**6**, a series of exploratory experiments was carried out using various bases and solvents. Initial attempts involved the use of NaOMe in methanol, a simple base–solvent combination for such substrates. However, this system proved unsuitable due to the limited solubility of the starting materials in methanol, which prevented efficient conversion. Subsequently, the substrates were introduced as THF solutions into NaOMe in methanol. Under these conditions, the cyclization did occur to form the corresponding Na salts of 3-hydroxy-TTs. Upon aqueous work-up and acidification, however, the reaction afforded mixtures of the desired products and the corresponding carboxylic acids due to partial hydrolysis of the ester groups. This behavior was observed during cyclization of substrates **4a** and **5a**. In the case of substrate **6b**, treatment with NaOMe resulted primarily in the formation of carboxylic acid **9bA**, isolated in 71% yield (Scheme 7). In addition, an attempt to cyclize **6b** in the presence of DBU without a solvent at 110 °C led to significant destruction of the substrate and product **9b** was obtained with a yield of about 20%. Cyclization of substrate **4a** with NaH in toluene also gave product **7a** in about 25% yield, along with the formation of a mixture of byproducts. It was found that the most favorable conditions for cyclization of compounds **4a**, **4b**, and **5a** were their treatment with excess LiH in a solution of dry DMF at room temperature for 24 h. In this way, products **7a**,**b**, and **8a** were obtained in 62%, 55%, and 45% yields, respectively. In turn, the suitable reaction conditions for cyclization of substrates **6a**–**c** were their treatment with excess Mg(OMe)_2_ in a solution of methanol/toluene, which afforded products **9a**–**c** in 66–73% yield. It should be noted that the bases LiH and Mg(OMe)_2_ chosen for the cyclization are convenient from the point of view of availability and work safety. Indeed, LiH is not a pyrophoric hydride compared to other alkali metal hydrides, NaH or KH, while Mg(OMe)_2_ is easily prepared by dissolving I_2_-activated Mg metal in dry methanol.

## Conclusion

In summary, this study reveals an unusual and previously poorly understood reactivity of 3-nitrothiophene-2,5-dicarboxylate toward sulfur-containing nucleophiles such as potassium thioacetate or thioacetamide, which resulted in the efficient formation of both bis(thiophen-3-yl) sulfide and disulfide derivatives via substitution of the nitro group. This reaction opens a new route to sulfur-containing derivatives of the thiophene nucleus. Beyond enabling an effective synthesis of 3-hydroxy-substituted TTs bearing electron-withdrawing groups at the C-2 position, this work also establishes a robust approach to a wide range of 3-substituted thiophene-2,5-dicarboxylates. These intermediates are of interest as valuable building blocks for the synthesis of more complex thiophene derivatives in both materials and medicinal chemistry contexts.

## Supporting Information

File 1 Full experimental details, characterization data, copies of NMR spectra and HRMS for all new compounds.

## Data Availability

All data that supports the findings of this study is available in the published article and/or the supporting information of this article.
