# Supplementary material for: Assembly strategy for thieno[3,2-b]thiophenes via a disulfide intermediate derived from 3-nitrothiophene-2,5-dicarboxylate
Source: Beilstein J Org Chem. 2025 Nov 11;21:2489–97. doi: 10.3762/bjoc.21.191 (PMC12621641; doi:10.3762/bjoc.21.191)

BEILSTEIN JOURNAL OF ORGANIC CHEMISTRY

Supporting Information

for

Assembly strategy for thieno[3,2-*b*]thiophenes via a disulfide intermediate derived from 3-nitrothiophene-2,5-dicarboxylate

Roman A. Irgashev

Beilstein J. Org. Chem. **2025,** *21,* 2489–2497. doi:10.3762/bjoc.21.191

Full experimental details, characterization data, copies of NMR spectra and HRMS for all new compounds

License and Terms: This is a supporting information file under the terms of the Creative Commons Attribution License (https://creativecommons.org/licenses/by/4.0). Please note that the reuse, redistribution and reproduction in particular requires that the author(s) and source are credited and that individual graphics may be subject to special legal provisions.
The license is subject to the *Beilstein Journal of Organic Chemistry* terms and conditions: (https://www.beilstein-journals.org/bjoc/terms)

Table of contents

1. General information S3

2. Experimental procedures S3

*Procedure for the synthesis of sulfide **2** using thioacetamide and K_2CO_3* S3

*Procedure for the synthesis of disulfide **3** using potassium thioacetate (KSAc)* S4

*Procedure for the synthesis of disulfide **3** using Na_2S_2* S4

*Procedure for one-pot reduction–alkylation of disulfide **3** using $Na_2S_2O_4$ (synthesis of compound **4a**)* S5

*General procedure for one-pot reduction–alkylation of disulfide **3** using $NaBH_4$ (synthesis of compounds **4**–**6**)* S6

*General procedure for the cyclization of compounds **4a**, **4b** and **5a** using LiH* S11

*Procedure for the synthesis of thieno[3,2-b]thiophene-2-carboxylic acid **9bA*** S12

*General procedure for the cyclization of compounds **6a**–**c** using $Mg(OMe)_2$* S13

3. References S15

4. Copies of ^1H, ^{19}F, ^{13}C NMR spectra S16

Tetramethyl 3,3'-thiobis(thiophene-2,5-dicarboxylate) (**2**) S16

Tetramethyl 3,3'-disulfanediylbis(thiophene-2,5-dicarboxylate) (**3**) S18

A mixture of compounds **2** and **3** from the reaction of ester **1** with potassium thioacetate S20

A mixture of compounds **2** and **3** from the reaction of ester **1** with potassium xanthate S21

A mixture of compounds **2** and **3** from the reaction of ester **1** with sodium diethyldithiocarbamate S22

A mixture of compounds **2** and **3** from the reaction of ester **1** with thioacetamide and K_2CO_3 .... S23

A mixture of compounds **2** and **3** from the reaction of ester **1** with Na_2S_2 .... S24

Dimethyl 3-[(4-cyanobenzyl)thio]thiophene-2,5-dicarboxylate (**4a**) S25

Dimethyl 3-{[4-(methoxycarbonyl)benzyl]thio}thiophene-2,5-dicarboxylate (**4b**) S27

Dimethyl 3-(benzylthio)thiophene-2,5-dicarboxylate (**4c**) S29

Dimethyl 3-[(4-fluorobenzyl)thio]thiophene-2,5-dicarboxylate (**4d**) S31

Dimethyl 3-[(3,4-difluorobenzyl)thio]thiophene-2,5-dicarboxylate (**4e**) S34

Dimethyl 3-{[4-(trifluoromethyl)benzyl]thio}thiophene-2,5-dicarboxylate (**4f**) S37

Dimethyl 3-[(benzo[*d*][1,3]dioxol-5-ylmethyl)thio]thiophene-2,5-dicarboxylate (**4g**) S40

Dimethyl 3-[(thiophen-2-ylmethyl)thio]thiophene-2,5-dicarboxylate (**4h**) S42

Dimethyl 3-[(cyanomethyl)thio]thiophene-2,5-dicarboxylate (**5a**) S44

Dimethyl 3-[(2-oxo-2-phenylethyl)thio]thiophene-2,5-dicarboxylate (**6a**) S46

Dimethyl 3-{[2-(4-fluorophenyl)-2-oxoethyl]thio}thiophene-2,5-dicarboxylate (**6b**) S48

Dimethyl 3-{[2-oxo-2-(thiophen-2-yl)ethyl]thio}thiophene-2,5-dicarboxylate (**6c**) S51

Methyl 5-(4-cyanophenyl)-6-hydroxythieno[3,2-*b*]thiophene-2-carboxylate (**7a**) S53

Methyl 6-hydroxy-5-[4-(methoxycarbonyl)phenyl]thieno[3,2-*b*]thiophene-2-carboxylate (**7b**).....S55
Methyl 5-cyano-6-hydroxythieno[3,2-*b*]thiophene-2-carboxylate (**8a**).......................................S57
5-(4-Fluorobenzoyl)-6-hydroxythieno[3,2-*b*]thiophene-2-carboxylic acid (**9bA**) S59
Methyl 5-benzoyl-6-hydroxythieno[3,2-*b*]thiophene-2-carboxylate (**9a**)....................................S62
Methyl 5-(4-fluorobenzoyl)-6-hydroxythieno[3,2-*b*]thiophene-2-carboxylate (**9b**).......................S64
Methyl 6-hydroxy-5-(thiophene-2-carbonyl)thieno[3,2-*b*]thiophene-2-carboxylate (**9c**).............S67
5. Copies of high-resolution mass spectra (HRMS) S70
Tetramethyl 3,3'-thiobis(thiophene-2,5-dicarboxylate) (**2**) S70
Tetramethyl 3,3'-disulfanediylbis(thiophene-2,5-dicarboxylate) (**3**) S70
Dimethyl 3-[(4-cyanobenzyl)thio]thiophene-2,5-dicarboxylate (**4a**)...S70
Dimethyl 3-{[4-(methoxycarbonyl)benzyl]thio}thiophene-2,5-dicarboxylate (**4b**) S71
Dimethyl 3-(benzylthio)thiophene-2,5-dicarboxylate (**4c**)..S71
Dimethyl 3-[(4-fluorobenzyl)thio]thiophene-2,5-dicarboxylate (**4d**) S71
Dimethyl 3-[(3,4-difluorobenzyl)thio]thiophene-2,5-dicarboxylate (**4e**)......................................S71
Dimethyl 3-{[4-(trifluoromethyl)benzyl]thio}thiophene-2,5-dicarboxylate (**4f**) S72
Dimethyl 3-[(benzo[*d*][1,3]dioxol-5-ylmethyl)thio]thiophene-2,5-dicarboxylate (**4g**)...................S72
Dimethyl 3-[(thiophen-2-ylmethyl)thio]thiophene-2,5-dicarboxylate (**4h**) S72
Dimethyl 3-[(cyanomethyl)thio]thiophene-2,5-dicarboxylate (**5a**) S72
Dimethyl 3-[(2-oxo-2-phenylethyl)thio]thiophene-2,5-dicarboxylate (**6a**) S73
Dimethyl 3-{[2-(4-fluorophenyl)-2-oxoethyl]thio}thiophene-2,5-dicarboxylate (**6b**) S73
Dimethyl 3-{[2-oxo-2-(thiophen-2-yl)ethyl]thio}thiophene-2,5-dicarboxylate (**6c**) S73
Methyl 5-(4-cyanophenyl)-6-hydroxythieno[3,2-*b*]thiophene-2-carboxylate (**7a**) S73
Methyl 6-hydroxy-5-[4-(methoxycarbonyl)phenyl]thieno[3,2-*b*]thiophene-2-carboxylate (**7b**).....S74
Methyl 5-cyano-6-hydroxythieno[3,2-*b*]thiophene-2-carboxylate (**8a**).......................................S74
5-(4-Fluorobenzoyl)-6-hydroxythieno[3,2-*b*]thiophene-2-carboxylic acid (**9bA**) S74
Methyl 5-benzoyl-6-hydroxythieno[3,2-*b*]thiophene-2-carboxylate (**9a**)....................................S74
Methyl 5-(4-fluorobenzoyl)-6-hydroxythieno[3,2-*b*]thiophene-2-carboxylate (**9b**).......................S75
Methyl 6-hydroxy-5-(thiophene-2-carbonyl)thieno[3,2-*b*]thiophene-2-carboxylate (**9c**).............S75

1. General information

^{1}H, ^{13}C, and ^{19}F NMR spectra of the synthesized compounds were recorded on NMR spectrometers in $CDCl_3$ or DMSO-d_6 with $SiMe_4$ as an internal standard for ^{1}H and ^{13}C NMR spectra, and C_6F_6 as an internal standard for ^{19}F NMR. Mass spectra were obtained on a Q-TOF LCMS/MS spectrometer. Elemental analyses were performed on an automated CHNS analyzer. Melting points were determined on combined heating stages and were not corrected. Dimethyl 3-nitrothiophene-2,5-dicarboxylate (**1**) was prepared using our previously reported procedure [1]. The alkylating agents for the preparation of compounds **4g** and **4h**, respectively, 3,4-methylenedioxybenzyl and thiophen-2-ylmethyl mesylates, were synthesized by treatment of the corresponding benzyl alcohols with methanesulfonyl chloride [2], for the preparation of compound **6c**, 2-(chloroacetyl)thiophene was prepared by chlorination of 2-acetylthiophene with SO_2Cl_2 [3]. Unless otherwise stated, all reagents were purchased from commercial suppliers and used without further purification.

2. Experimental procedures

*Procedure for the synthesis of sulfide **2** using thioacetamide and K_2CO_3*

Thioacetamide (0.90 g, 12 mmol) and K_2CO_3 (1.66 g, 12 mmol) were added to a solution of ester **1** (2.45 g, 10 mmol) in acetone (20 mL). The suspension was stirred and heated at reflux (oil bath temperature 70 °C) for 1 h, then diluted with water (30 mL). The precipitate was filtered, washed with acetone/water (2 × 10 mL, v/v, 1:1) and water (10 mL), and dried at 110 °C. The crude matter was

recrystallized from DMF/methanol (35 mL, v/v 6:1) and washed with methanol (10 mL) on filter, dried at 110 °C to yield analytically pure sulfide **2**.

Tetramethyl 3,3'-thiobis(thiophene-2,5-dicarboxylate) (**2**): Yellow needles, yield 1.09 g (51%), mp 216-217 °C. ^{1}H NMR (500 MHz, $CDCl_3$) δ 7.40 (s, 1H), 3.91 (s, 3H), 3.89 (s, 3H). ^{13}C NMR (126 MHz, $CDCl_3$) δ 161.3, 161.2, 138.5, 137.0, 135.5, 133.0, 52.7, 52.6. HRMS (ESI) calcd for $C_{16}H_{15}O_8S_3$ m/z 430.9924 $[M+H]^+$, found m/z 430.9925 $[M+H]^+$. Anal. calcd for $C_{16}H_{14}O_8S_3$: C, 44.64; H, 3.28; S, 22.34. Found: C, 44.72; H, 3.21; S, 22.25

*Procedure for the synthesis of disulfide **3** using potassium thioacetate (KSAc)*

Ester **1** (4.90 g, 20 mmol) was dissolved in acetone (40 mL), and KSAc (2.51 g, 22 mmol) was added at ambient temperature. The reaction mixture was stirred and heated at reflux (oil bath temperature 70 °C) for 1 h. The resulting suspension was diluted with water (40 mL), and the precipitate was collected by filtration, washed with acetone/water (5 × 5 mL, v/v 1:1) and water (15 mL), and dried at 110 °C. The crude substance was recrystallized twice from toluene (45 mL, then 30 mL) and washed with methanol (5 mL) on filter to afford analytically pure disulfide **3**, yield 3.47 g (75%).

*Procedure for the synthesis of disulfide **3** using Na_2S_2*

Sulfur powder (1.28 g, 40 mmol) was added to a solution of $Na_2S{\cdot}9H_2O$ (9.60 g, 40 mmol) in DMF/water (80 mL, v/v 1:1), and the suspension was heated at 100 °C in the oil bath for 1 h until complete dissolution of sulfur, forming a deep-yellow Na_2S_2 solution. After cooling to ambient temperature, this solution was added

dropwise to a cooled (0–5 °C) solution of ester **1** (18.60 g, 75.9 mmol) in acetone (200 mL). The reaction mixture, which turned dark red, was stirred and heated at reflux (oil bath temperature 70 °C) for 1 h. The precipitate was collected by filtration, washed with warm water (100 mL), and dried at 110 °C. Recrystallization from toluene/DMF (125 mL, v/v 60:1) afforded analytically pure disulfide **3**, yield 12.4 g (71%).

Tetramethyl 3,3'-disulfanediylbis(thiophene-2,5-dicarboxylate) (**3**): Light-beige crystals, mp 212-213 °C (toluene-DMF). ^{1}H NMR (400 MHz, $CDCl_3$) δ 7.83 (s, 1H), 3.97 (s, 3H), 3.87 (s, 3H). ^{13}C NMR (126 MHz, $CDCl_3$) δ 162.1, 161.4, 143.3, 137.3, 132.1, 128.2, 52.8. HRMS (ESI) calcd for $C_{16}H_{15}O_8S_4$ m/z 462.9644 $[M+H]^+$, found m/z 462.9646 $[M+H]^+$. Anal. calcd for $C_{16}H_{14}O_8S_4$: C, 41.55; H, 3.05; S, 27.73 Found: C, 41.63; H, 2.94; S, 27.27.

*Procedure for one-pot reduction–alkylation of disulfide **3** using $Na_2S_2O_4$ (synthesis of compound **4a**)*

Disulfide **3** (185 mg, 0.4 mmol) was dissolved in DMF (9.0 mL)/water (1.0 mL), and K_2CO_3 (166 mg, 1.20 mmol) and $Na_2S_2O_4$ (209 mg, 1.2 mmol) were added. After stirring for 5 min at ambient temperature, 4-(chloromethyl)benzonitrile (133 mg, 0.88 mmol) was added and the mixture was stirred at ambient temperature for 24 h. The reaction mixture was then poured into water (20 mL), and the precipitate formed was filtered off, washed with water (2 × 5 mL) and methanol (2 mL), and dried at 60 °C. Product **4a** was obtained in analytically pure form without additional purification. Yield 167 mg (60%).

*General procedure for one-pot reduction–alkylation of disulfide **3** using $NaBH_4$ (synthesis of compounds **4**–**6**)*

$NaBH_4$ (95 mg, 2.5 mmol) was added to a suspension of disulfide **3** (463 mg, 1 mmol) in DMF (15 mL) under argon at ambient temperature. The mixture was heated at 75 °C in the oil bath for 15 min until complete dissolution to give a deep orange solution. Methanol (5 mL) was added, and the solution was stirred at ambient temperature for 10 min. An alkylating agent (2.2 mmol) was added, causing decolorization of the reaction mixture. The reaction was stirred for 20 min, diluted with water (15 mL), and the precipitate was collected, washed with water (15 mL) and methanol (2 × 5 mL), and dried at 60 °C. Products **4**–**6** were obtained in analytically pure form without further purification. For compound **5a**, the mixture was diluted with water (60 mL), the formed precipitate was filtered and washed with water (20 mL), then dried.

Dimethyl 3-[(4-cyanobenzyl)thio]thiophene-2,5-dicarboxylate (**4a**): White microcrystals, yield 612 mg (88%), mp 195-196 °C. ^1H NMR (400 MHz, $CDCl_3$) δ 7.67 – 7.60 (m, 1H), 7.57 (s, 1H), 7.55 – 7.50 (m, 1H), 4.28 (s, 1H), 3.90 (s, 3H). ^{13}C NMR (126 MHz, $CDCl_3$) δ 161.9, 161.5, 142.4, 141.5, 137.2, 132.7, 132.5, 131.6, 131.2, 129.6, 127.9, 118.5, 111.6, 52.8, 52.4, 37.4. HRMS (ESI) calcd for $C_{16}H_{12}O_4NS_2$ m/z 346.0213 $[M–H]^–$, found m/z 346.0212 $[M–H]^–$. 4-(Chloromethyl)benzonitrile (334 mg, 2.2 mmol) was used as the alkylating agent.

Dimethyl 3-{[4-(methoxycarbonyl)benzyl]thio}thiophene-2,5-dicarboxylate (**4b**): White powder, yield 632 mg (83%), mp 108-109 °C. ^1H NMR (400 MHz, $CDCl_3$) δ

8.00 (d, J = 8.4 Hz, 2H), 7.60 (s, 1H), 7.49 (d, J = 8.4 Hz, 2H), 4.29 (s, 2H), 3.91 (s, 3H), 3.90 (s, 6H). ^{13}C NMR (126 MHz, $CDCl_3$) δ 166.6, 161.9, 161.5, 143.2, 141.1, 137.0, 131.3, 130.0, 129.5, 128.9, 127.5, 52.7, 52.4, 52.1, 37.5. HRMS (ESI) calcd for $C_{17}H_{15}O_6S_2$ m/z 379.0316 $[M–H]^–$, found m/z 379.0313 $[M–H]^–$. Methyl 4-(bromomethyl)benzoate (504 mg, 2.2 mmol) was used as the alkylating agent.

Dimethyl 3-(benzylthio)thiophene-2,5-dicarboxylate (**4c**): White powder, yield 490 mg (76%), mp 146-147 °C. ^{1}H NMR (400 MHz, $CDCl_3$) δ 7.66 (s, 1H), 7.44 – 7.39 (m, 2H), 7.37 – 7.27 (m, 3H), 4.26 (s, 2H), 3.90 (s, 3H), 3.89 (s, 3H). ^{13}C NMR (126 MHz, $CDCl_3$) δ 162.0, 161.6, 144.0, 136.9, 135.6, 131.3, 128.9, 128.7, 127.6, 127.0, 52.6 52.3, 37.8. HRMS (ESI) calcd for $C_{15}H_{15}O_4S_2$ m/z 323.0406 $[M+H]^+$, found m/z 323.0407 $[M+H]^+$. Benzyl chloride (280 mg, 2.2 mmol) was used as the alkylating agent.

Dimethyl 3-[(4-fluorobenzyl)thio]thiophene-2,5-dicarboxylate (**4d**): White microcrystals, yield 560 mg (82%), mp 137-138 °C. ^{1}H NMR (500 MHz, $CDCl_3$) δ 7.63 (s, 1H), 7.41 – 7.34 (m, 2H), 7.06 – 6.97 (m, 2H), 4.23 (s, 2H), 3.90 (s, 3H), 3.89 (s, 3H). ^{19}F NMR (471 MHz, $CDCl_3$) δ 47.35 – 47.24 (m). ^{13}C NMR (126 MHz $CDCl_3$) δ 162.2 (d, J_{CF} = 246.6 Hz), 162.0, 161.6, 143.6, 137.0, 131.4 (d, J_{CF} = 3.2 Hz), 131.2, 130.5 (d, J_{CF} = 8.2 Hz), 127.2, 115.6 (d, J_{CF} = 21.6 Hz), 52.7, 52.3, 37.1. HRMS (ESI) calcd for $C_{15}H_{13}O_4FS_2$ m/z 341.0312 $[M+H]^+$, found m/z

341.0319 [M+H]$^+$. 4-Fluorobenzyl chloride (320 mg, 2.2 mmol) was used as the alkylating agent.

Dimethyl 3-[(3,4-difluorobenzyl)thio]thiophene-2,5-dicarboxylate (**4e**): White powder, yield 631 mg (88%), mp 139-140 °C. ^{1}H NMR (400 MHz, DMSO-d_6) δ 7.84 (s, 1H), 7.52 (ddd, *J* = 11.6, 7.8, 2.0 Hz, 2H), 7.42 (dt, *J* = 10.7, 8.5 Hz, 1H), 7.30 (dq, *J* = 6.6, 2.0 Hz, 2H), 4.46 (s, 2H), 3.87 (s, 3H), 3.82 (s, 3H). ^{19}F NMR (376 MHz, $CDCl_3$) δ 25.06 – 24.77 (m), 23.04 – 22.76 (m). ^{13}C NMR (126 MHz, $CDCl_3$) δ 161.9, 161.5, 151.1 (dd, *J* = 64.4, 12.7 Hz), 149.1 (dd, *J* = 64.0, 12.7 Hz), 142.9, 137.1, 132.8 (dd, *J* = 5.6, 3.9 Hz), 131.2, 127.6, 124.9 (dd, *J* = 6.3, 3.6 Hz), 117.8 (d, *J* = 17.8 Hz), 117.5 (d, *J* = 17.4 Hz), 52.7, 52.4, 36.8. HRMS (ESI) calcd for $C_{15}H_{11}F_2O_4S_2$ m/z 357.0072 [M–H]$^-$, found m/z 357.0074 [M–H]$^-$. 3,4-Difluorobenzyl chloride (360 mg, 2.2 mmol) was used as the alkylating agent.

Dimethyl 3-{[4-(trifluoromethyl)benzyl]thio}thiophene-2,5-dicarboxylate (**4f**): White microcrystals, yield 617 mg (79%), mp 89-90 °C. ^{1}H NMR (400 MHz, $CDCl_3$) δ 7.61, 7.60, 7.58, 7.54, 7.52, 7.26, 4.29, 3.90, 3.90, -0.00. ^{19}F NMR (376 MHz, $CDCl_3$) δ 99.13. ^{13}C NMR (126 MHz, $CDCl_3$) δ 161.9, 161.5, 142.9, 134.0, 137.1, 131.2, 129.9 (q, *J* = 32.6 Hz), 129.2, 127.6, 125.7 (q, *J* = 3.7 Hz), 124.0 (q, *J* = 272.1 Hz), 52.7, 52.4, 37.3. HRMS (ESI) calcd for $C_{16}H_{12}F_3O_4S_2$ m/z 389.0135 [M–H]$^-$, found m/z 389.0136 [M–H]$^-$. 4-(Trifluoromethyl)benzyl chloride (430 mg, 2.2 mmol) was used as the alkylating agent.

Dimethyl 3-[(benzo[*d*][1,3]dioxol-5-ylmethyl)thio]thiophene-2,5-dicarboxylate (**4g**): White powder, yield 674 mg (92%), mp 177-178 °C. ^{1}H NMR (500 MHz, $CDCl_3$) δ 7.65 (s, 1H), 6.90 (d, *J* = 1.7 Hz, 1H), 6.86 (dd, *J* = 7.9, 1.7 Hz, 1H), 6.75 (d, *J* = 7.9 Hz, 1H), 5.95 (s, 2H), 4.18 (s, 2H), 3.90 (s, 3H), 3.89 (s, 3H). ^{13}C NMR (126 MHz, $CDCl_3$) δ 162.0, 161.7, 147.9, 147.2, 144.0, 136.9, 131.3, 129.2, 127.1, 122.3, 109.2, 108.3, 101.1, 52.7, 52.3, 37.8. HRMS (ESI) calcd for $C_{16}H_{15}O_6S_2$ m/z 367.0305 $[M+H]^+$, found m/z 367.0307 $[M+H]^+$. 3,4-Methylenedioxybenzyl mesylate (470 mg, 2.2 mmol) was used as the alkylating agent.

Dimethyl 3-[(thiophen-2-ylmethyl)thio]thiophene-2,5-dicarboxylate (**4h**): White crystals, yield 506 mg (77%), mp 122-123 °C. ^{1}H NMR (500 MHz, $CDCl_3$) δ 7.66 (s, 1H), 7.22 (dd, *J* = 5.1, 1.1 Hz, 1H), 7.08 – 7.02 (m, 2H), 6.94 (dd, *J* = 5.1, 3.5 Hz, 1H), 4.46 (s, 3H), 3.90 (s, 6H). ^{13}C NMR (126 MHz, $CDCl_3$) δ 161.9, 161.6, 143.1, 138.9, 137.0, 132.1, 131.3, 127.0, 126.8, 125.4, 52.7, 52.4, 32.2. HRMS (ESI) calcd for $C_{13}H_{12}O_4S_3$ m/z 328.9970 $[M+H]^+$, found m/z 328.9971 $[M+H]^+$. Thiophen-2-ylmethyl mesylate (425 mg, 2.2 mmol) was used as the alkylating agent.

Dimethyl 3-[(cyanomethyl)thio]thiophene-2,5-dicarboxylate (**5a**): White woolly crystals, yield 260 mg (48%) with $ClCH_2CN$, 495 mg (91%) with $BrCH_2CN$, mp 135-138 °C. ^{1}H NMR (400 MHz, $CDCl_3$) δ 7.71 (s, 1H), 3.94 (d, *J* = 4.4 Hz, 5H), 3.79 (s, 2H). ^{13}C NMR (126 MHz, $CDCl_3$) **δ** 161.6, 161.2, 138.5, 138.0, 131.2, 129.9, 115.7, 52.9, 52.7, 18.7. HRMS (ESI) calcd for $C_{10}H_8NO_4S_2$ m/z 269.9900 $[M–H]^–$,

found m/z 269.9901 [M–H]⁻. Chloroacetonitrile (170 mg, 2.2 mmol) or bromoacetonitrile (265 mg, 2.2 mmol) was used as the alkylating agent.

Dimethyl 3-[(2-oxo-2-phenylethyl)thio]thiophene-2,5-dicarboxylate (**6a**): White powder, yield 505 mg (72%), mp 126-127 °C. ^{1}H NMR (400 MHz, $CDCl_3$) δ 8.03 – 7.98 (m, 1H), 7.75 (s, 1H), 7.65 – 7.58 (m, 1H), 7.53 – 7.46 (m, 1H), 4.43 (s, 1H), 3.91 (s, 1H), 3.89 (s, 1H). ^{13}C NMR (126 MHz, $CDCl_3$) δ 193.2, 161.9, 161.6, 142.0, 137.1, 135.0, 133.8, 131.8, 128.8, 128.7, 128.0, 52.7, 52.4, 39.4. HRMS (ESI) calcd for $C_{16}H_{13}O_5S_2$ m/z 349.0210 $[M–H]^-$, found m/z 349.0207 $[M–H]^-$. Phenacyl chloride (340 mg, 2.2 mmol) was used as the alkylating agent.

Dimethyl 3-{[2-(4-fluorophenyl)-2-oxoethyl]thio}thiophene-2,5-dicarboxylate (**6b**): White microcrystals, yield 508 mg (69%), mp 154-155 °C. ^{1}H NMR (400 MHz, $CDCl_3$) δ 8.07 – 7.99 (m, 1H), 7.76 (s, 1H), 7.21 – 7.12 (m, 1H), 4.39 (s, 1H), 3.92 (s, 2H), 3.89 (s, 2H). ^{19}F NMR (376 MHz, $CDCl_3$) δ 58.27 (tt, J = 8.3, 5.3 Hz). ^{13}C NMR (126 MHz, $CDCl_3$) δ 191.8, 166.1 (d, J_{CF} = 256.4 Hz), 161.9, 161.6, 141.7, 137.2, 131.8, 131.5, 131.4, 128.2, 116.0 (d, J_{CF} = 22.0 Hz), 52.7, 52.4, 39.3. HRMS (ESI) calcd for $C_{16}H_{12}FO_5S_2$ m/z 367.0116 $[M–H]^-$, found m/z 367.0116 $[M–H]^-$. 4-Fluorophenacyl chloride (380 mg, 2.2 mmol) was used as the alkylating agent.

Dimethyl 3-{[2-oxo-2-(thiophen-2-yl)ethyl]thio}thiophene-2,5-dicarboxylate (**6c**): Cream powder, yield 542 mg (76%), mp 128 -129 °C. ^{1}H NMR (400 MHz, $CDCl_3$) δ 7.86 – 7.82 (m, 1H), 7.80 (s, 1H), 7.71 (dd, J = 4.9, 0.6 Hz, 1H), 7.17 (dd, J =

4.7, 4.1 Hz, 1H), 4.32 (s, 2H), 3.91 (s, 4H), 3.89 (s, 3H). ^{13}C NMR (126 MHz, $CDCl_3$) δ 186.4, 161.9, 161.6, 141.9, 141.9, 137.2, 134.9, 133.2, 131.9, 128.3, 127.9, 52.7, 52.4, 39.7. HRMS (ESI) calcd for $C_{14}H_{12}O_5S_3$ m/z 354.9774 [M–H]$^-$, found m/z 354.9773 [M–H]$^-$. 2-(Chloroacetyl)thiophene (355 mg, 2.2 mmol) was used as an alkylating agent.

General procedure for the cyclization of compounds ***4a****,* ***4b*** *and* ***5a*** *using LiH*

Freshly ground LiH (40 mg, 5 mmol) was added to a solution of substrate **4a**, **4b** or **5a** (1 mmol) in dry DMF (8 mL) at ambient temperature. The mixture was stirred for 24 h, then carefully poured into water (40 mL) containing glacial acetic acid (5 mL). The precipitate was filtered, washed with water (3 × 10 mL), and dried at 110 °C. Recrystallization from 1,4-dioxane (5 mL) with methanol washes (2 × 5 mL) afforded analytically pure products **7a**, **7b** or **8a**.

Methyl 5-(4-cyanophenyl)-6-hydroxythieno[3,2-*b*]thiophene-2-carboxylate (**7a**): Light-yellow woolly crystals, yield 195 mg (62%), mp 275-276 °C. ^{1}H NMR (400 MHz, DMSO-d_6) δ 11.48 (br.s, 1H), 8.15 (s, 1H), 8.04 – 7.94 (m, 2H), 7.92 – 7.75 (m, 2H), 3.87 (s, 3H). ^{13}C NMR (126 MHz, DMSO-d_6) δ 162.1, 146.0, 138.4, 135.6, 134.9, 134.4, 132.7, 127.3, 126.2, 126.1, 119.8, 119.0, 108.3, 52.5. HRMS (ESI) calcd for $C_{15}H_8NO_3S_2$ m/z 313.9951 [M–H]$^-$, found m/z 313.9951 [M–H]$^-$.

Methyl 6-hydroxy-5-[4-(methoxycarbonyl)phenyl]thieno[3,2-*b*]thiophene-2-carboxylate (**7b**): Light-yellow powder, yield 192 mg (55%), mp 266-267 °C. ^{1}H NMR (400 MHz, DMSO-d_6) δ 11.28 (s, 1H), 8.14 (s, 1H), 8.03 – 7.97 (m, 2H), 7.97

– 7.92 (m, 2H), 3.87 (s, 3H), 3.86 (s, 3H). ^{13}C NMR (126 MHz, DMSO-d_6) δ 165.9, 162.1, 145.4, 138.4, 135.8, 134.5, 134.0, 129.7, 127.2, 127.1, 125.8, 120.7, 52.5, 52.0. HRMS (ESI) calcd for $C_{16}H_{11}O_5S_2$ m/z 347.0053 [M–H]$^-$, found m/z 347.0050 [M–H]$^-$.

Methyl 5-cyano-6-hydroxythieno[3,2-*b*]thiophene-2-carboxylate (**8a**): Light-cream crystals, yield 108 mg (45%), mp 250-251 °C dec. ^{1}H NMR (400 MHz, DMSO-d_6) δ 12.75 (br.s, 1H), 8.18 (s, 1H), 3.88 (s, 3H). ^{13}C NMR (126 MHz, DMSO-d_6) δ 161.7, 156.9, 139.5, 138.5, 131.8, 127.5, 114.0, 87.7, 52.8. HRMS (ESI) calcd for $C_9H_4NO_3S_2$ m/z 237.9638 [M–H]$^-$, found m/z 237.9643 [M–H]$^-$.

*Procedure for the synthesis of thieno[3,2-b]thiophene-2-carboxylic acid **9bA***

A solution of compound **6b** (370 mg, 1.00 mmol) in THF (5 mL) was added to a solution of NaOMe (110 mg, 2.00 mmol) in methanol (5 mL). The reaction mixture was stirred and heated at reflux (oil bath temperature 75 °C) for 2 h, then diluted with water (10 mL) and neutralized with 38% aq. HCl (0.4 mL). The resulting precipitate was filtered off, washed with water (2 × 10 mL), and dried at 110 °C. The crude substance was recrystallized from ethanol (10 mL) to afford analytically pure product **9bA**, yield 229 mg (71%).

5-(4-Fluorobenzoyl)-6-hydroxythieno[3,2-*b*]thiophene-2-carboxylic acid (**9bA**): Light-yellow woolly crystals, mp 315-316 °C. ^{1}H NMR (400 MHz, DMSO-d_6) δ 13.69 (br.s, 1H), 12.07 (br.s, 1H), 8.10 (s, 1H), 8.04 – 7.76 (m, 2H), 7.48 – 7.14

(m, 2H). ^{19}F NMR (376 MHz, DMSO-d_6) δ 55.33 (tt, J = 8.9, 5.5 Hz). ^{13}C NMR (126 MHz, DMSO-d_6) δ 187.6, 164.3 (d, J_{CF} = 250.3 Hz), 162.7, 153.9, 141.2, 140.9, 134.6 (d, J_{CF} = 2.8 Hz), 133.2, 131.4 (d, J_{CF} = 9.2 Hz), 126.8, 119.5, 115.2 (d, J_{CF} = 21.9 Hz). HRMS (ESI) calcd for $C_{14}H_6FO_4S_2$ m/z 320.9697 [M–H]$^-$, found m/z 320.9696 [M–H]$^-$.

*General procedure for the cyclization of compounds **6a**–**c** using Mg(OMe)$_2$*

Mg shavings (122 mg, 5 mmol) were activated by heating with iodine (5 mg) at 110 °C under argon for 15 min, then methanol (10 mL) was added. The mixture was stirred at ambient temperature for 5 min, then heated at reflux (oil bath temperature 75 °C) for 30 min until dissolution of metal. A solution of substrate **6a**, **6b** or **6c** (1 mmol) in toluene (5 mL) was added to the formed Mg(OMe)$_2$ in methanol. The reaction was stirred at reflux (oil bath temperature 90 °C) for 2 h, cooled, and treated with 85% formic acid (1 mL), diethyl ether (20 mL), and water (20 mL). The organic layer was separated, washed with water (30 mL), dried over Na_2SO_4, and evaporated under reduced pressure. The solid residue was recrystallized from 1,4-dioxane (10 mL) and washed with methanol (5 mL) to yield analytically pure products **9a**–**c**.

Methyl 5-benzoyl-6-hydroxythieno[3,2-*b*]thiophene-2-carboxylate (**9a**): Orange needles, yield 210 mg (66%), mp 203-204 °C. ^{1}H NMR (400 MHz, DMSO-d_6) δ 12.06 (br.s, 1H), 8.21 (s, 1H), 7.94 – 7.75 (m, 2H), 7.68 – 7.60 (m, 1H), 7.58 – 7.49 (m, 2H), 3.89 (s, 3H). ^{13}C NMR (126 MHz, DMSO-d_6) δ 189.1, 161.6, 154.2, 141.1,

138.5, 138.1, 133.3, 132.2, 128.4, 128.2, 127.5, 119.6, 52.7. HRMS (ESI) calcd for $C_{15}H_9O_4S_2$ m/z 316.9948 $[M–H]^–$, found m/z 316.9946 $[M–H]^–$.

Methyl 5-(4-fluorobenzoyl)-6-hydroxythieno[3,2-*b*]thiophene-2-carboxylate (**9b**): Light-yellow scaly crystals, yield 235 mg (70%), mp 239-240 °C. ^{1}H NMR (400 MHz, DMSO-d_6) δ 12.07 (br.s, 1H), 8.19 (s, 1H), 8.02 – 7.77 (m, 2H), 7.43 – 7.13 (m, 2H), 3.89 (s, 3H). ^{19}F NMR (376 MHz, DMSO-d_6) δ 55.39 (tt, J = 8.9, 5.5 Hz). ^{13}C NMR (126 MHz, DMSO-d_6) δ 187.5, 164.4 (d, J_{CF} = 250.3 Hz), 161.7, 153.3, 141.0, 138.4, 134.6, 133.5, 131.5 (d, J_{CF} = 9.3 Hz), 127.5, 120.3, 115.2 (d, J_{CF} = 21.9 Hz), 52.8. HRMS (ESI) calcd for $C_{15}H_8FO_4S_2$ m/z 334.9854 $[M–H]^–$, found m/z 334.9851 $[M–H]^–$.

Methyl 6-hydroxy-5-(thiophene-2-carbonyl)thieno[3,2-*b*]thiophene-2-carboxylate (**9c**): Dark orange needles, yield 237 mg (73%), mp 225-226 °C. ^{1}H NMR (400 MHz, $CDCl_3$) δ 13.15 (s, 1H), 8.07 (d, J = 3.8 Hz, 1H), 7.94 (s, 1H), 7.77 (d, J = 4.9 Hz, 1H), 7.27 – 7.21 (m, 1H), 3.96 (s, 3H). ^{1}H NMR (500 MHz, DMSO-d_6) δ 12.41 (br.s, 1H), 8.24 (s, 1H), 8.20 (d, J = 3.7 Hz, 1H), 8.11 (d, J = 4.9 Hz, 1H), 7.32 (dd, J = 4.8, 4.0 Hz, 1H), 3.90 (s, 3H). ^{13}C NMR (126 MHz, DMSO-d_6) δ 179.8, 161.6, 155.2, 142.1, 140.8, 138.7, 135.3, 133.5, 133.1, 128.7, 127.5, 117.6, 52.8. HRMS (ESI) calcd for $C_{13}H_7O_4S_3$ m/z 322.9512 $[M–H]^–$, found m/z 322.9512 $[M–H]^–$.

3. References

(1) Irgashev, R. A.; Kazin, N. A. *Organics* **2024**, *5* (4), 507–519. doi:10.3390/org5040027

(2) Liu, Z.; Zi, Y.; Zhang, L.; Fang, Y.; Shen, Y.; Li, Y.; Yang, X.; Zhang, H. *Chinese J. Org. Chem.* **2020**, *40* (3), 669. doi:10.6023/CJOC201909042

(3) Tu, D.; Luo, J.; Jiang, W.; Tang, Q. *Tetrahedron Lett.* **2021**, *81*, 153335. doi:10.1016/J.TETLET.2021.153335

4. Copies of ^{1}H, ^{19}F, ^{13}C NMR spectra

Tetramethyl 3,3'-thiobis(thiophene-2,5-dicarboxylate) (**2**)

^{1}H NMR (500 MHz, $CDCl_3$)

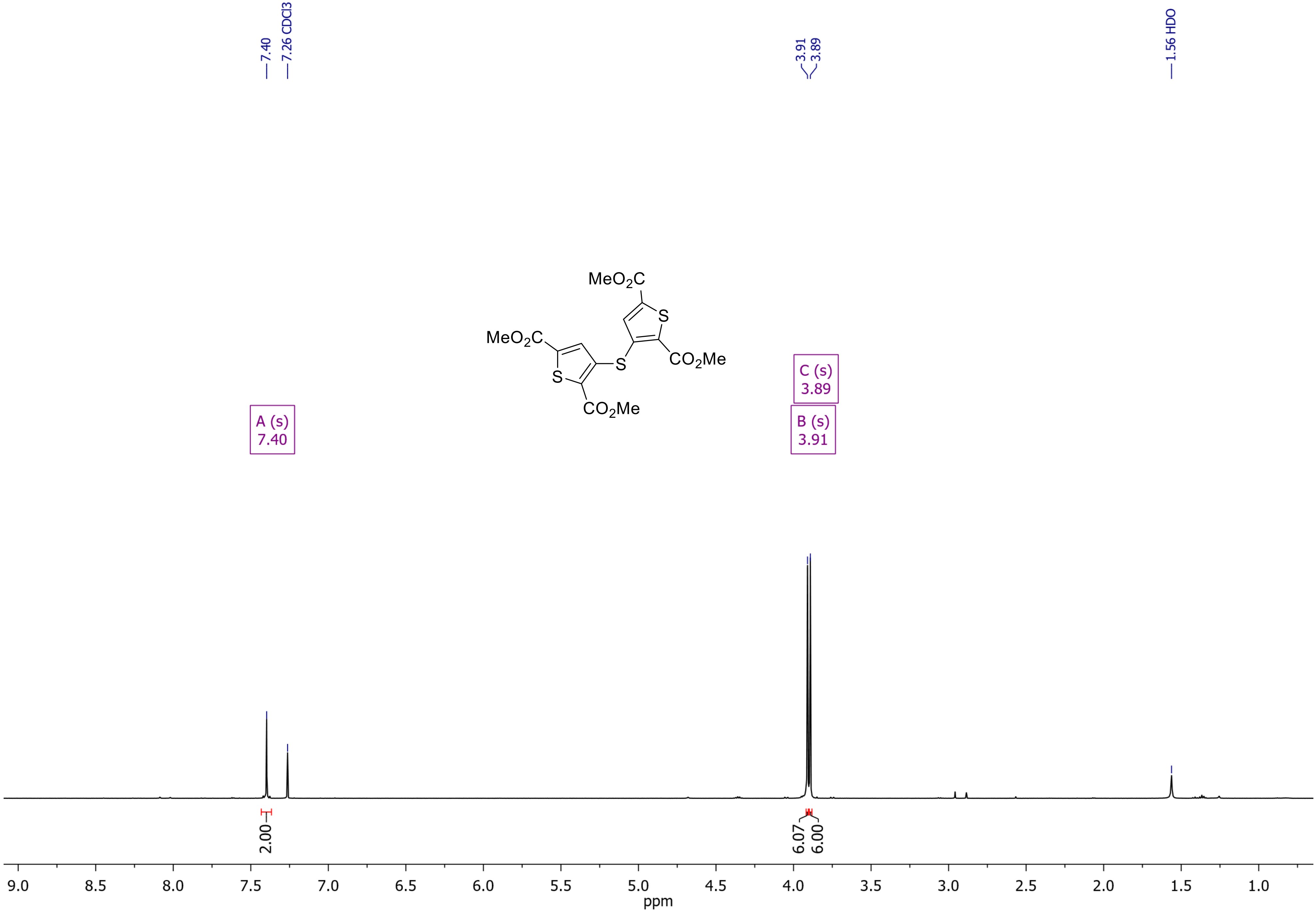

Tetramethyl 3,3'-thiobis(thiophene-2,5-dicarboxylate) (**2**)
^{13}C NMR (126 MHz, $CDCl_3$)

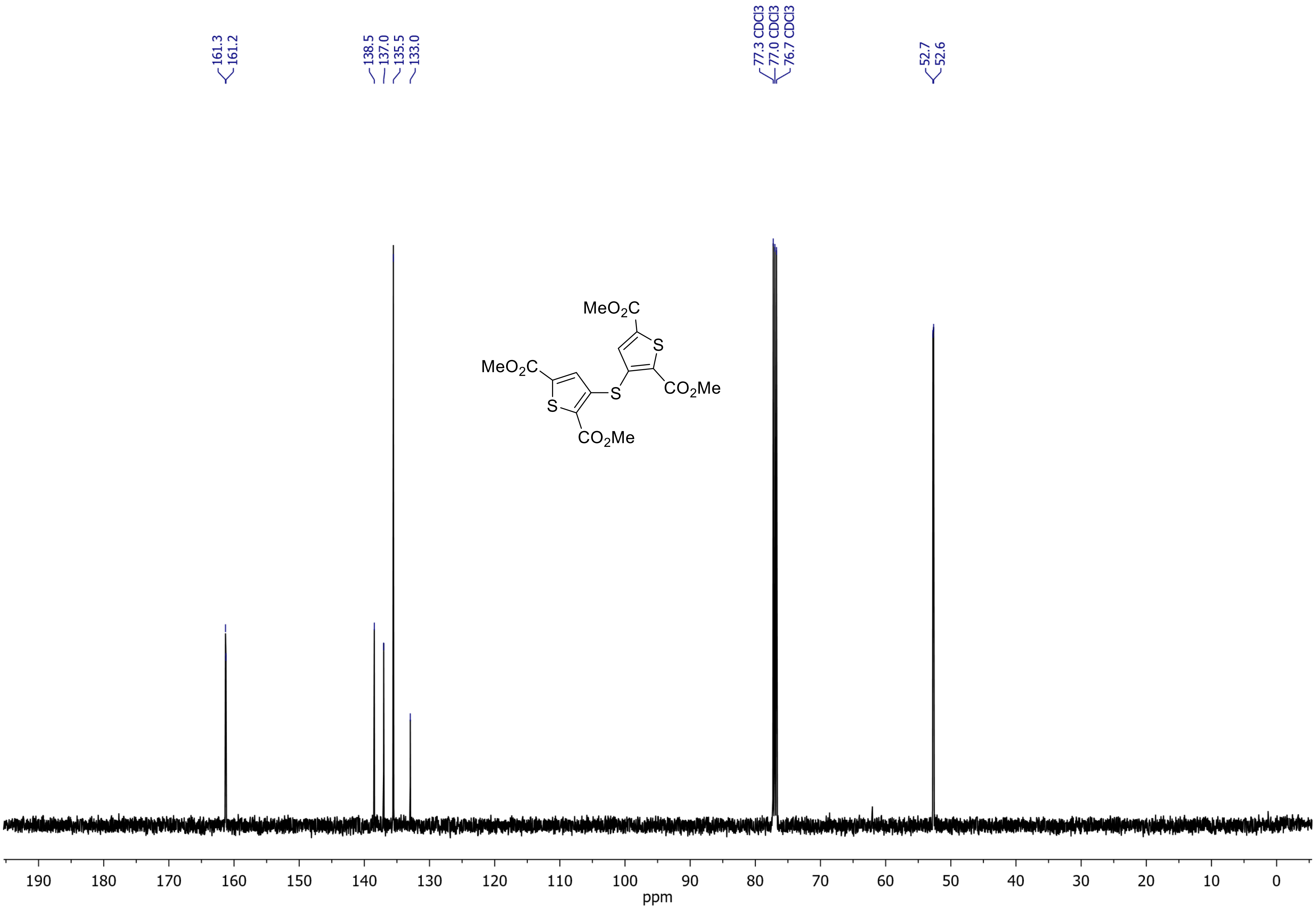

Tetramethyl 3,3'-disulfanediylbis(thiophene-2,5-dicarboxylate) (**3**)

^{1}H NMR (400 MHz, $CDCl_3$)

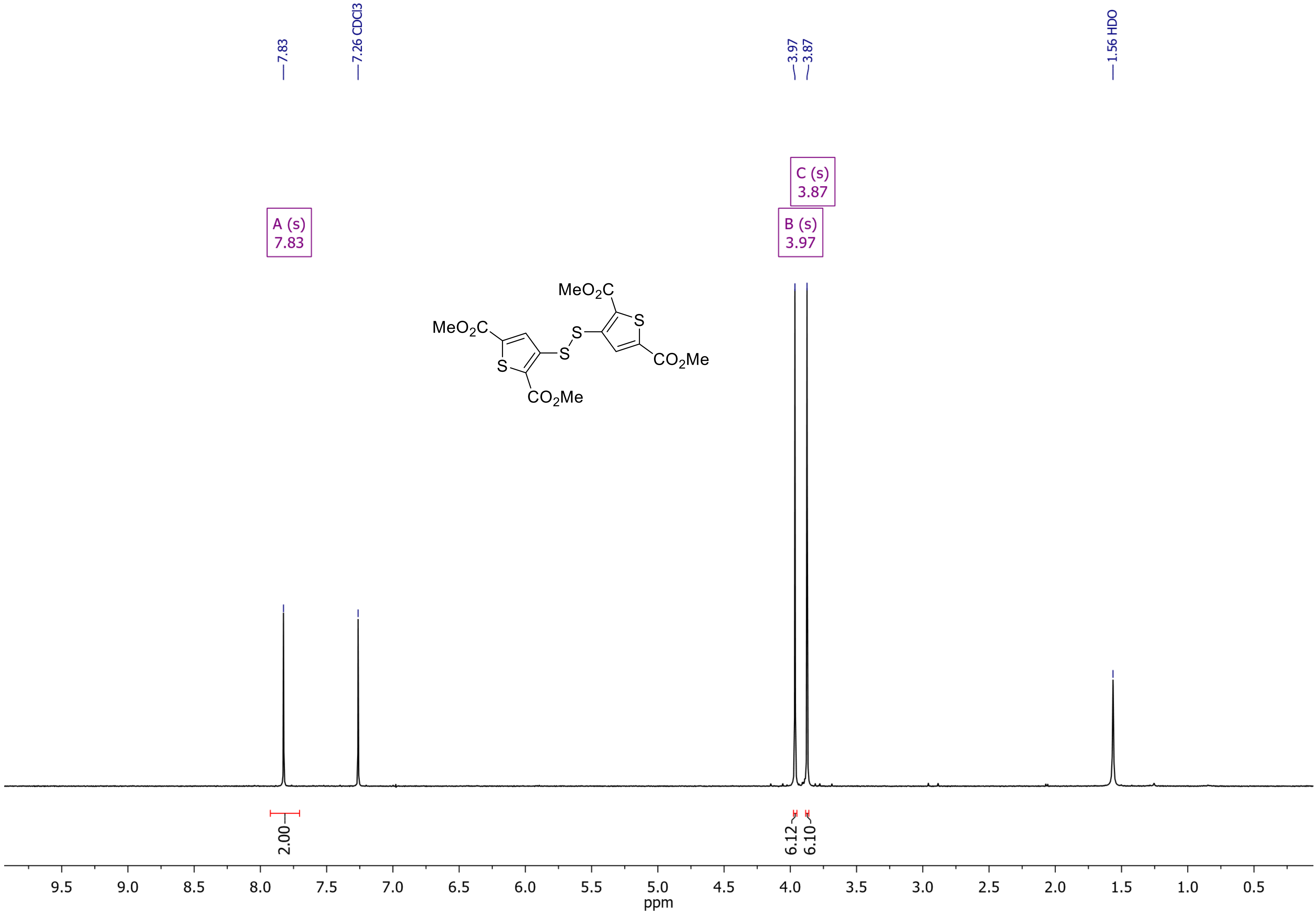

Tetramethyl 3,3'-disulfanediylbis(thiophene-2,5-dicarboxylate) (**3**)
^{13}C NMR (126 MHz, $CDCl_3$)

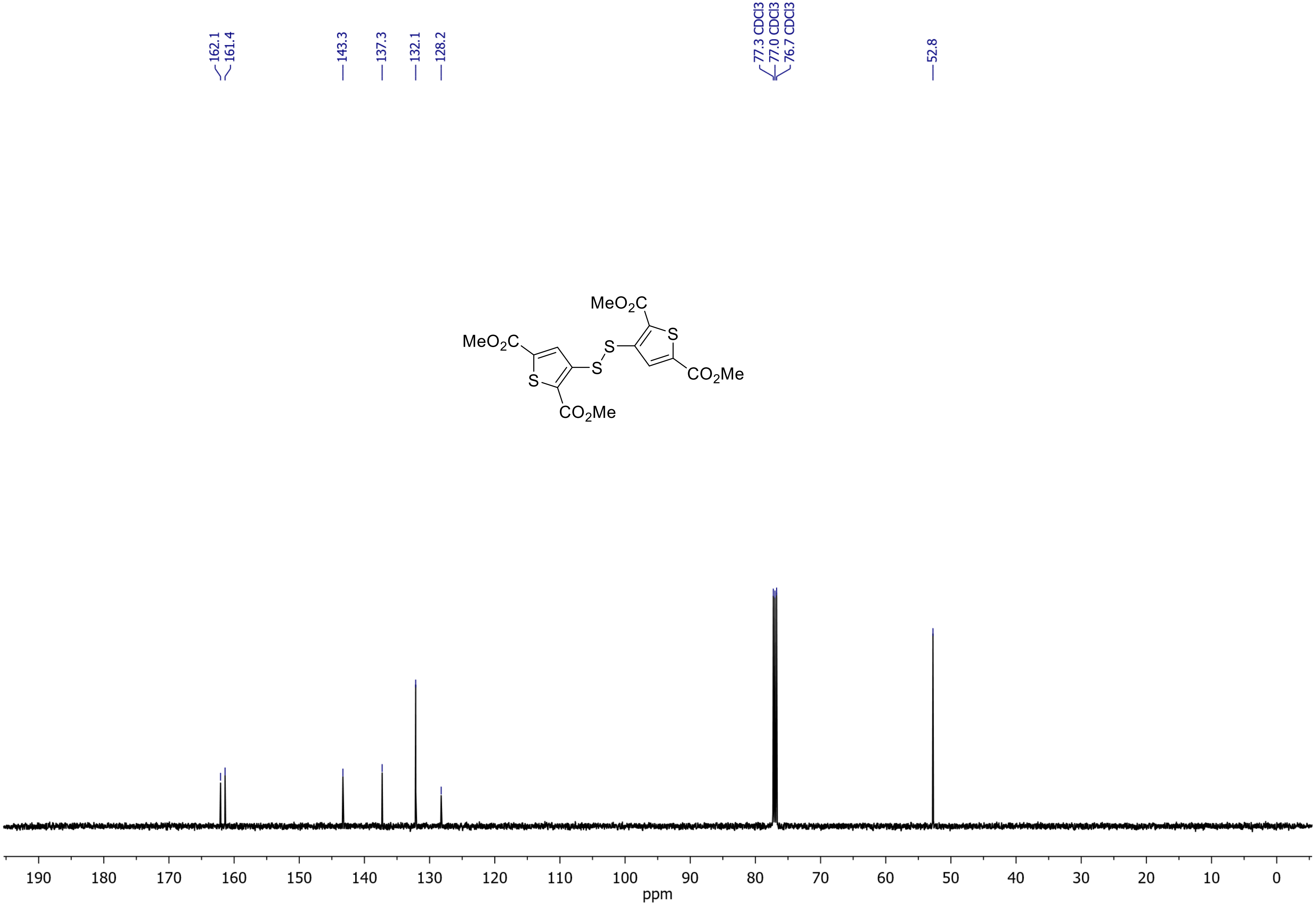

A mixture of compounds **2** and **3** from the reaction of ester **1** with potassium thioacetate

^{1}H NMR (400 MHz, $CDCl_3$)

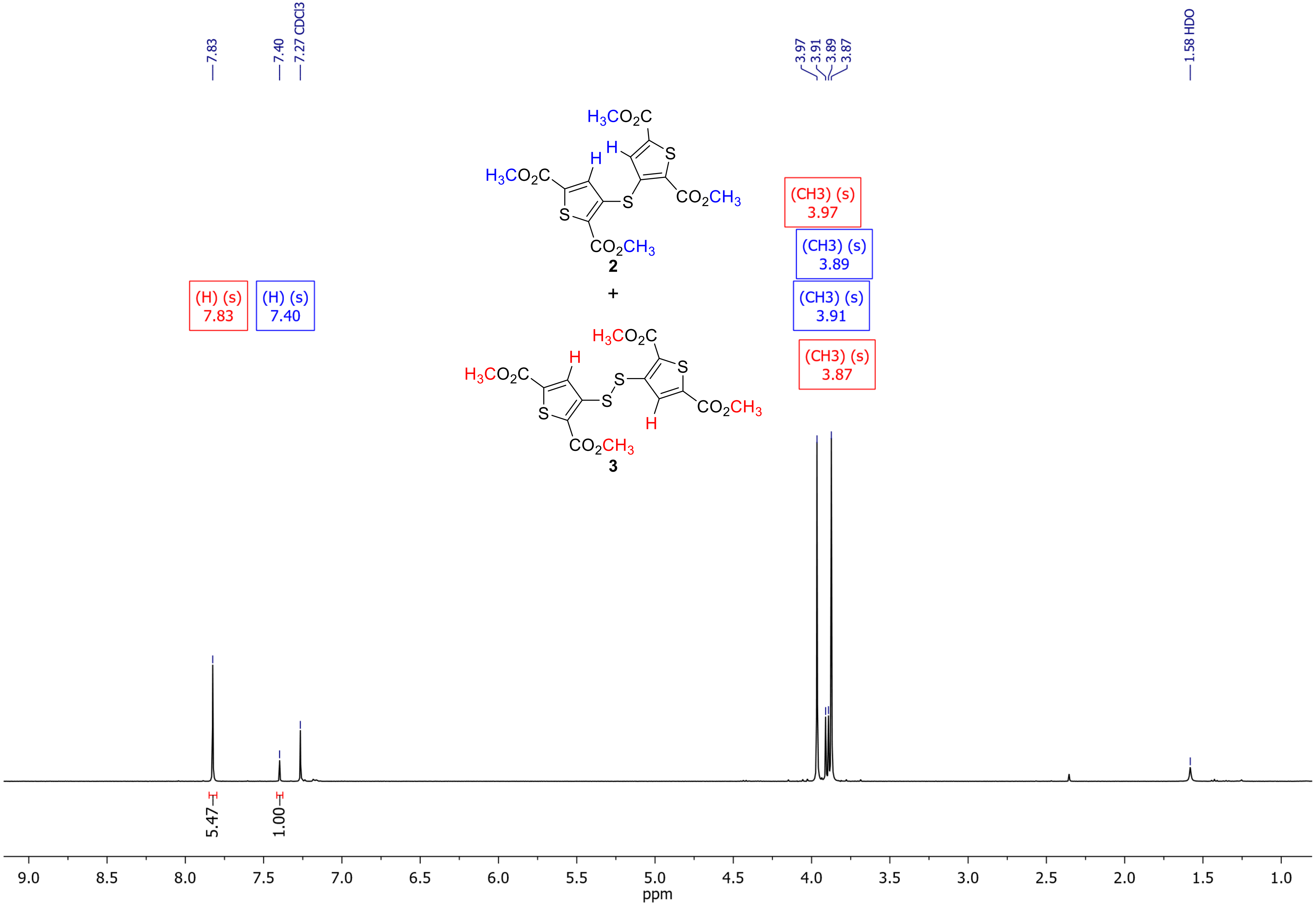

A mixture of compounds **2** and **3** from the reaction of ester **1** with potassium xanthate

^{1}H NMR (500 MHz, $CDCl_3$)

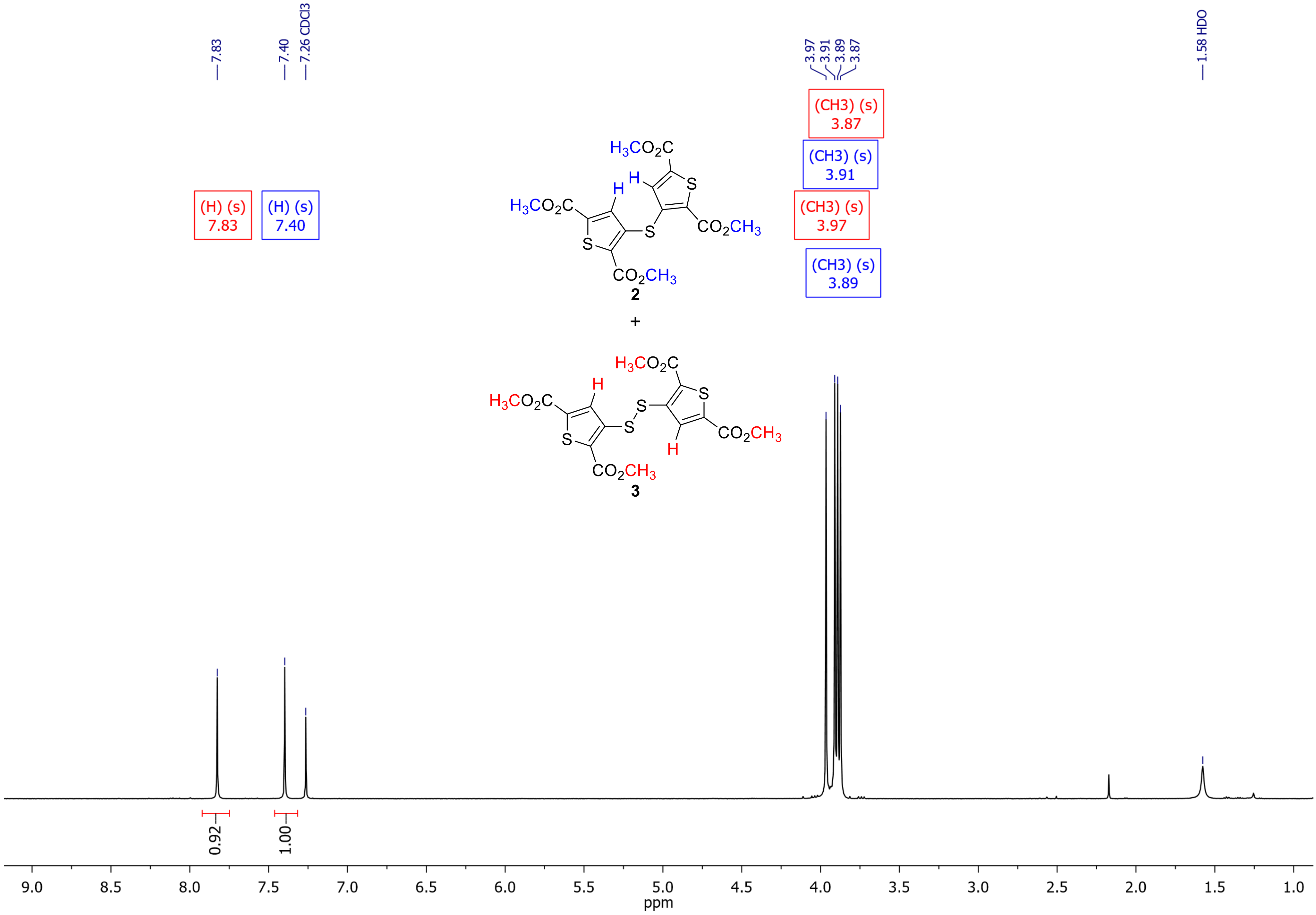

A mixture of compounds **2** and **3** from the reaction of ester **1** with sodium diethyldithiocarbamate
^{1}H NMR (400 MHz, $CDCl_3$)

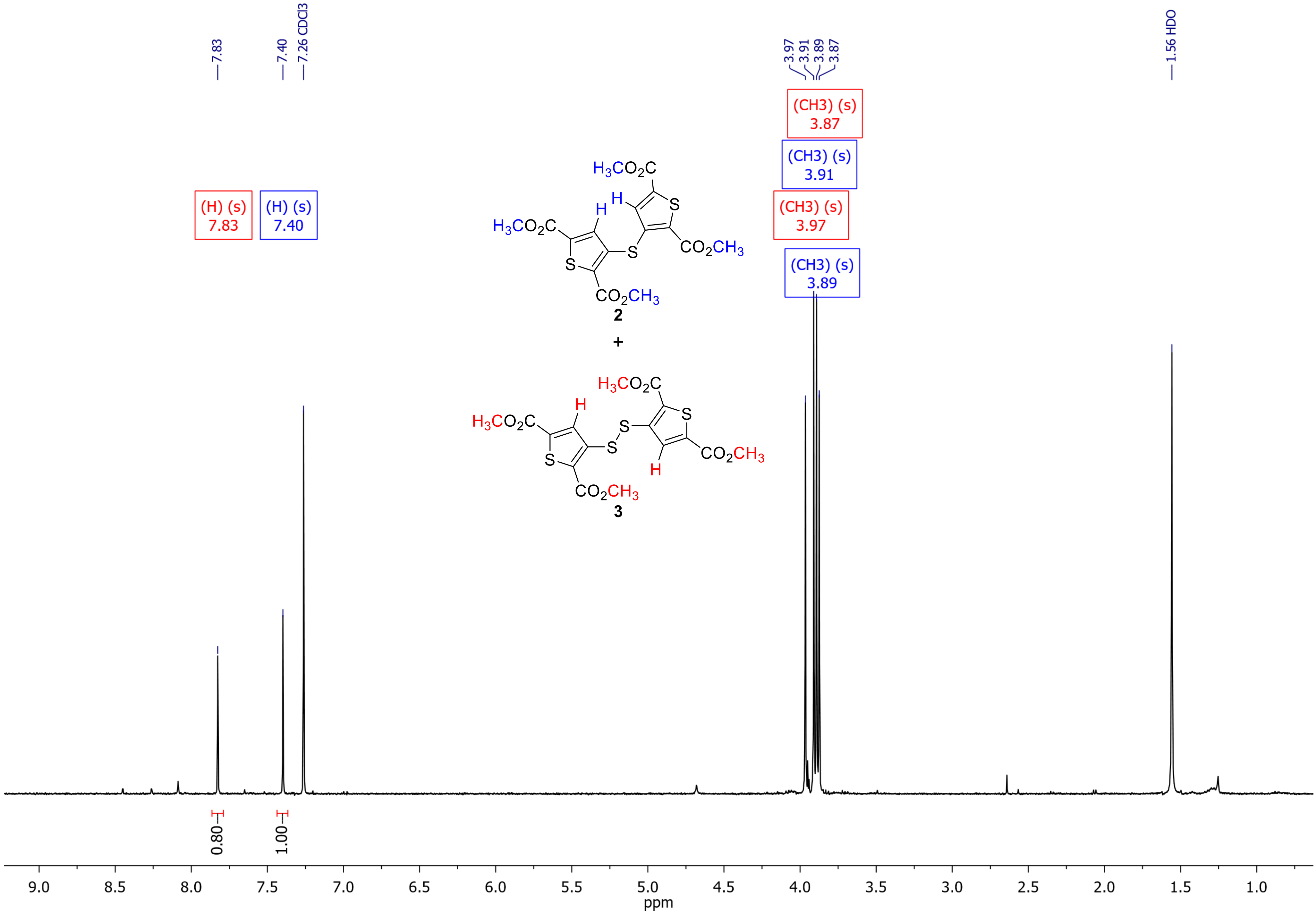

A mixture of compounds **2** and **3** from the reaction of ester **1** with thioacetamide and K_2CO_3

^{1}H NMR (400 MHz, $CDCl_3$)

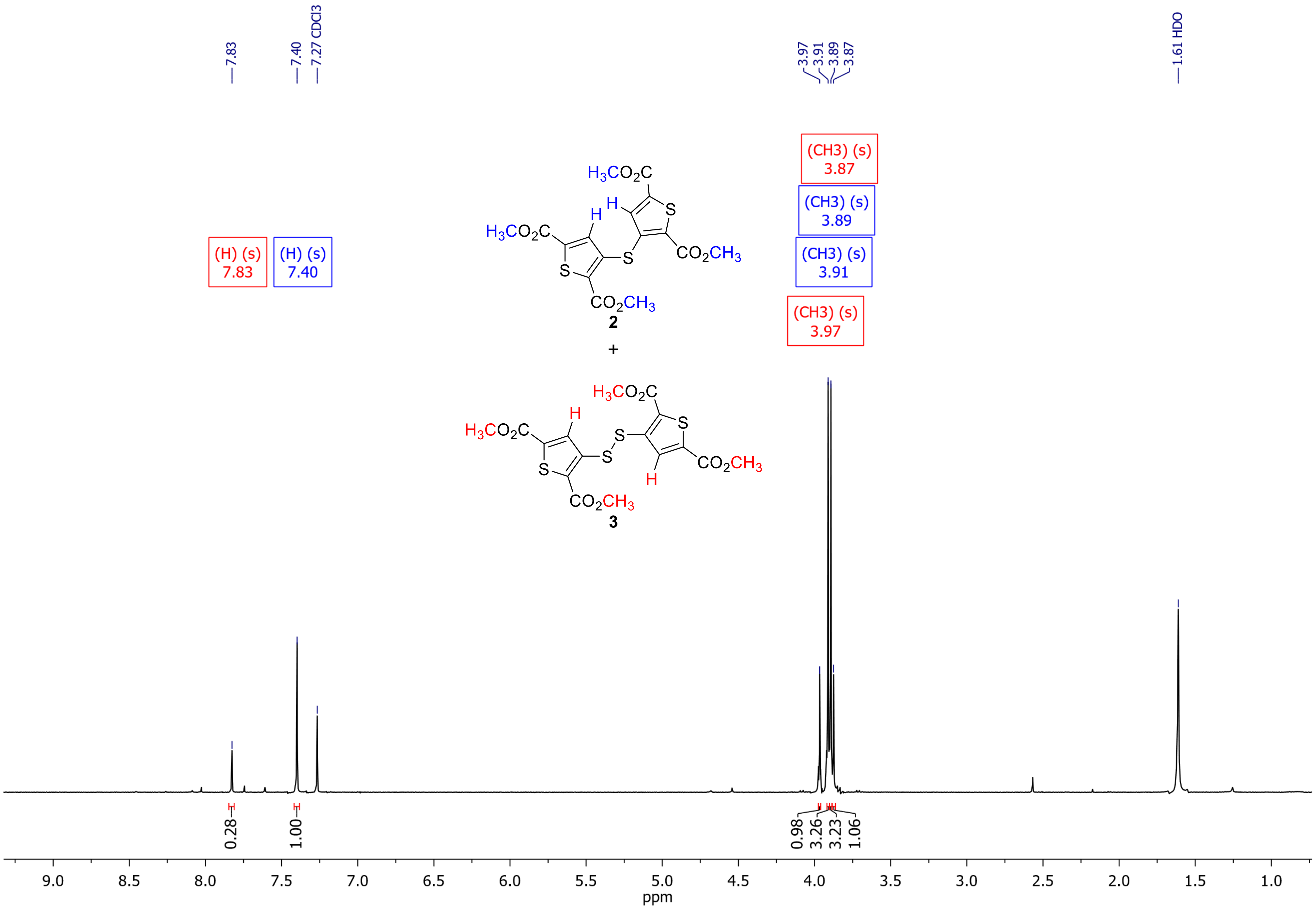

A mixture of compounds **2** and **3** from the reaction of ester **1** with Na_2S_2

^1H NMR (400 MHz, $CDCl_3$)

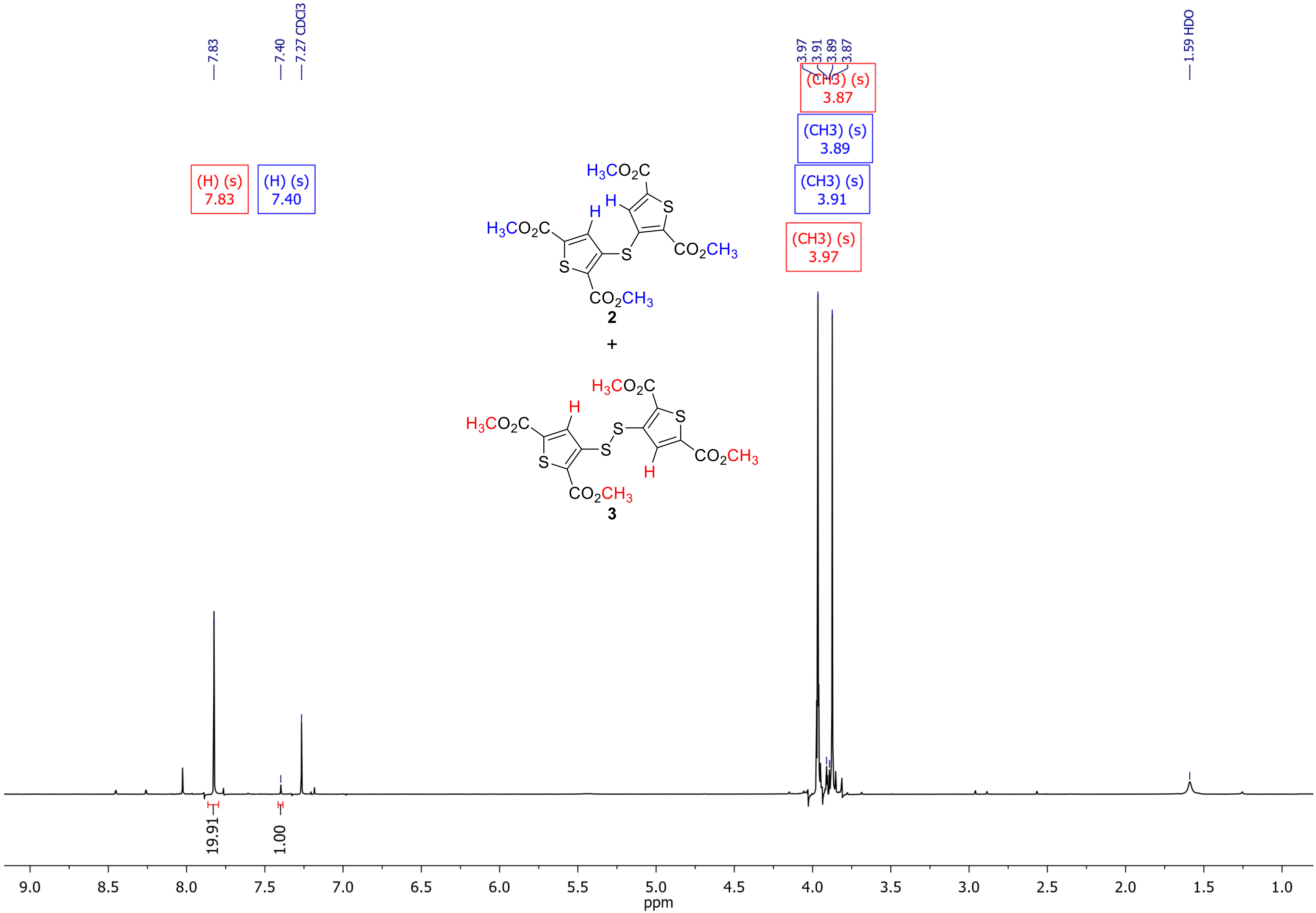

Dimethyl 3-[(4-cyanobenzyl)thio]thiophene-2,5-dicarboxylate (**4a**)

^{1}H NMR (400 MHz, $CDCl_3$)

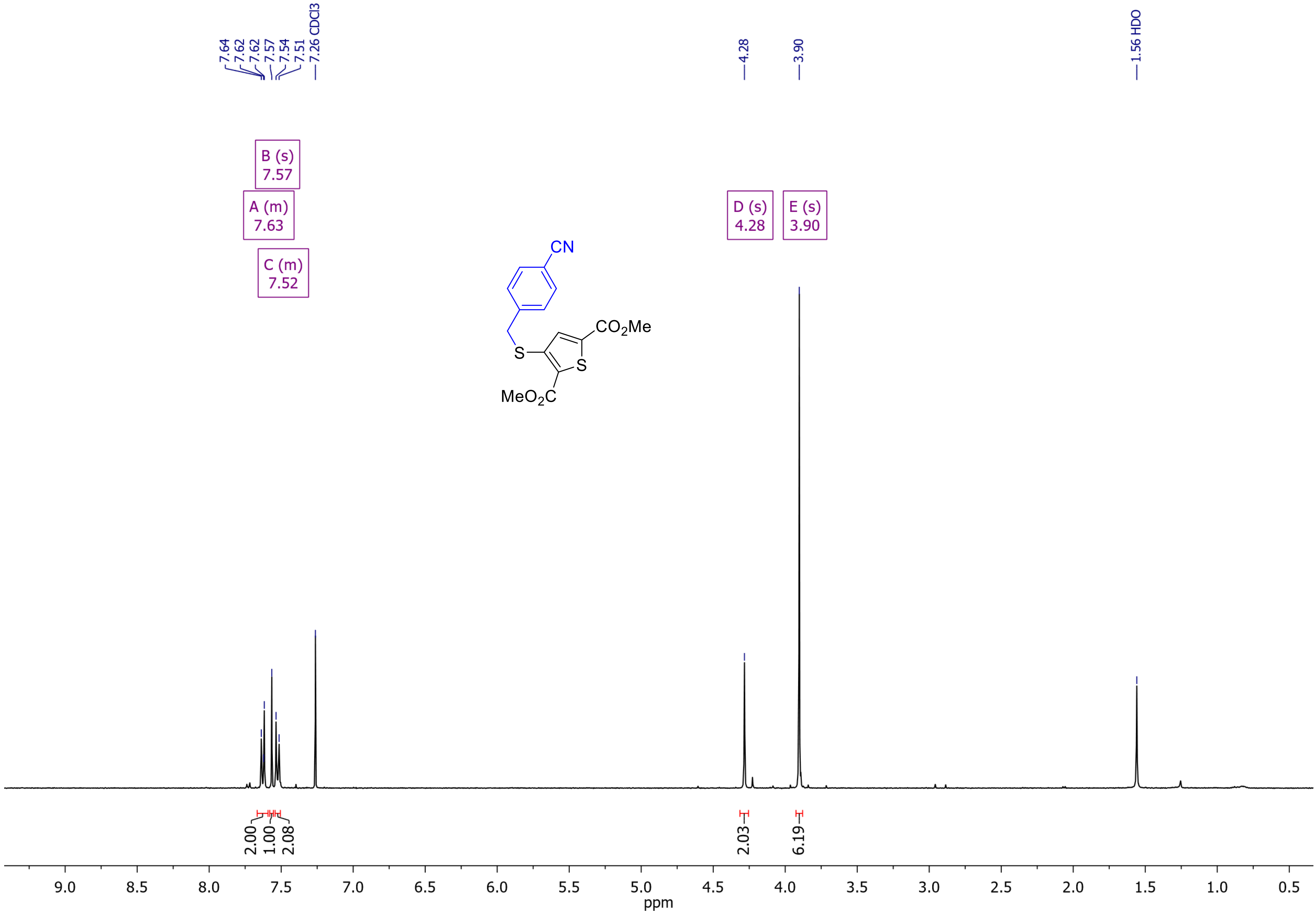

Dimethyl 3-[(4-cyanobenzyl)thio]thiophene-2,5-dicarboxylate (**4a**)

^{13}C NMR (126 MHz, $CDCl_3$)

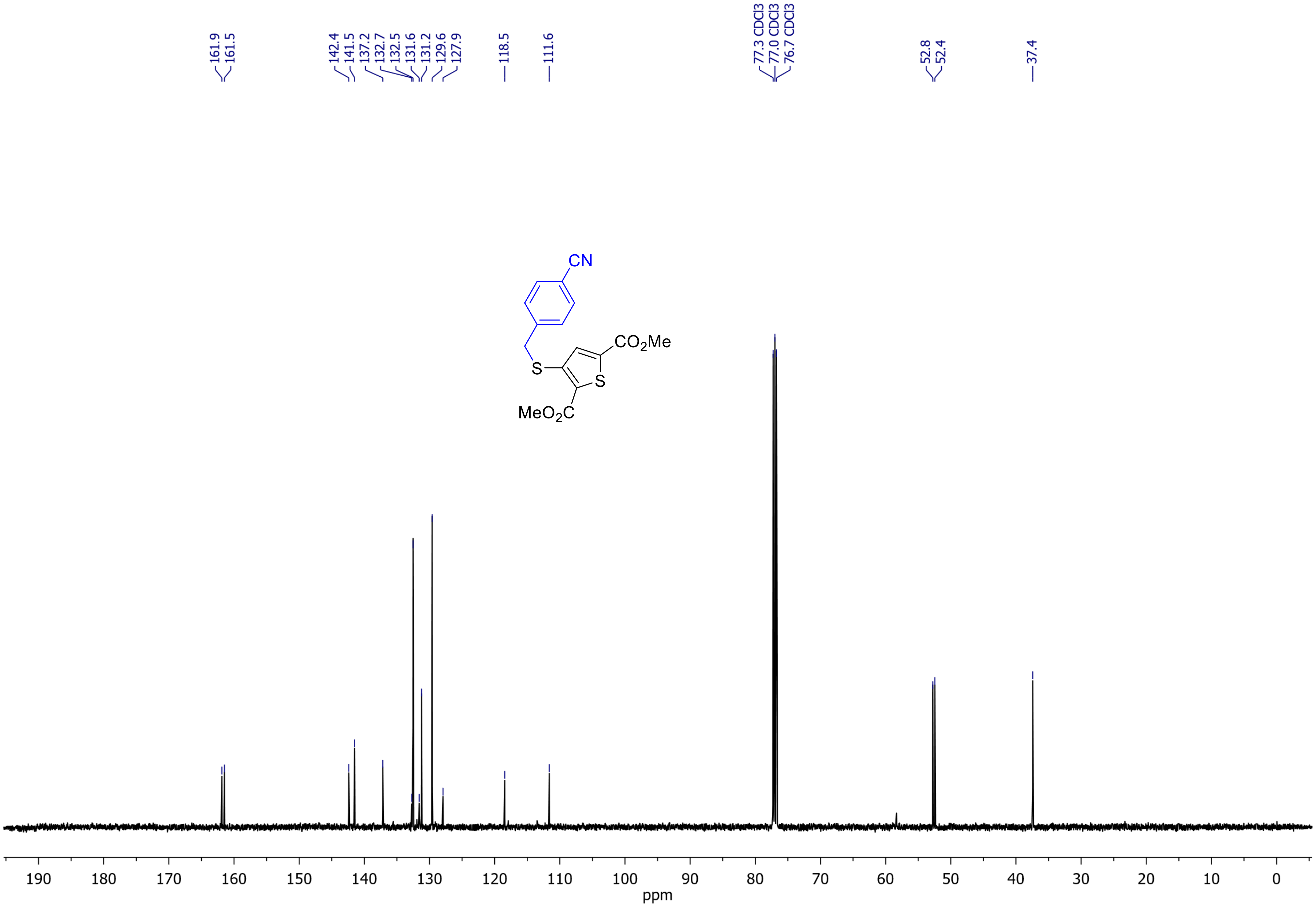

Dimethyl 3-{[4-(methoxycarbonyl)benzyl]thio}thiophene-2,5-dicarboxylate (**4b**)

^{1}H NMR (400 MHz, $CDCl_3$)

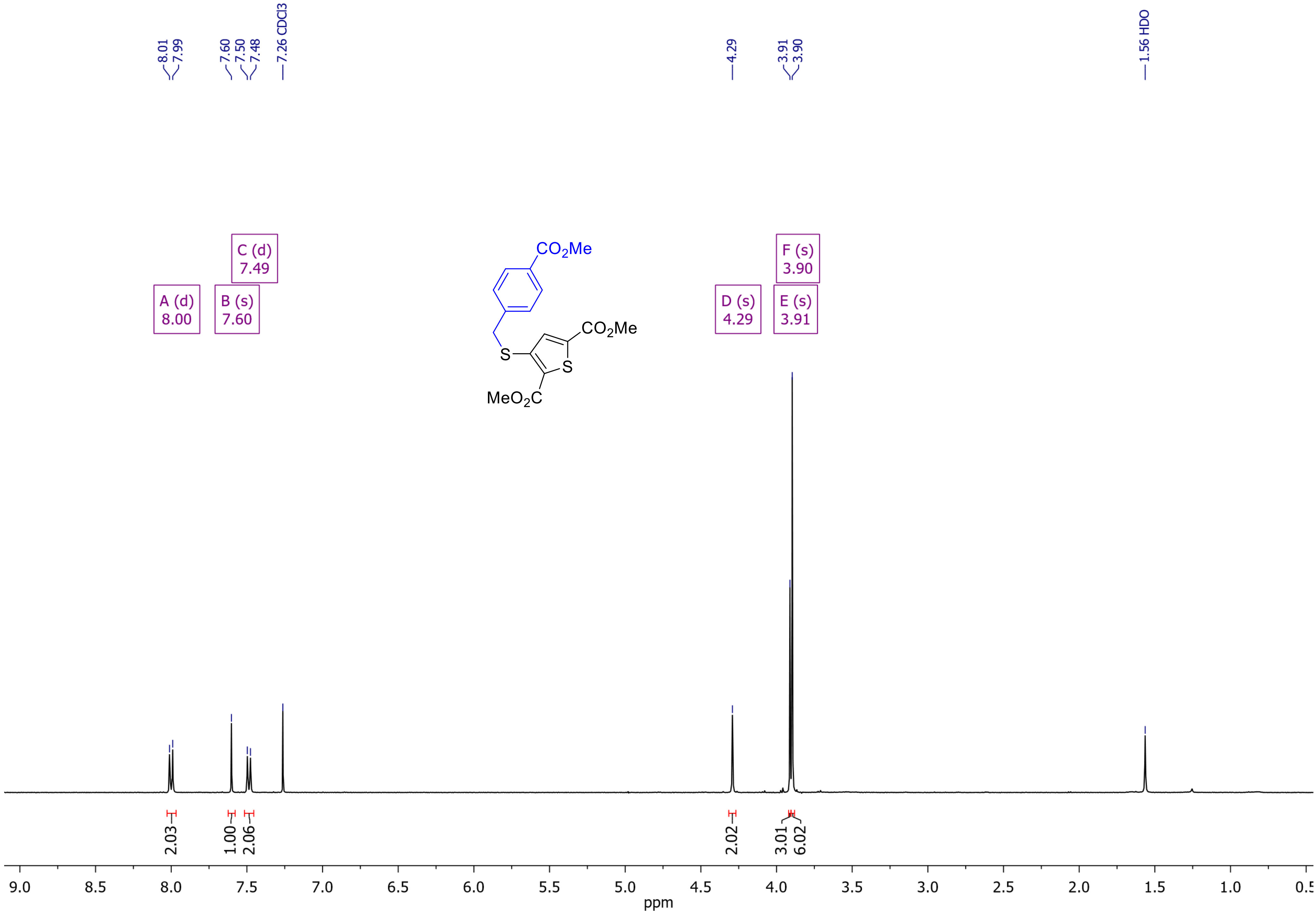

Dimethyl 3-{[4-(methoxycarbonyl)benzyl]thio}thiophene-2,5-dicarboxylate (**4b**)
^{13}C NMR (126 MHz, $CDCl_3$)

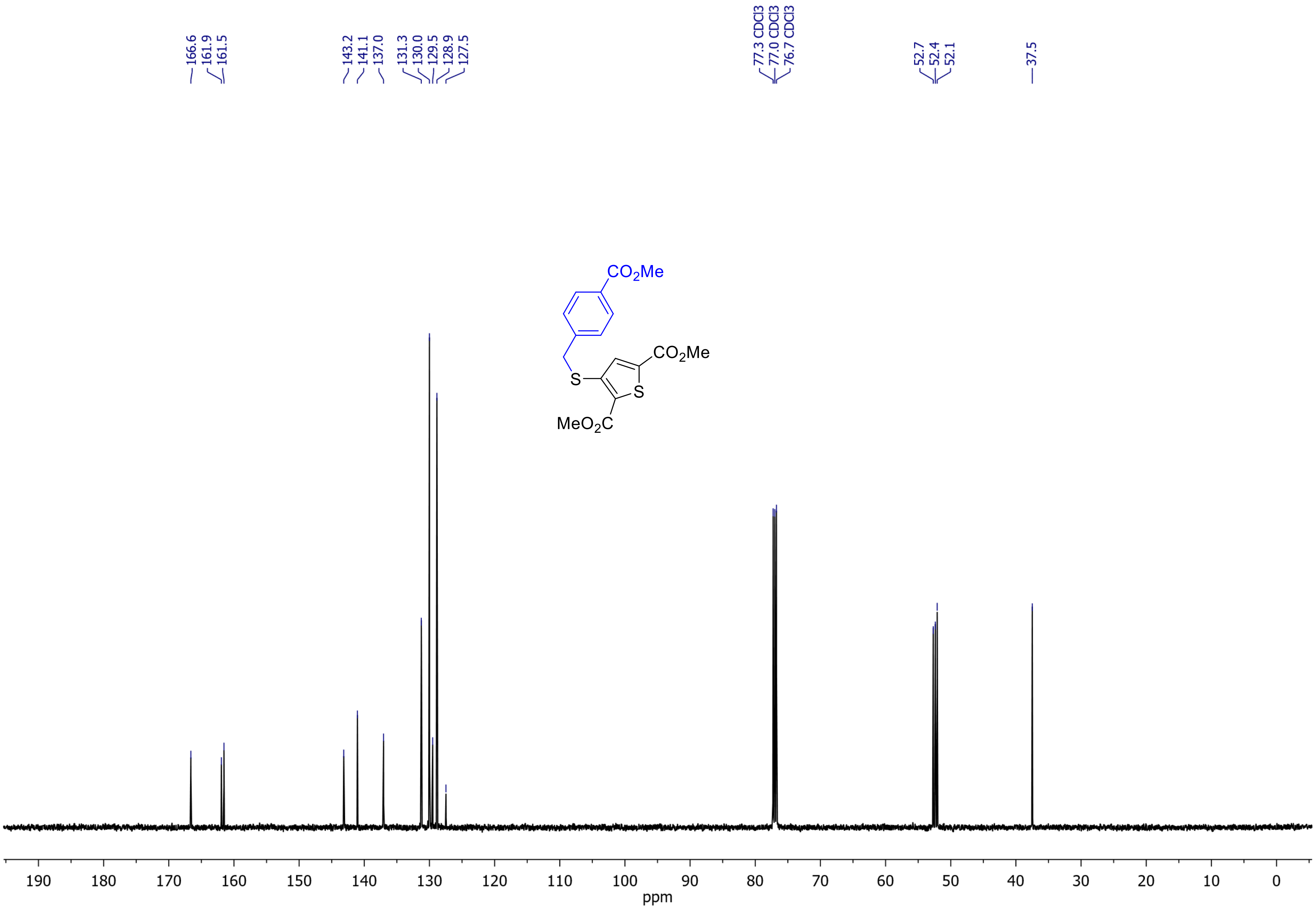

Dimethyl 3-(benzylthio)thiophene-2,5-dicarboxylate (**4c**)

^{1}H NMR (400 MHz, $CDCl_3$)

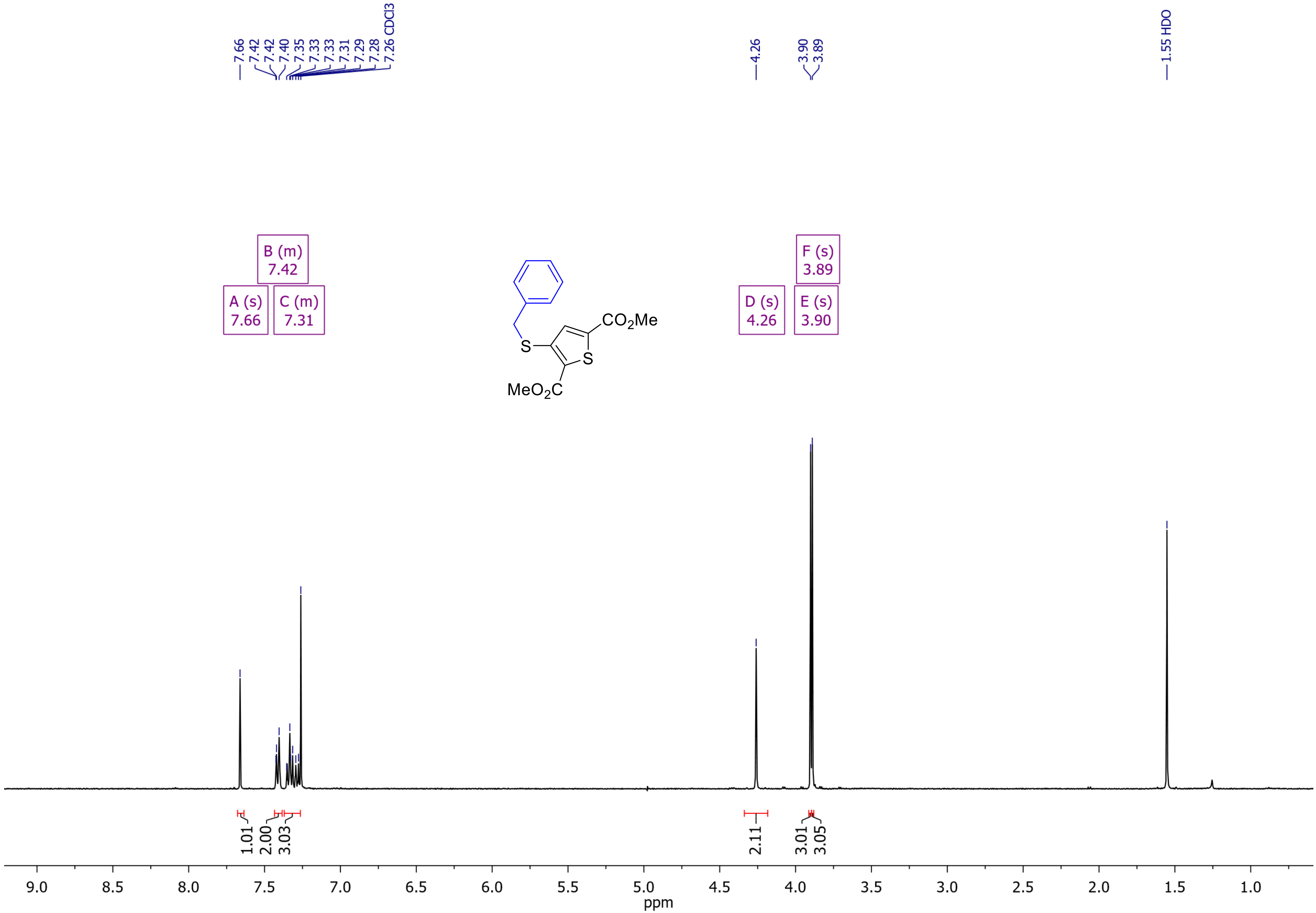

Dimethyl 3-(benzylthio)thiophene-2,5-dicarboxylate (**4c**)

^{13}H NMR (126 MHz, $CDCl_3$)

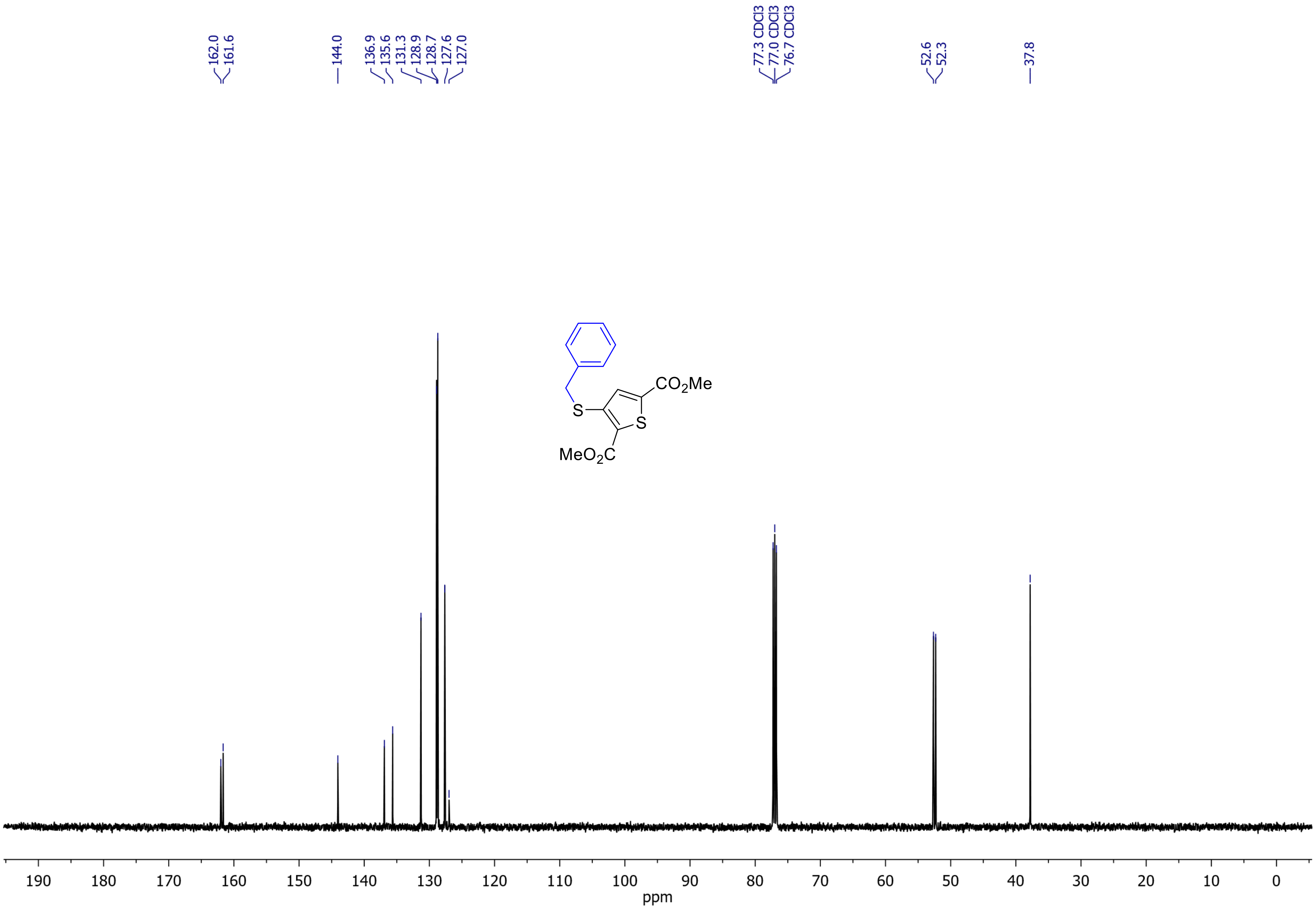

Dimethyl 3-[(4-fluorobenzyl)thio]thiophene-2,5-dicarboxylate (**4d**)

^{1}H NMR (500 MHz, $CDCl_3$)

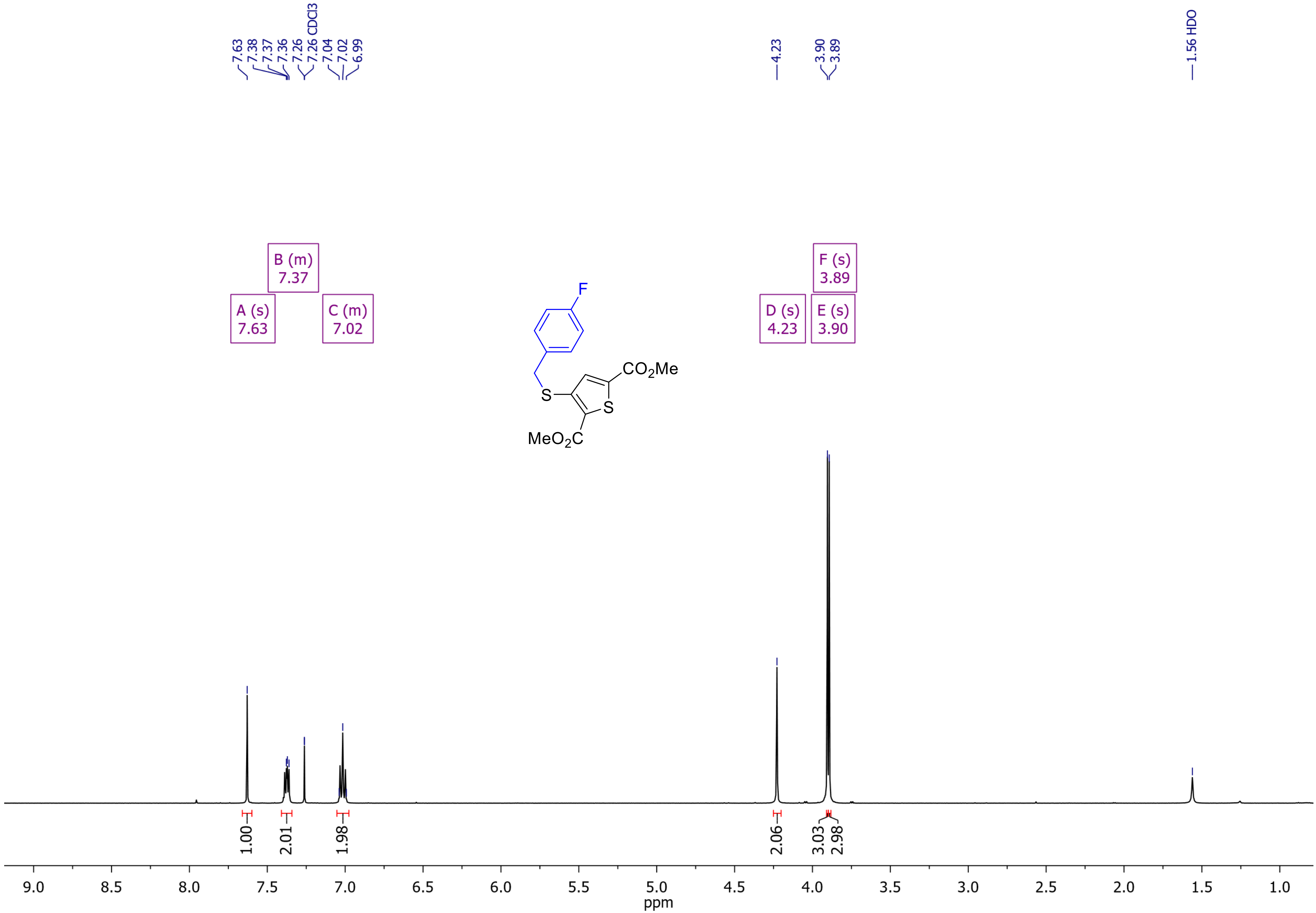

Dimethyl 3-[(4-fluorobenzyl)thio]thiophene-2,5-dicarboxylate (**4d**)

^{19}F NMR (471 MHz, $CDCl_3$)

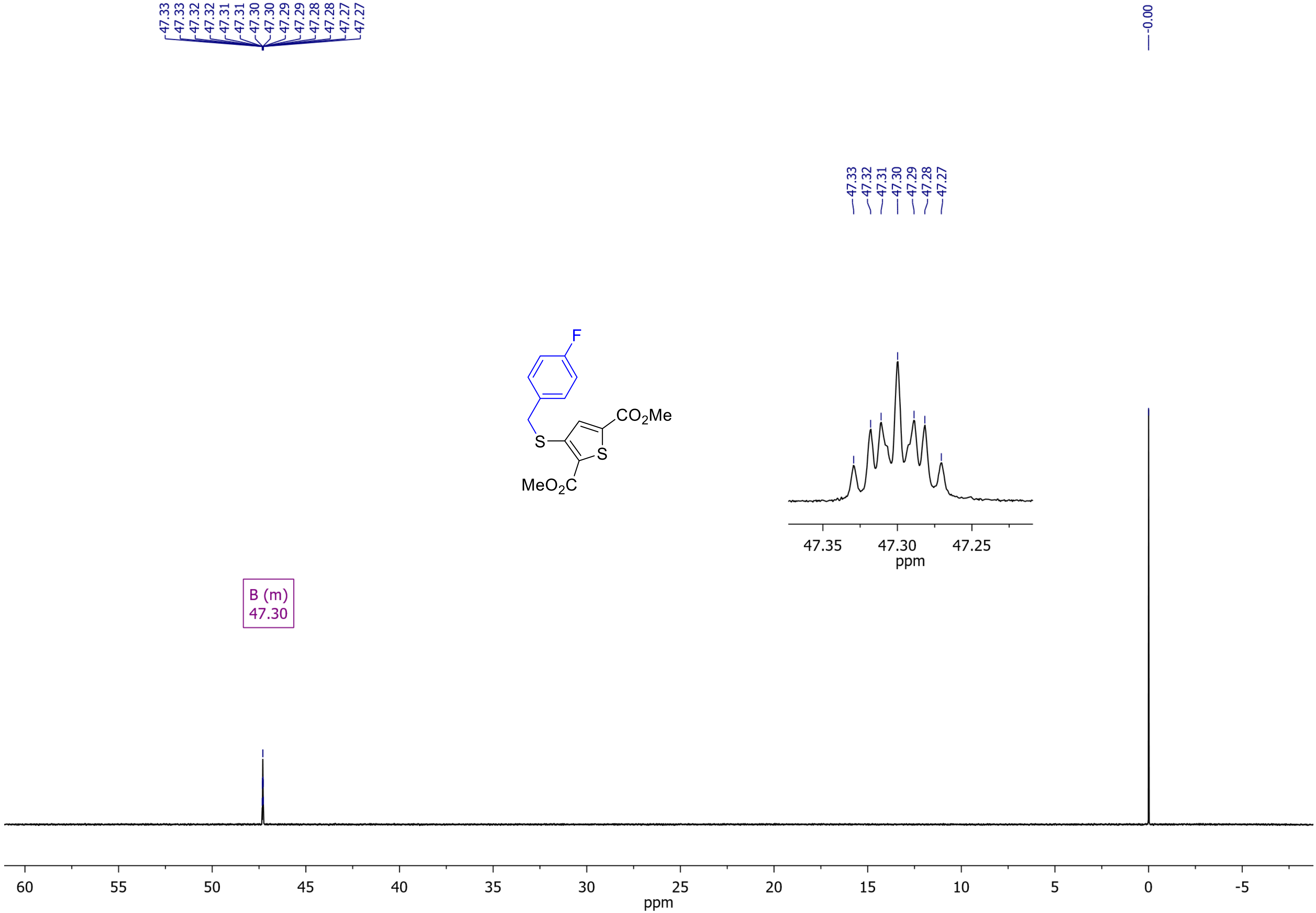

Dimethyl 3-[(4-fluorobenzyl)thio]thiophene-2,5-dicarboxylate (**4d**)

^{13}C NMR (126 MHz, $CDCl_3$)

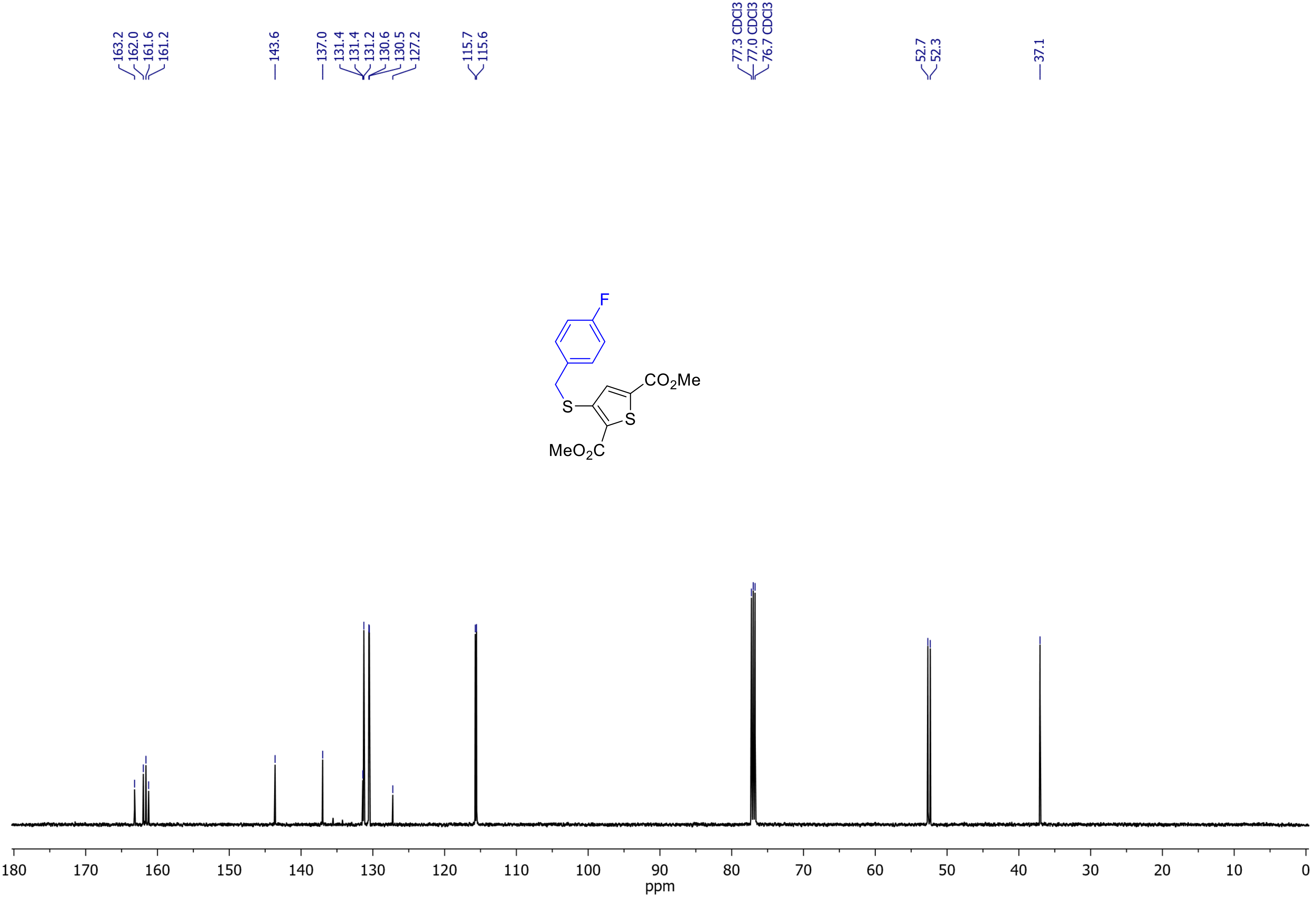

Dimethyl 3-[(3,4-difluorobenzyl)thio]thiophene-2,5-dicarboxylate (**4e**)
^{1}H NMR (400 MHz, DMSO-d_6)

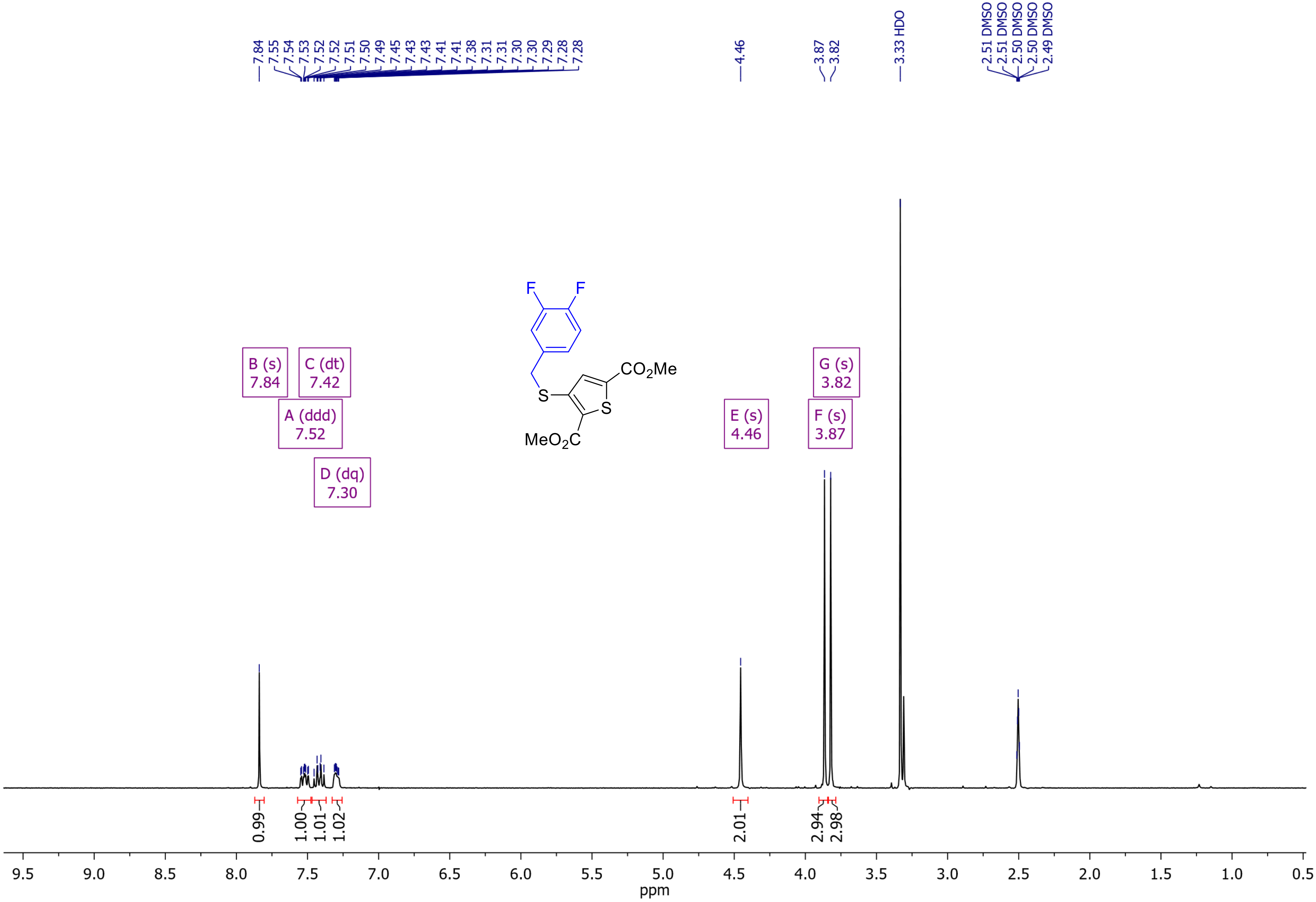

Dimethyl 3-[(3,4-difluorobenzyl)thio]thiophene-2,5-dicarboxylate (**4e**)

^{19}F NMR (376 MHz, $CDCl_3$)

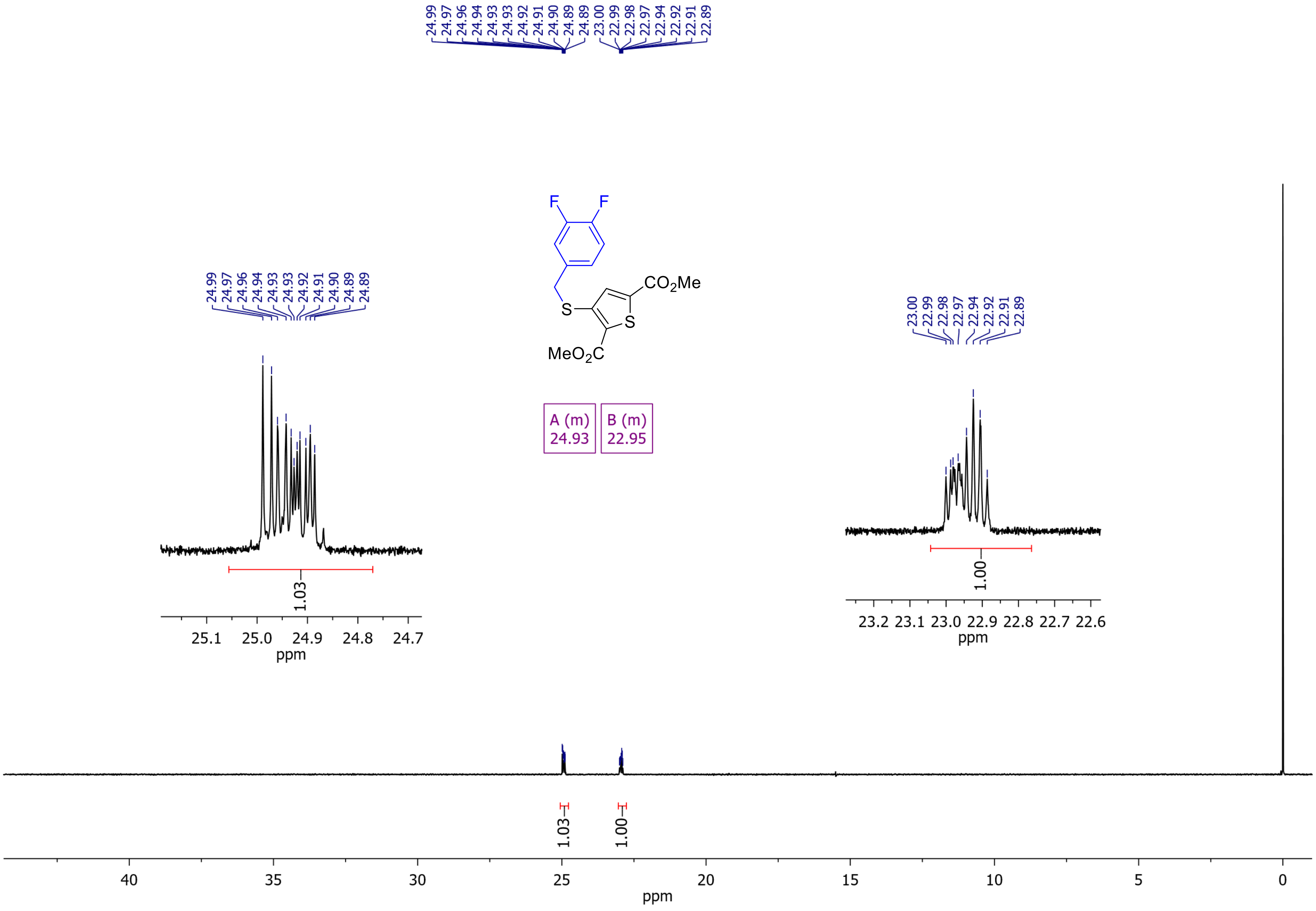

Dimethyl 3-[(3,4-difluorobenzyl)thio]thiophene-2,5-dicarboxylate (**4e**)

^{13}C NMR (126 MHz, $CDCl_3$)

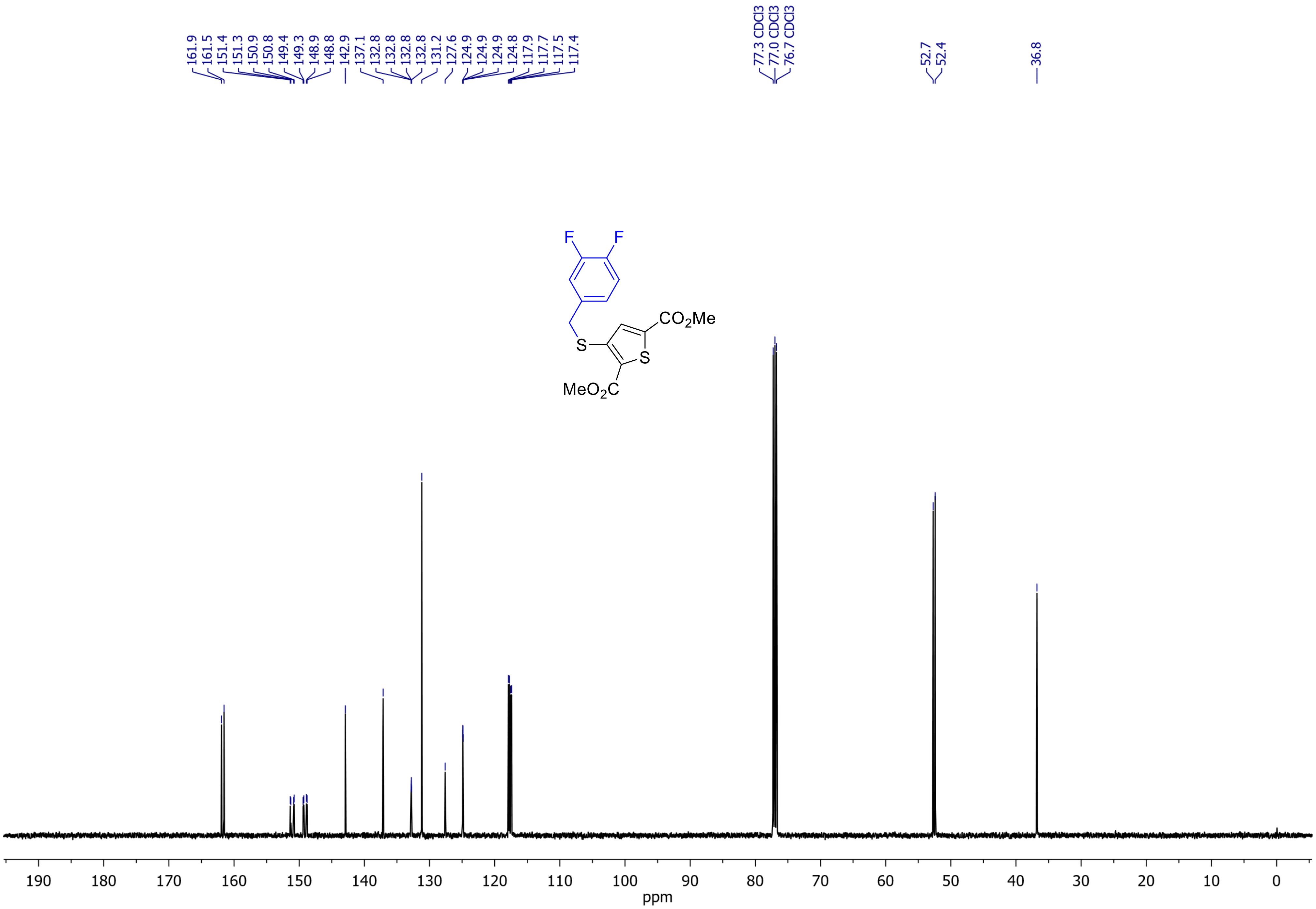

Dimethyl 3-{[4-(trifluoromethyl)benzyl]thio}thiophene-2,5-dicarboxylate (**4f**)

^{1}H NMR (400 MHz, $CDCl_3$)

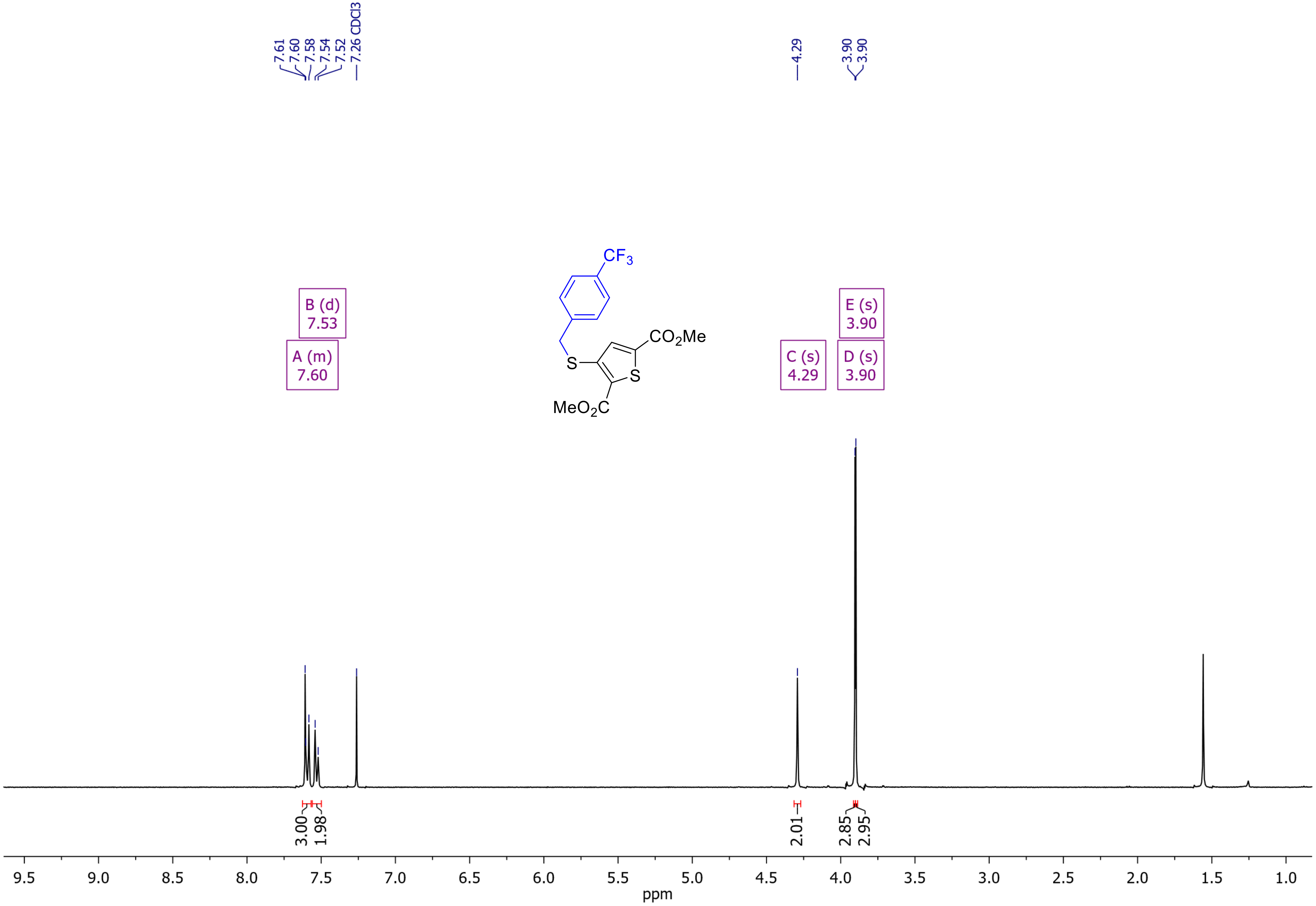

Dimethyl 3-{[4-(trifluoromethyl)benzyl]thio}thiophene-2,5-dicarboxylate (**4f**)

^{19}F NMR (376 MHz, $CDCl_3$)

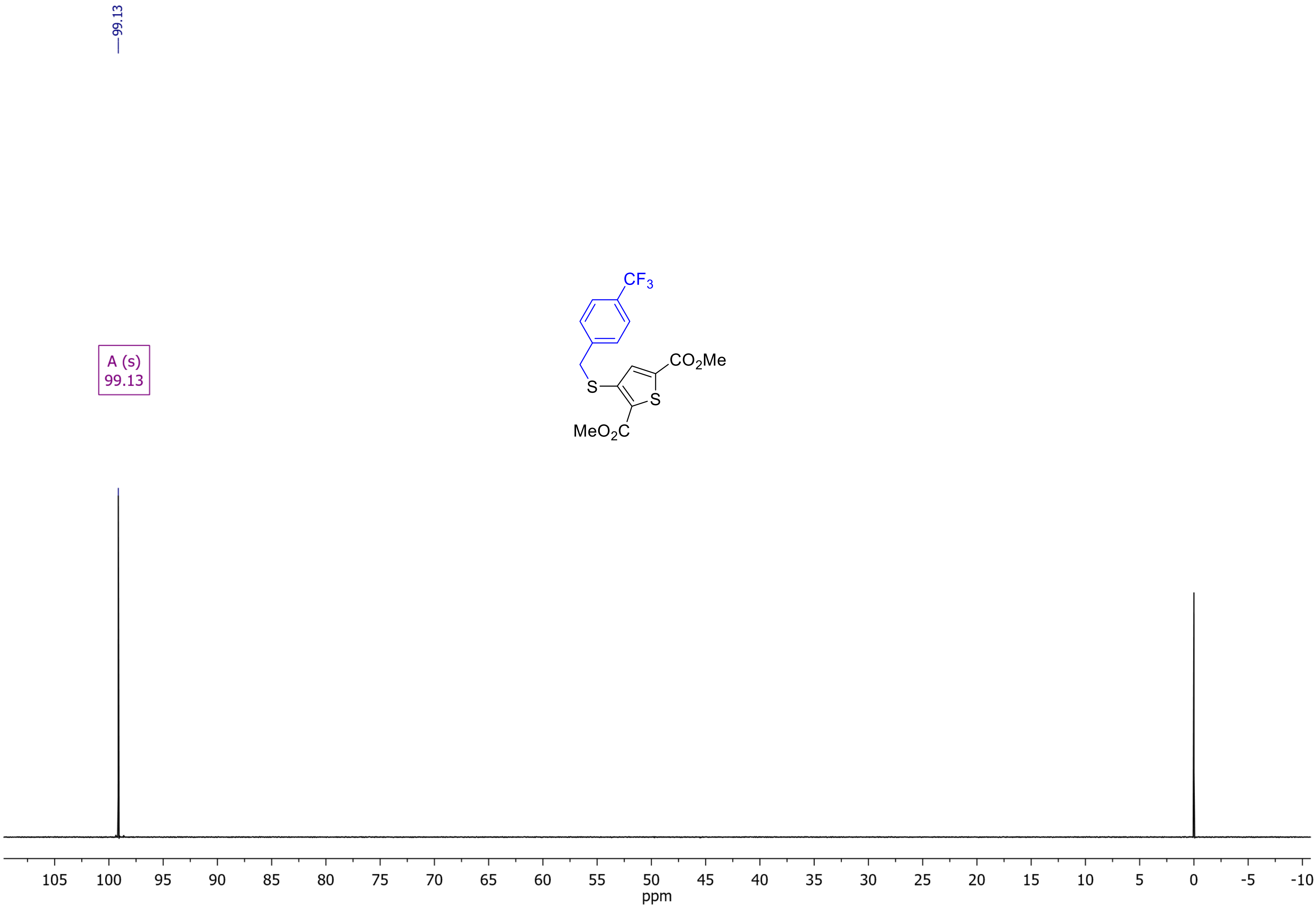

Dimethyl 3-{[4-(trifluoromethyl)benzyl]thio}thiophene-2,5-dicarboxylate (**4f**)
^{13}C NMR (126 MHz, CDCl$_3$)

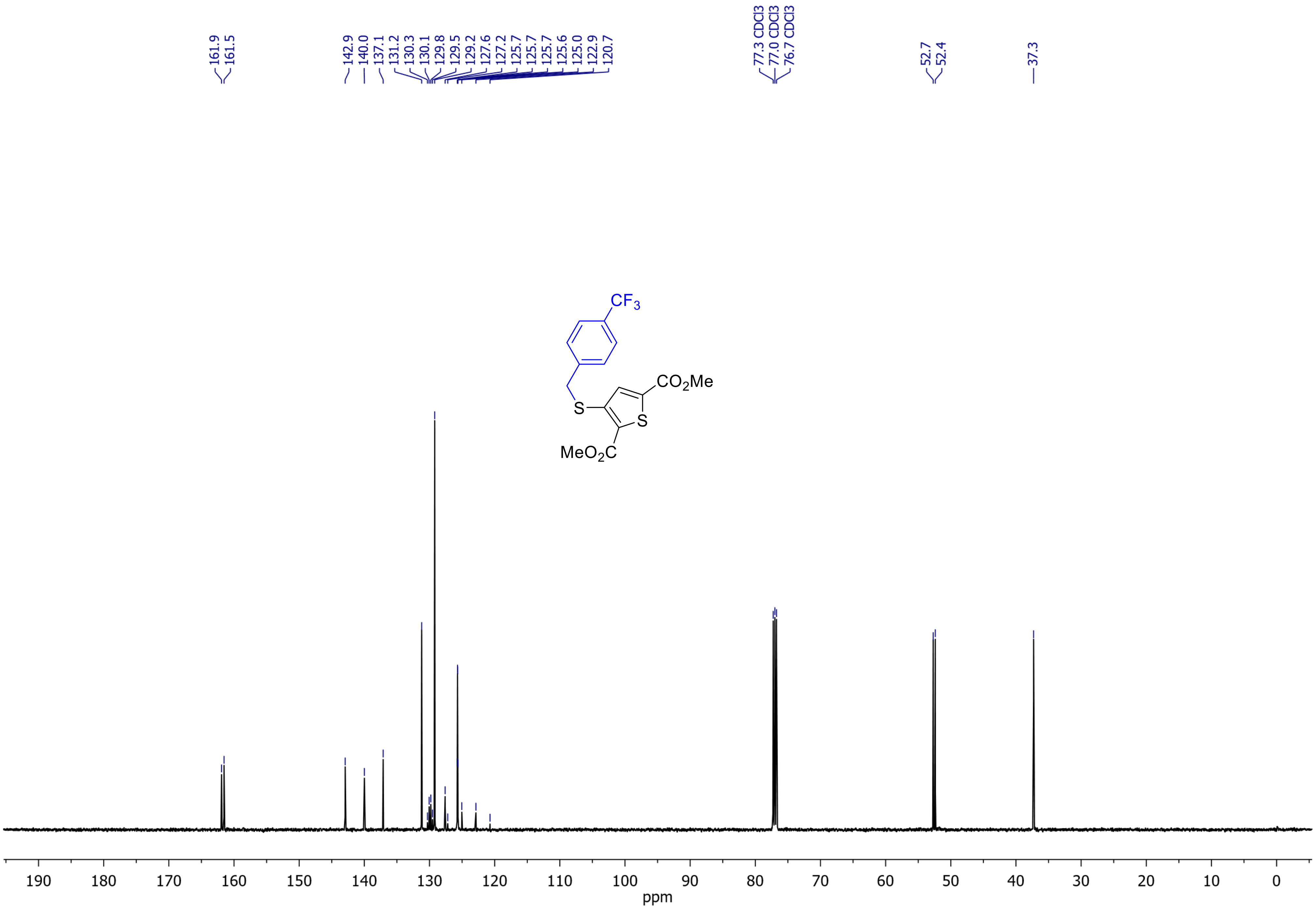

Dimethyl 3-[(benzo[*d*][1,3]dioxol-5-ylmethyl)thio]thiophene-2,5-dicarboxylate (**4g**)

^{1}H NMR (500 MHz, $CDCl_3$)

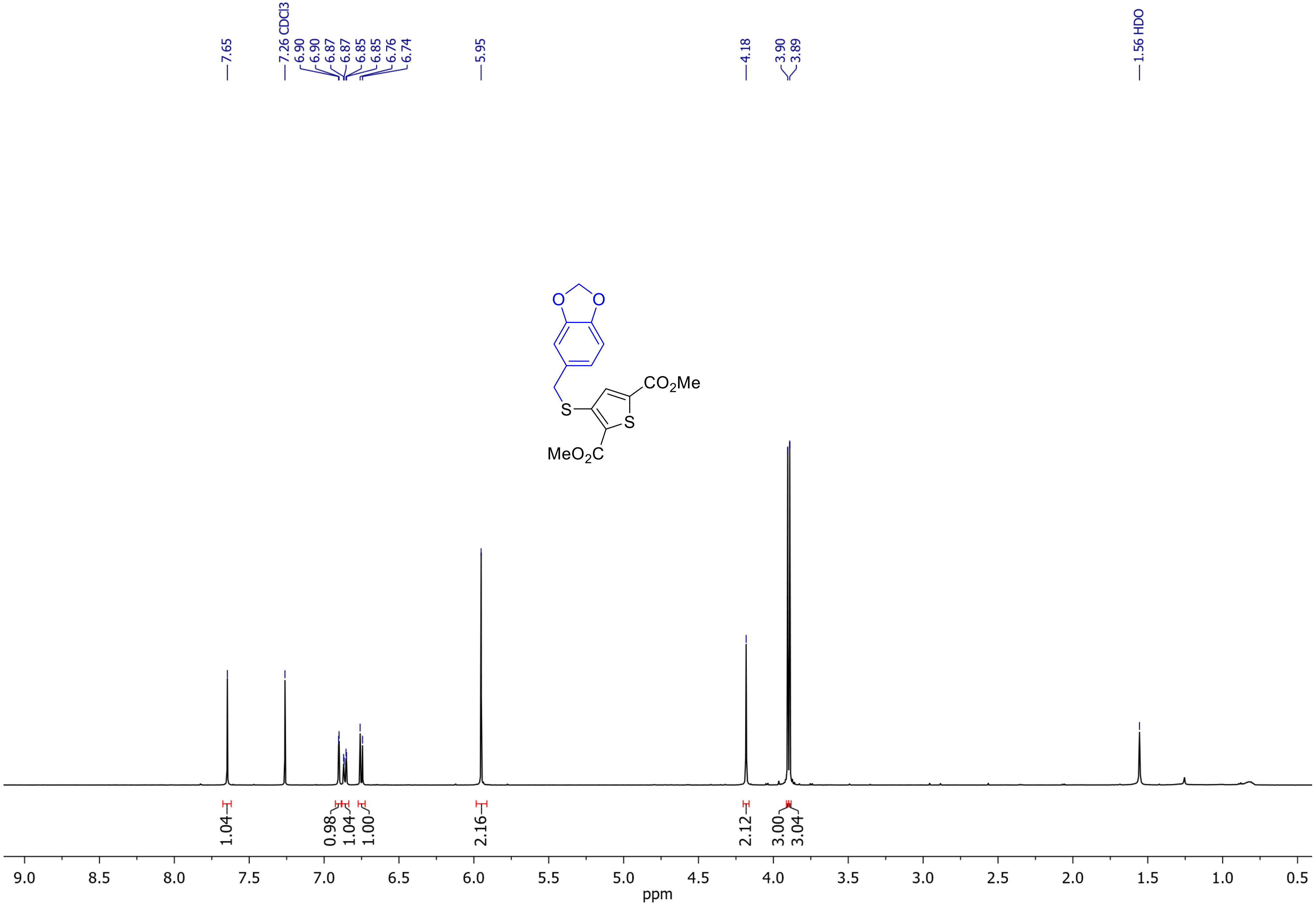

Dimethyl 3-[(benzo[*d*][1,3]dioxol-5-ylmethyl)thio]thiophene-2,5-dicarboxylate (**4g**)

^{13}C NMR (126 MHz, $CDCl_3$)

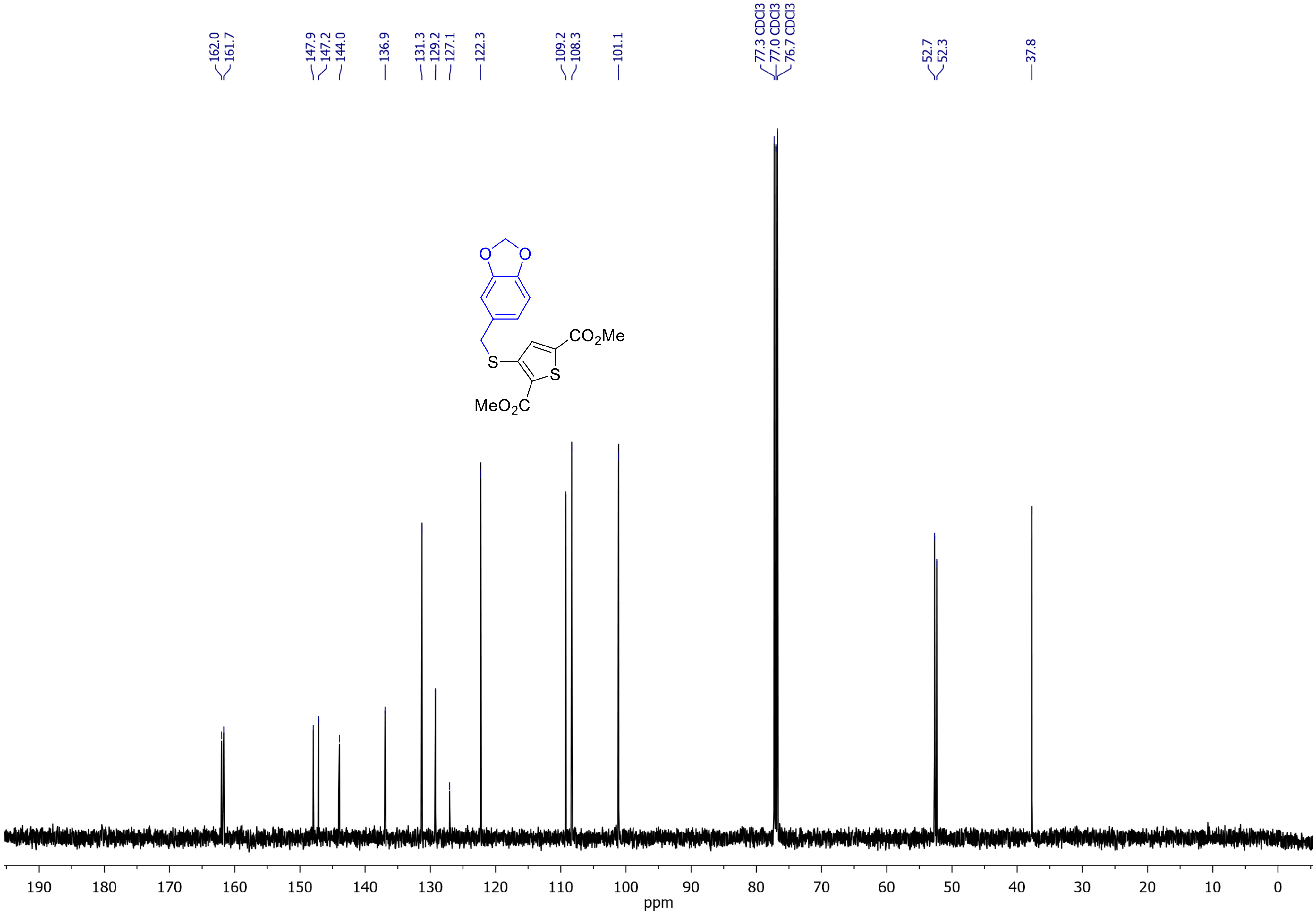

Dimethyl 3-[(thiophen-2-ylmethyl)thio]thiophene-2,5-dicarboxylate (**4h**)
^{1}H NMR (500 MHz, $CDCl_3$)

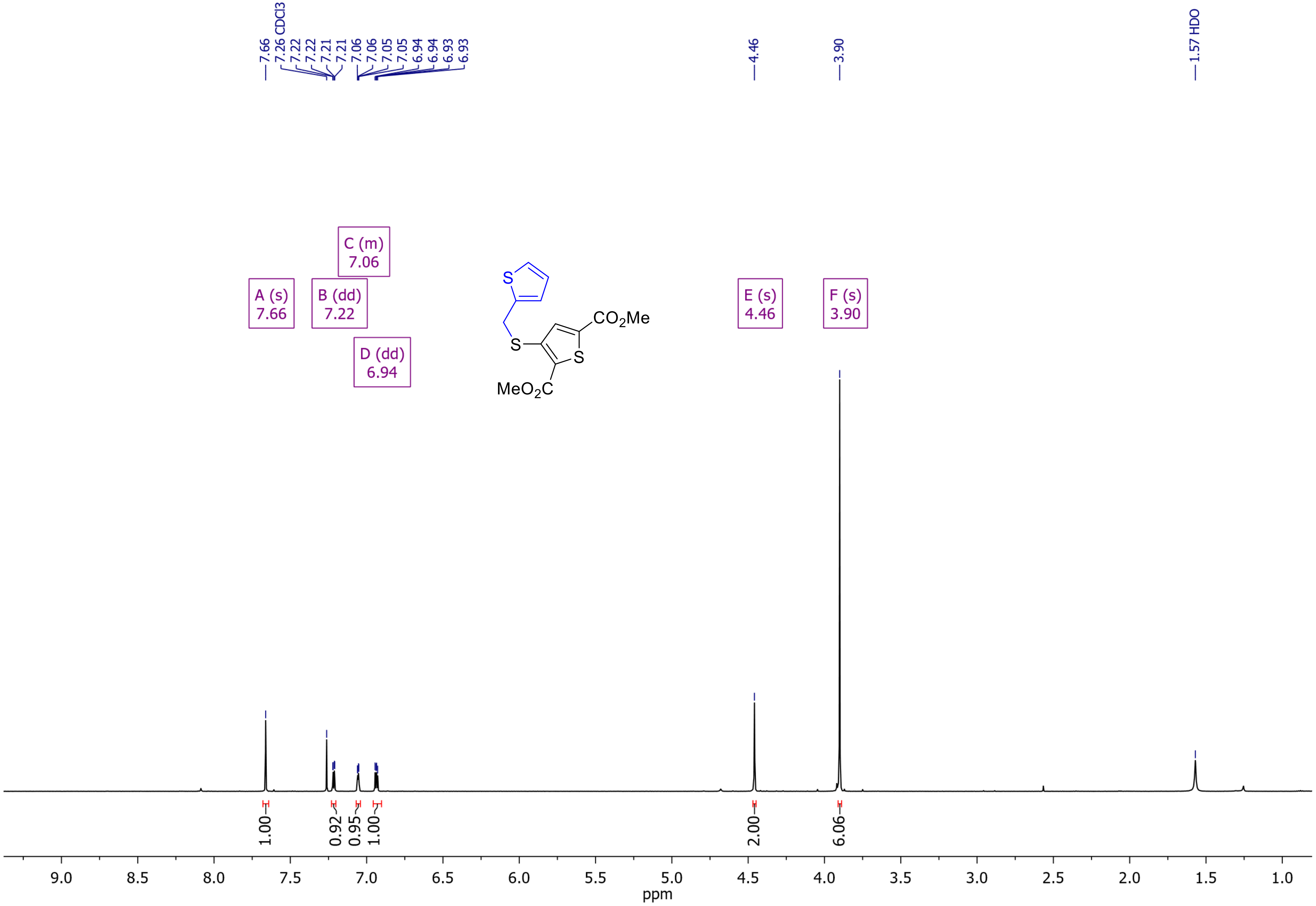

Dimethyl 3-[(thiophen-2-ylmethyl)thio]thiophene-2,5-dicarboxylate (**4h**)

^{13}C NMR (126 MHz, $CDCl_3$)

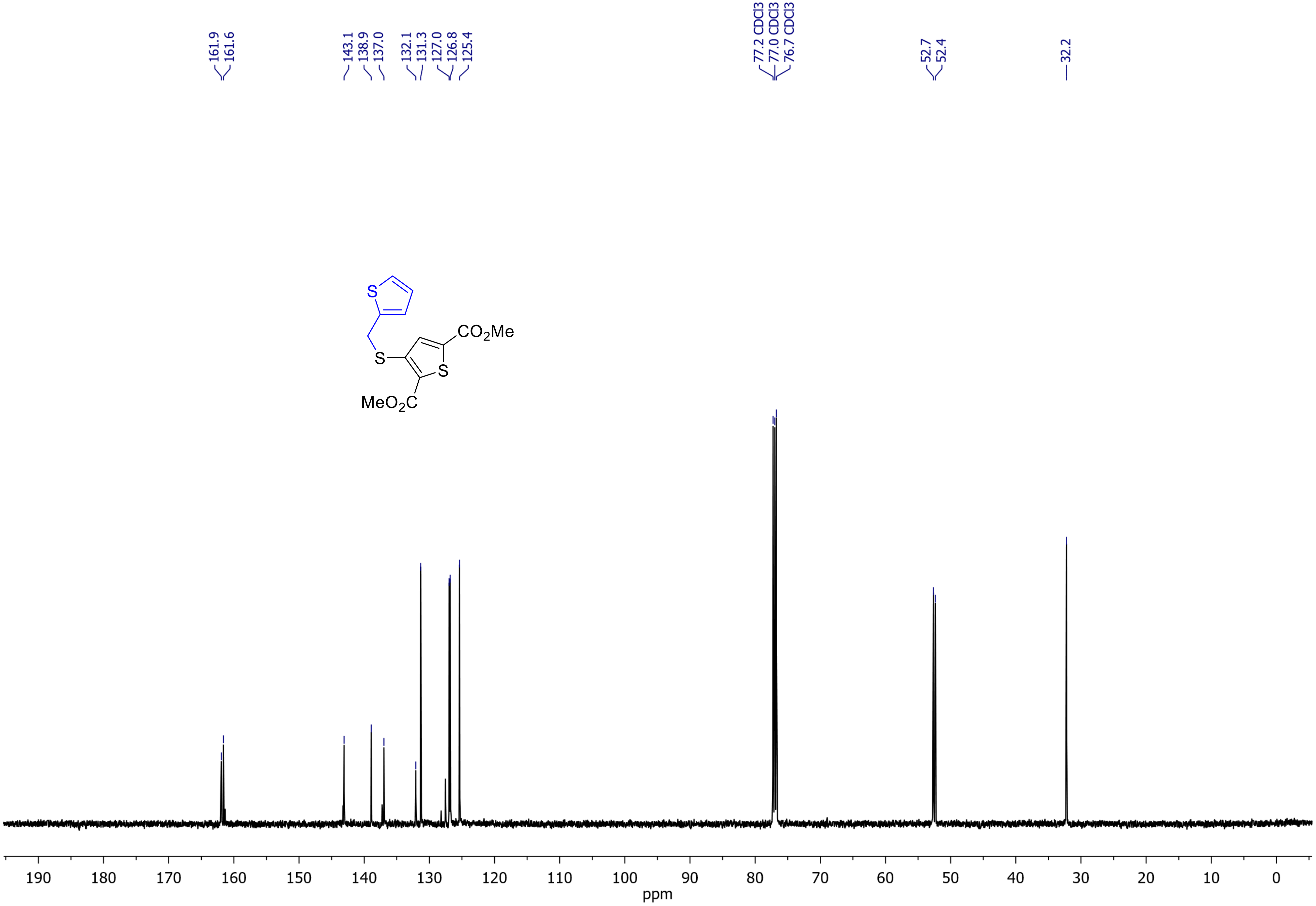

Dimethyl 3-[(cyanomethyl)thio]thiophene-2,5-dicarboxylate (**5a**)
^{1}H NMR (400 MHz, $CDCl_3$)

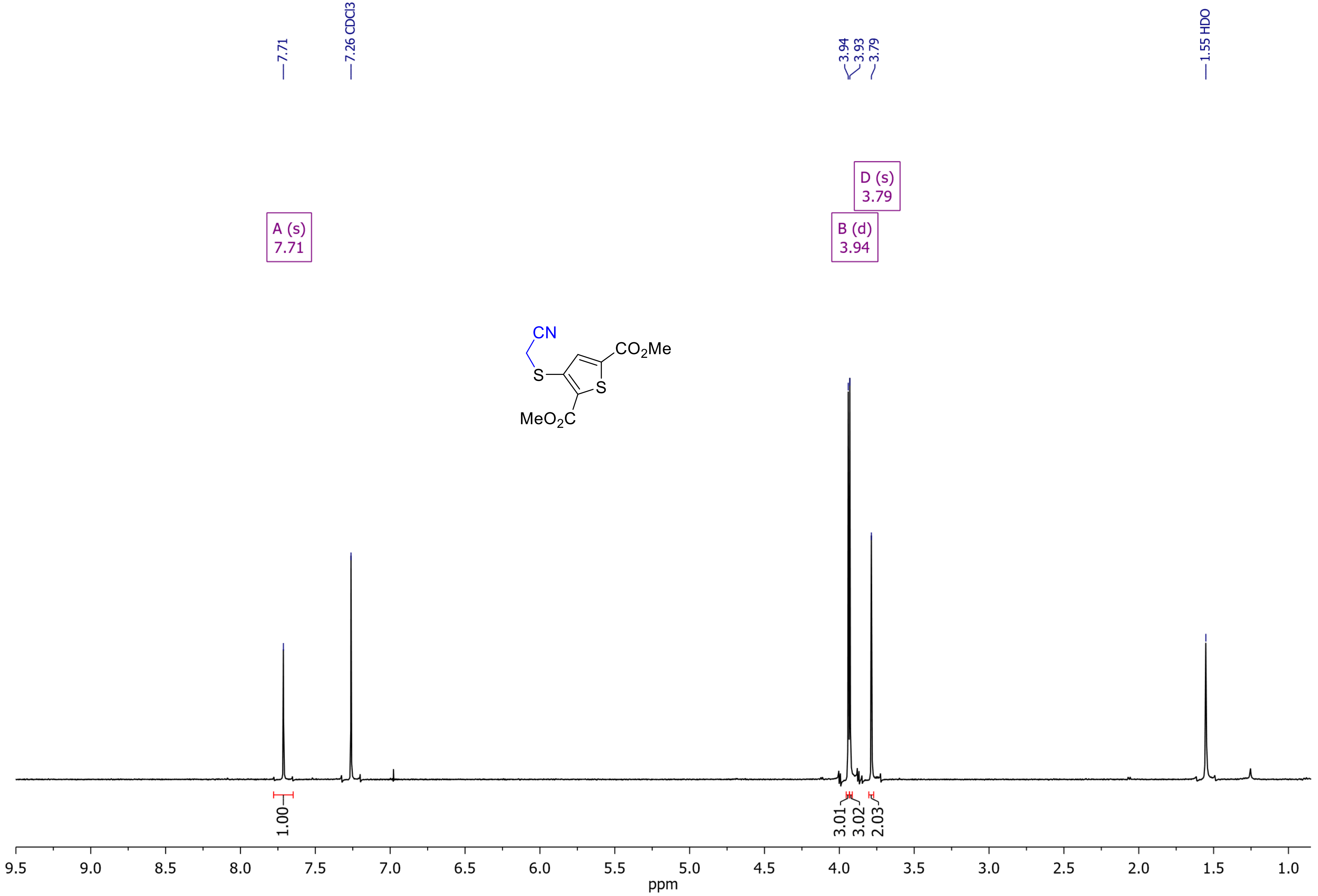

Dimethyl 3-[(cyanomethyl)thio]thiophene-2,5-dicarboxylate (**5a**)
^{13}C NMR (126 MHz, $CDCl_3$)

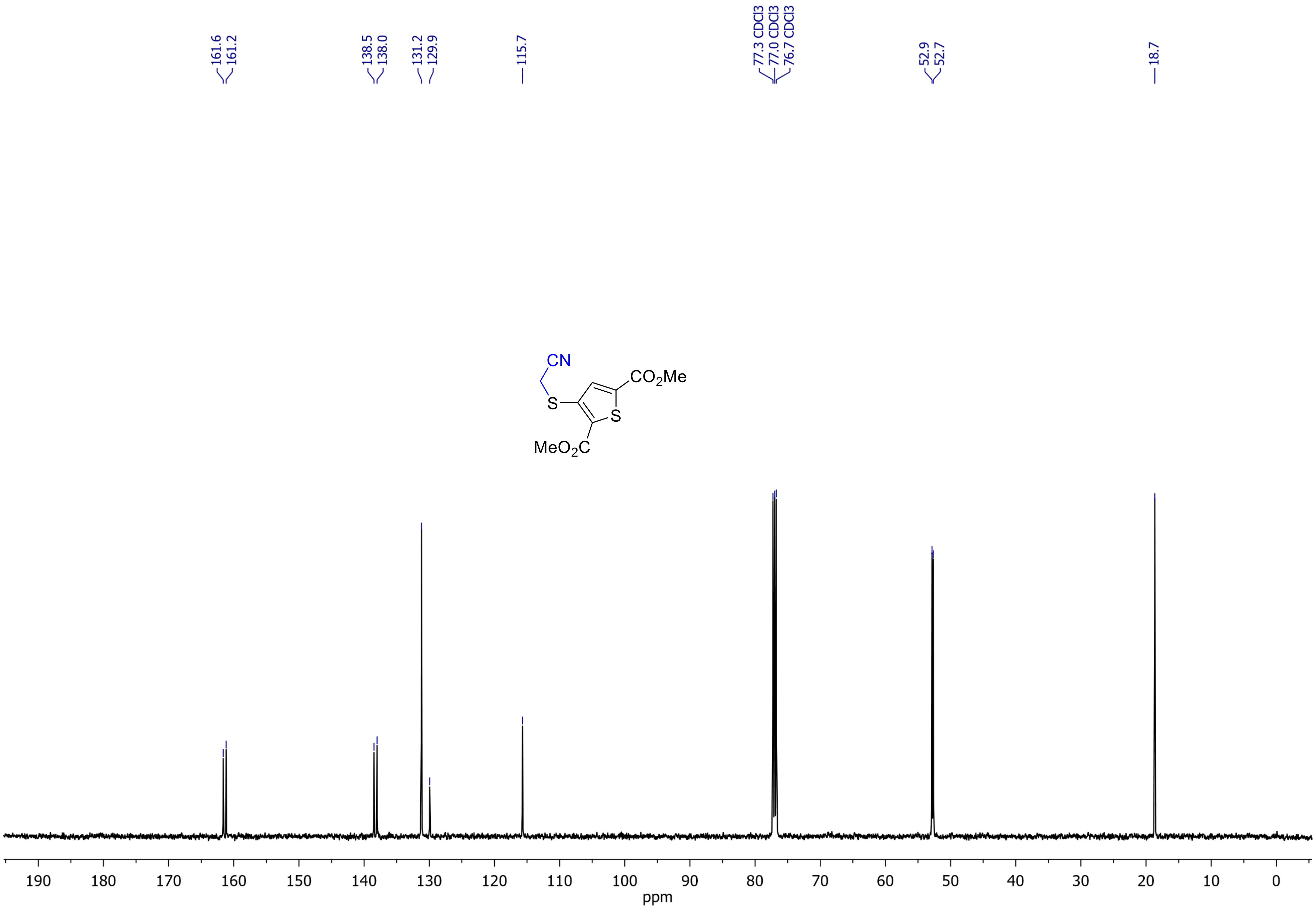

Dimethyl 3-[(2-oxo-2-phenylethyl)thio]thiophene-2,5-dicarboxylate (**6a**)
^{1}H NMR (400 MHz, $CDCl_3$)

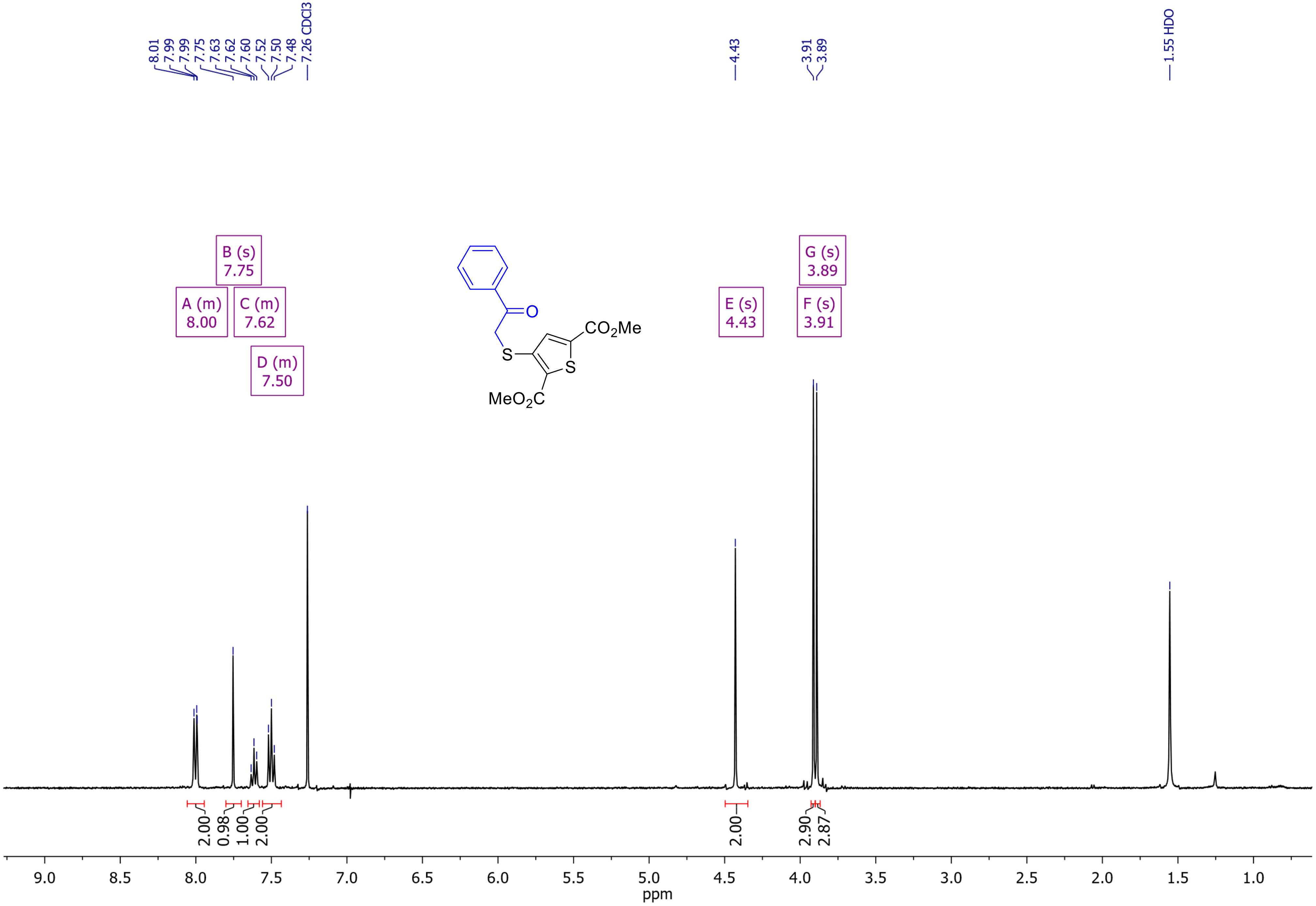

Dimethyl 3-[(2-oxo-2-phenylethyl)thio]thiophene-2,5-dicarboxylate (**6a**)
^{13}C NMR (126 MHz, $CDCl_3$)

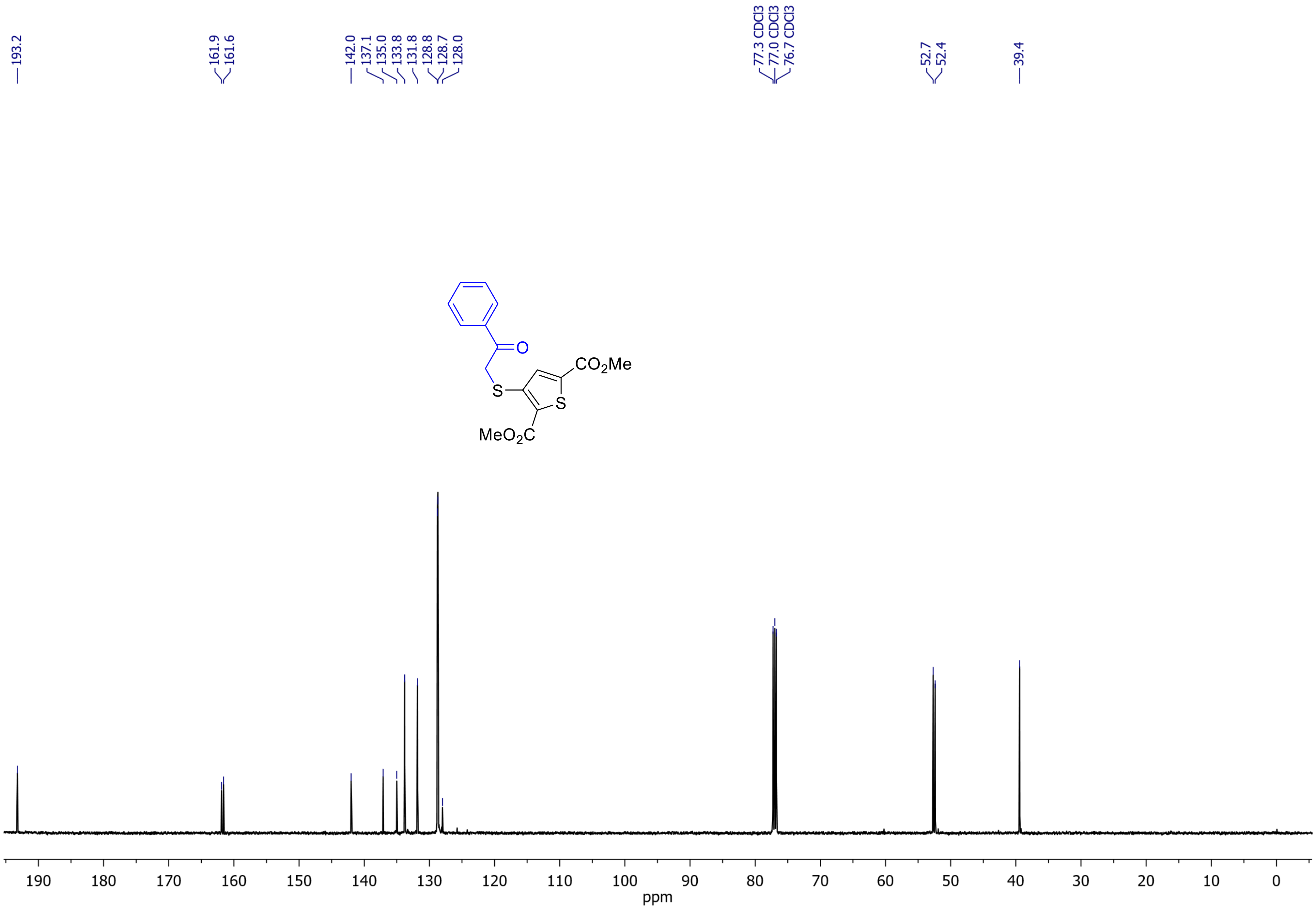

Dimethyl 3-{[2-(4-fluorophenyl)-2-oxoethyl]thio}thiophene-2,5-dicarboxylate (**6b**)

^{1}H NMR (400 MHz, $CDCl_3$)

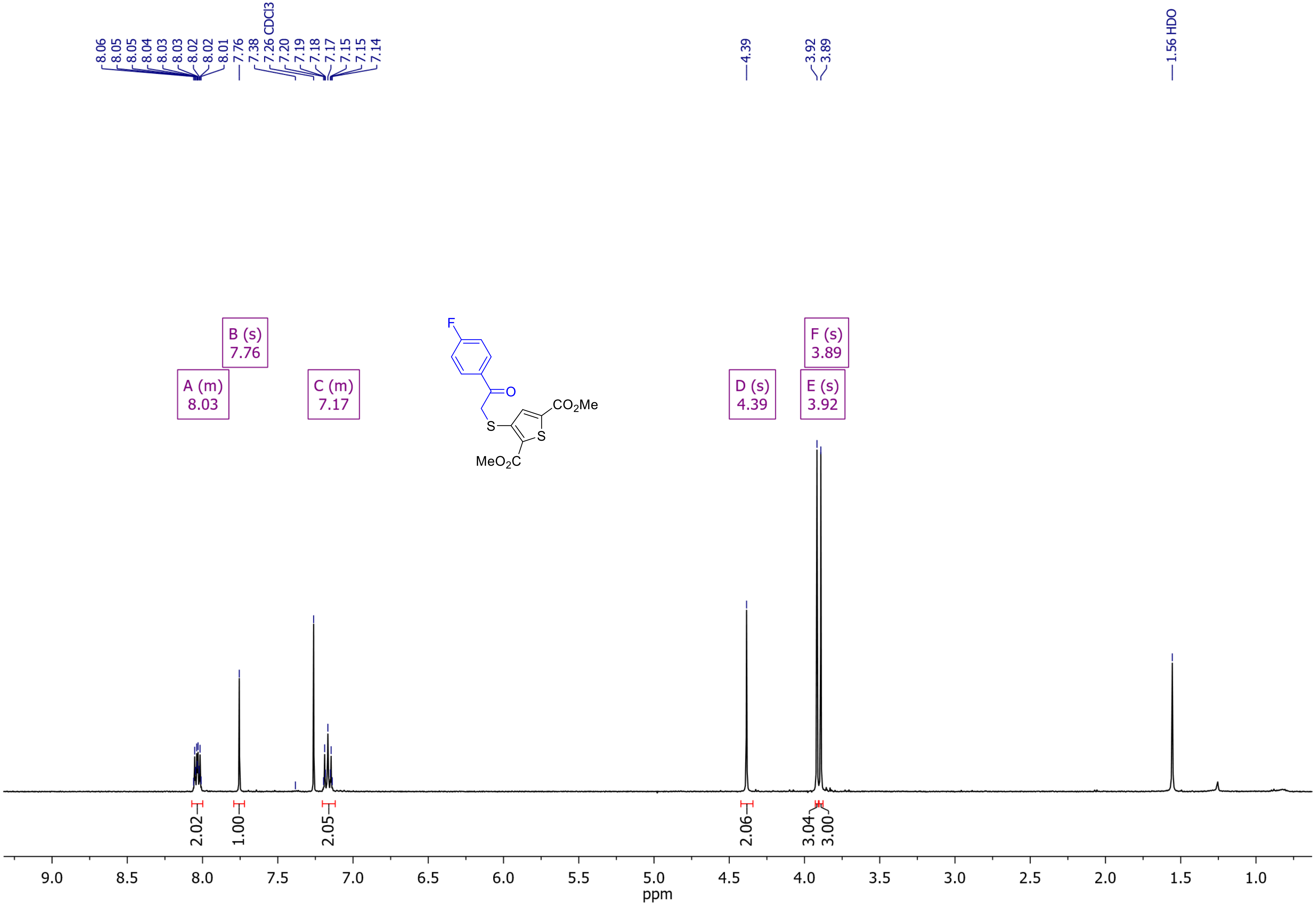

Dimethyl 3-{[2-(4-fluorophenyl)-2-oxoethyl]thio}thiophene-2,5-dicarboxylate (**6b**)
^{19}F NMR (376 MHz, $CDCl_3$)

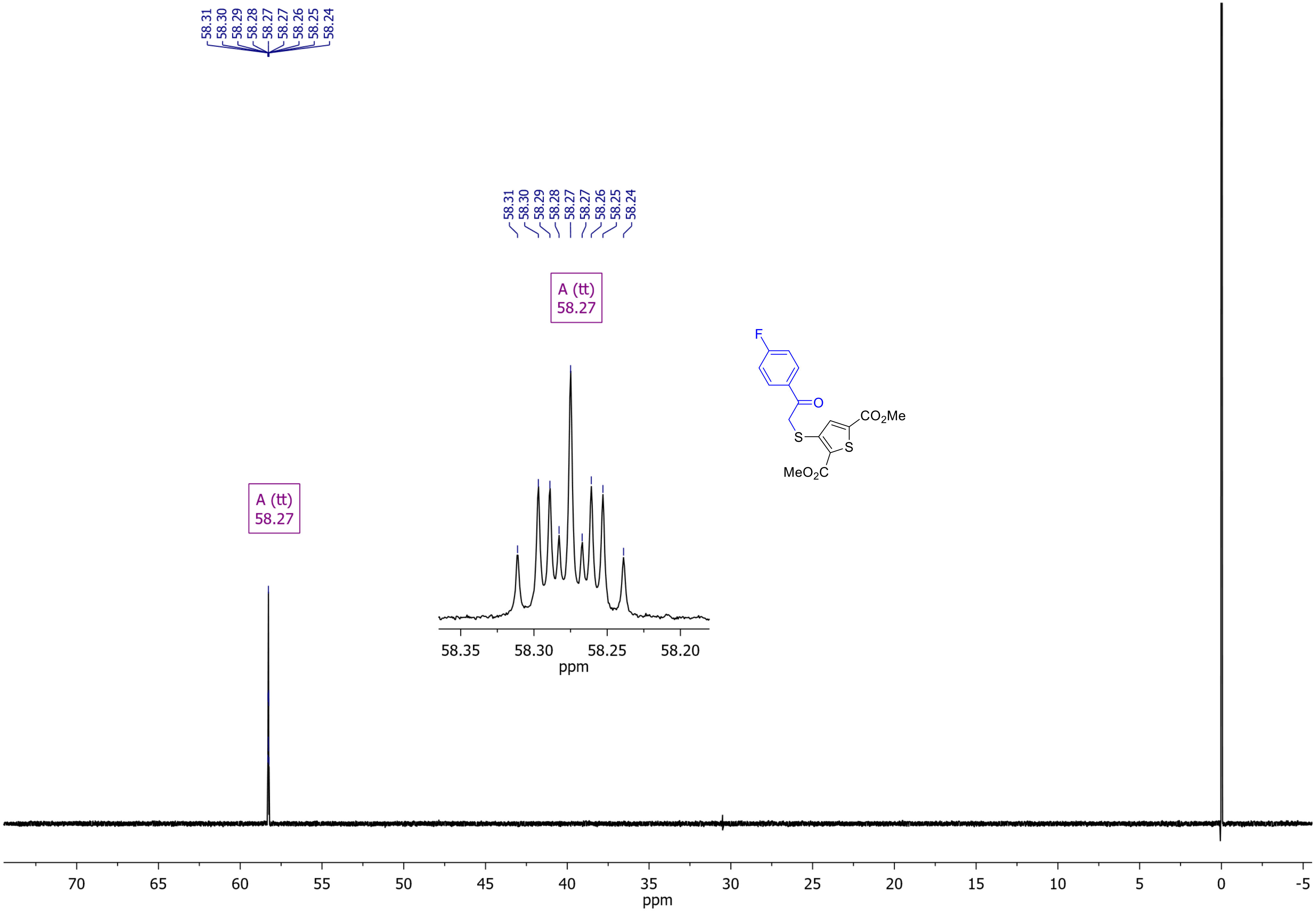

Dimethyl 3-{[2-(4-fluorophenyl)-2-oxoethyl]thio}thiophene-2,5-dicarboxylate (**6b**)
^{13}C NMR (126 MHz, $CDCl_3$)

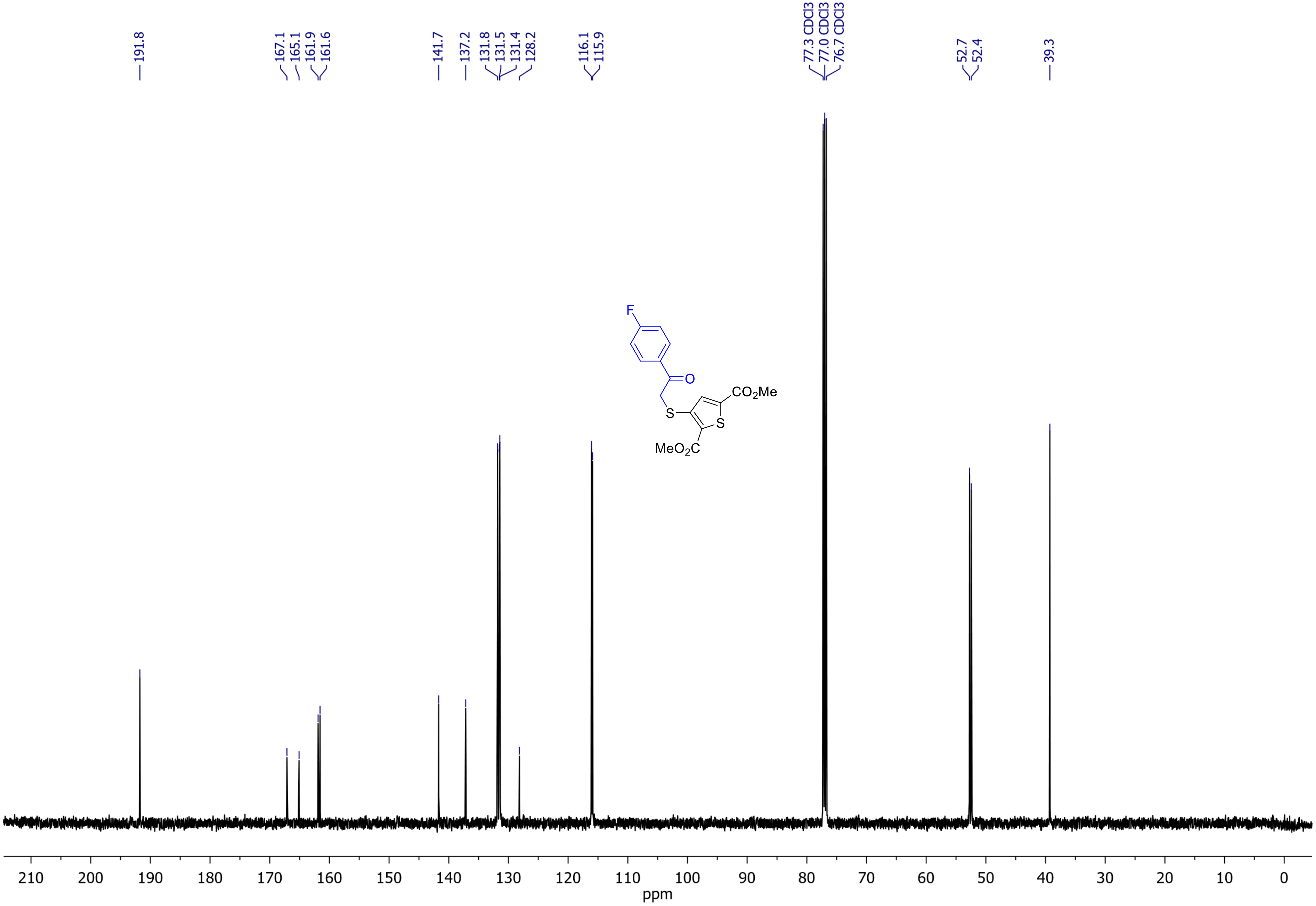

Dimethyl 3-{[2-oxo-2-(thiophen-2-yl)ethyl]thio}thiophene-2,5-dicarboxylate (**6c**)

^{1}H NMR (400 MHz, $CDCl_3$)

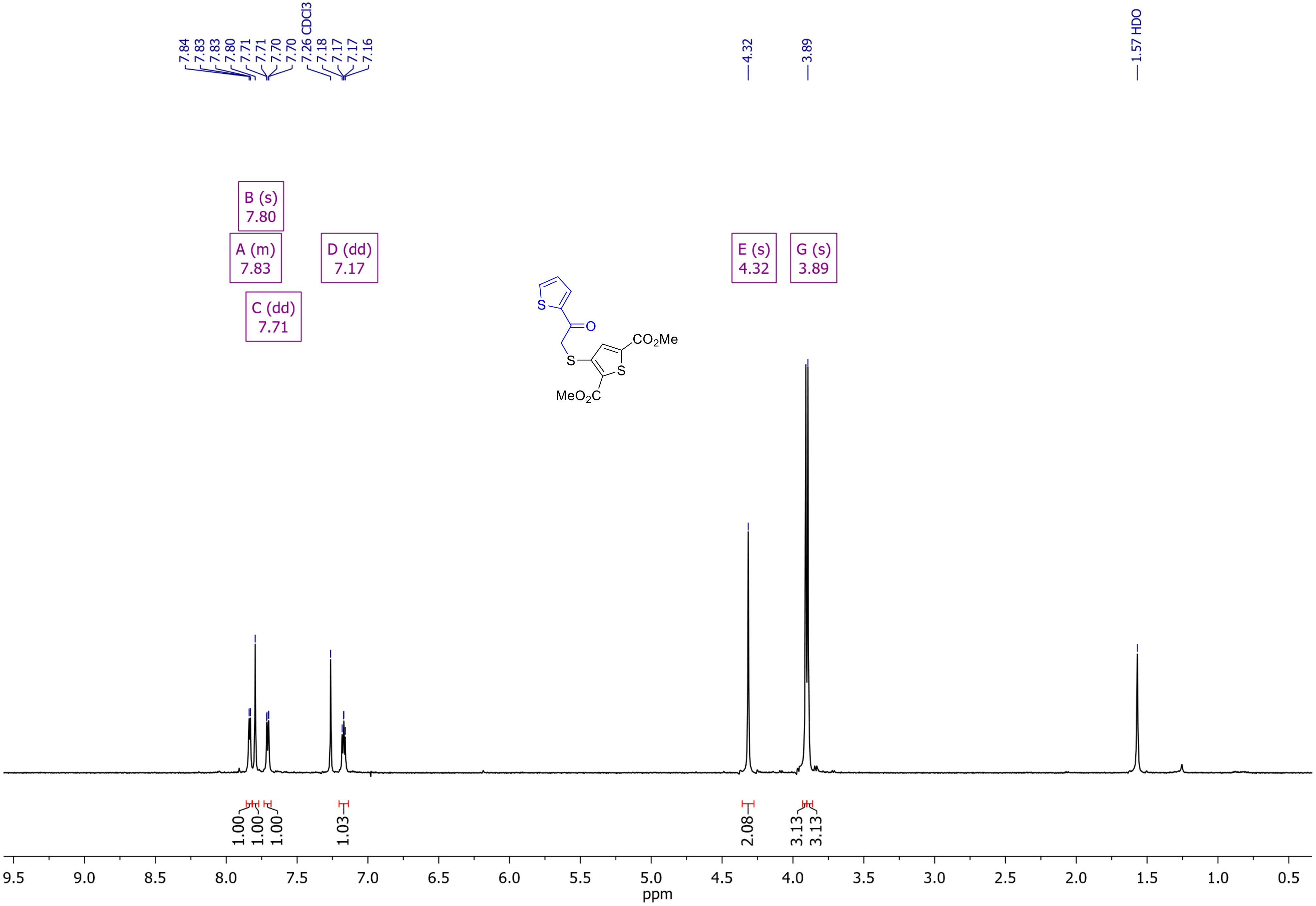

Dimethyl 3-{[2-oxo-2-(thiophen-2-yl)ethyl]thio}thiophene-2,5-dicarboxylate (**6c**)
^{13}C NMR (126 MHz, $CDCl_3$)

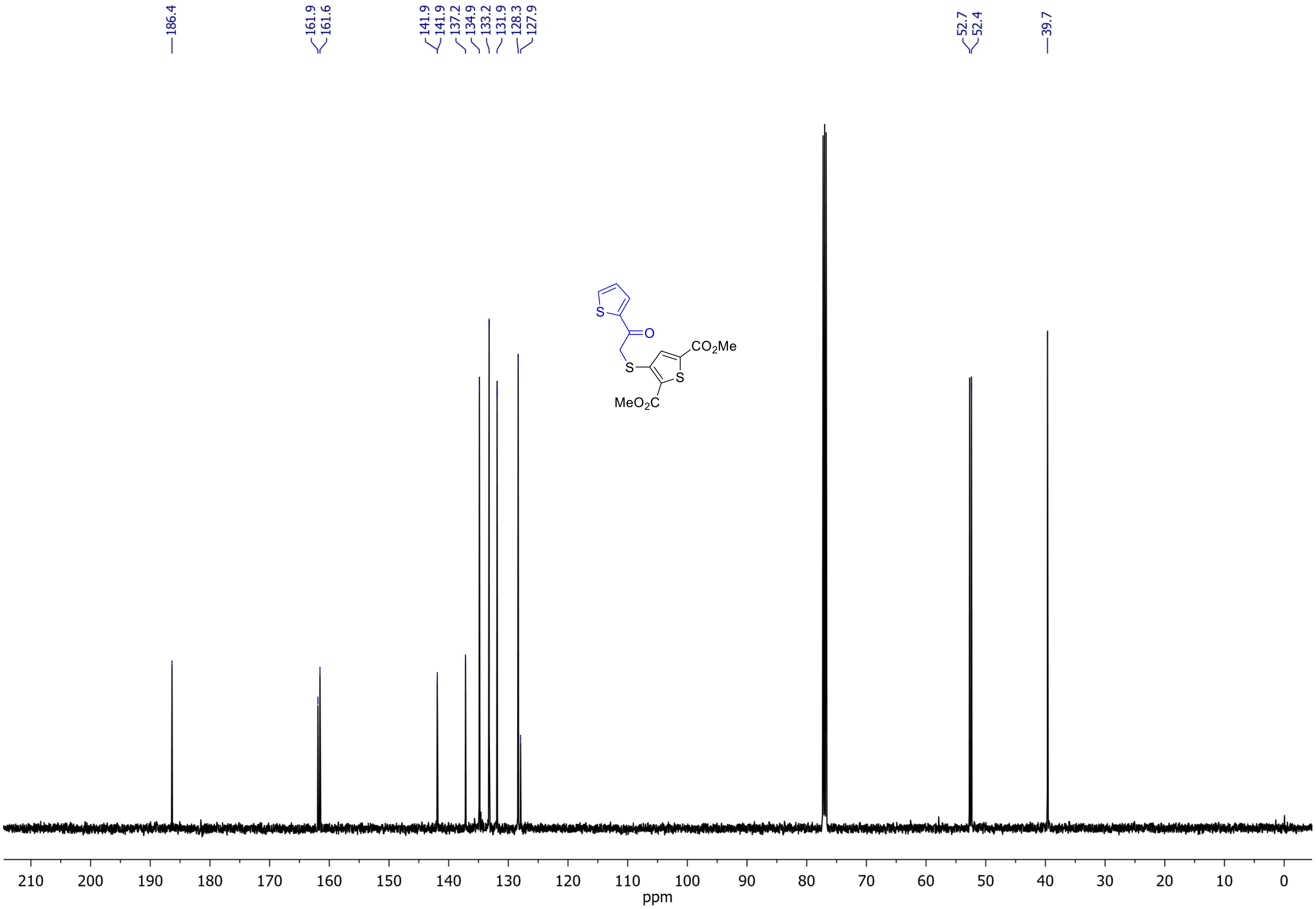

Methyl 5-(4-cyanophenyl)-6-hydroxythieno[3,2-*b*]thiophene-2-carboxylate (**7a**)

^{1}H NMR (400 MHz, DMSO-d_6)

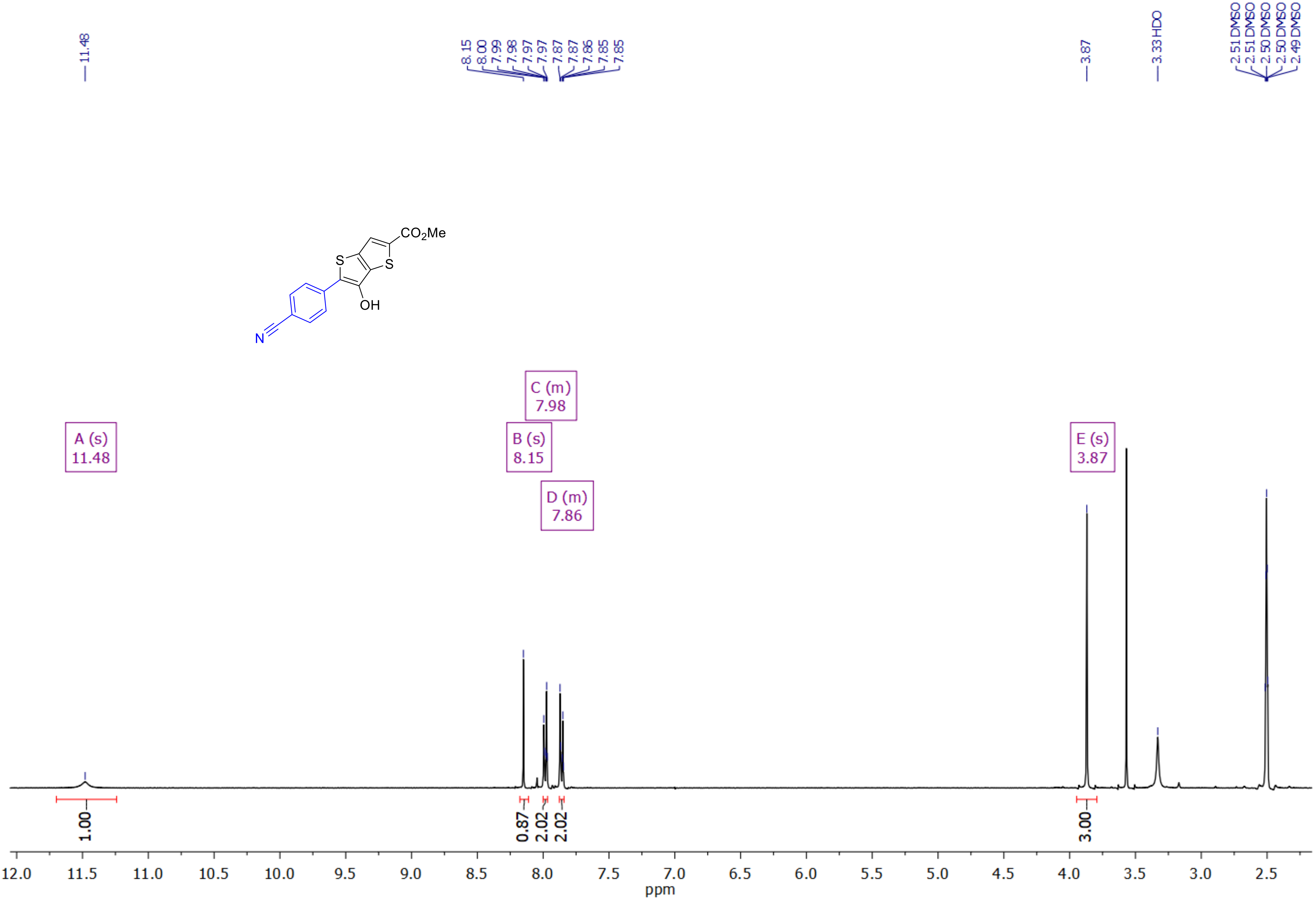

Methyl 5-(4-cyanophenyl)-6-hydroxythieno[3,2-*b*]thiophene-2-carboxylate (**7a**)
^{13}C NMR (126 MHz, DMSO-d_6)

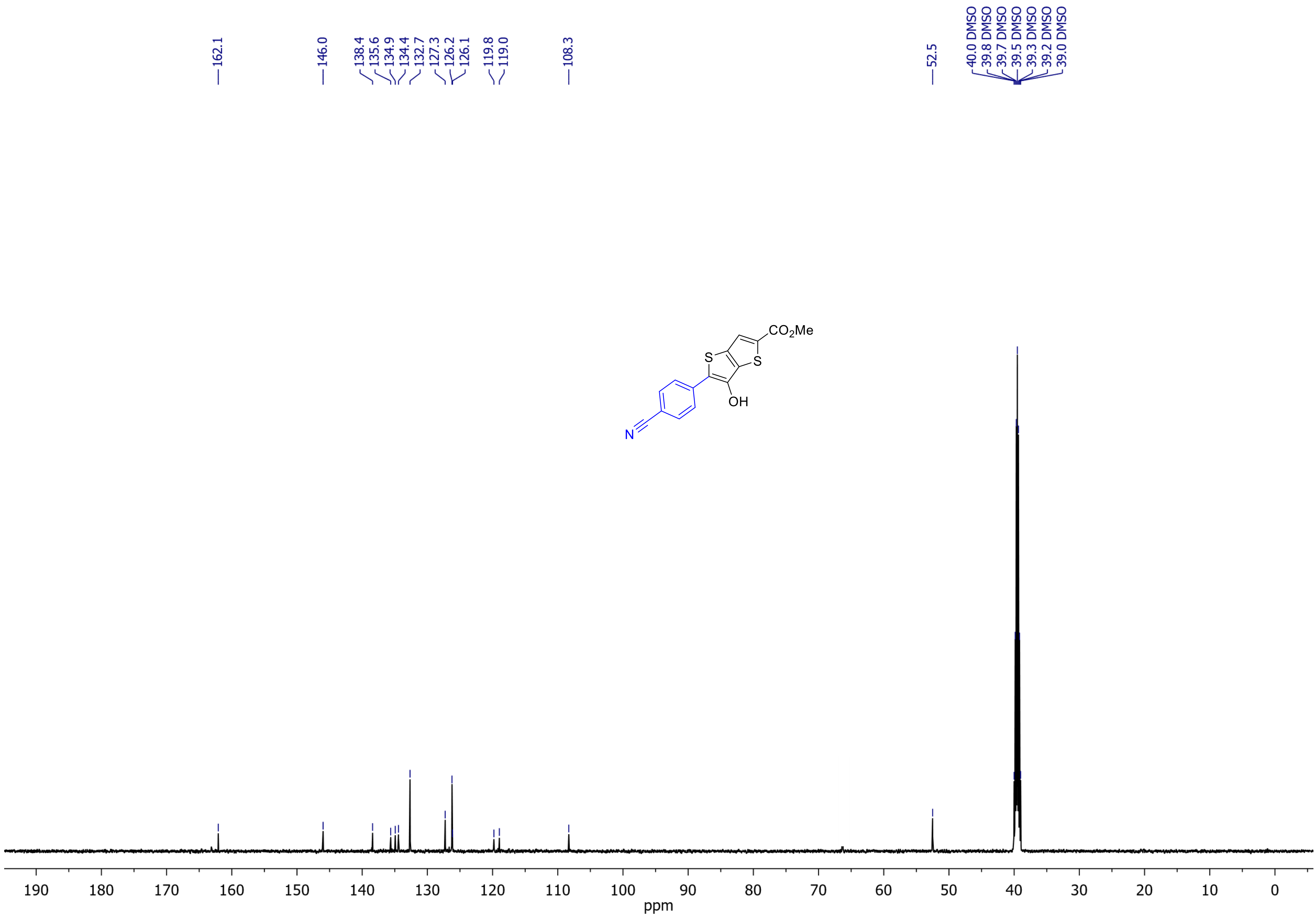

Methyl 6-hydroxy-5-[4-(methoxycarbonyl)phenyl]thieno[3,2-*b*]thiophene-2-carboxylate (**7b**)
^{1}H NMR (400 MHz, DMSO-d_6)

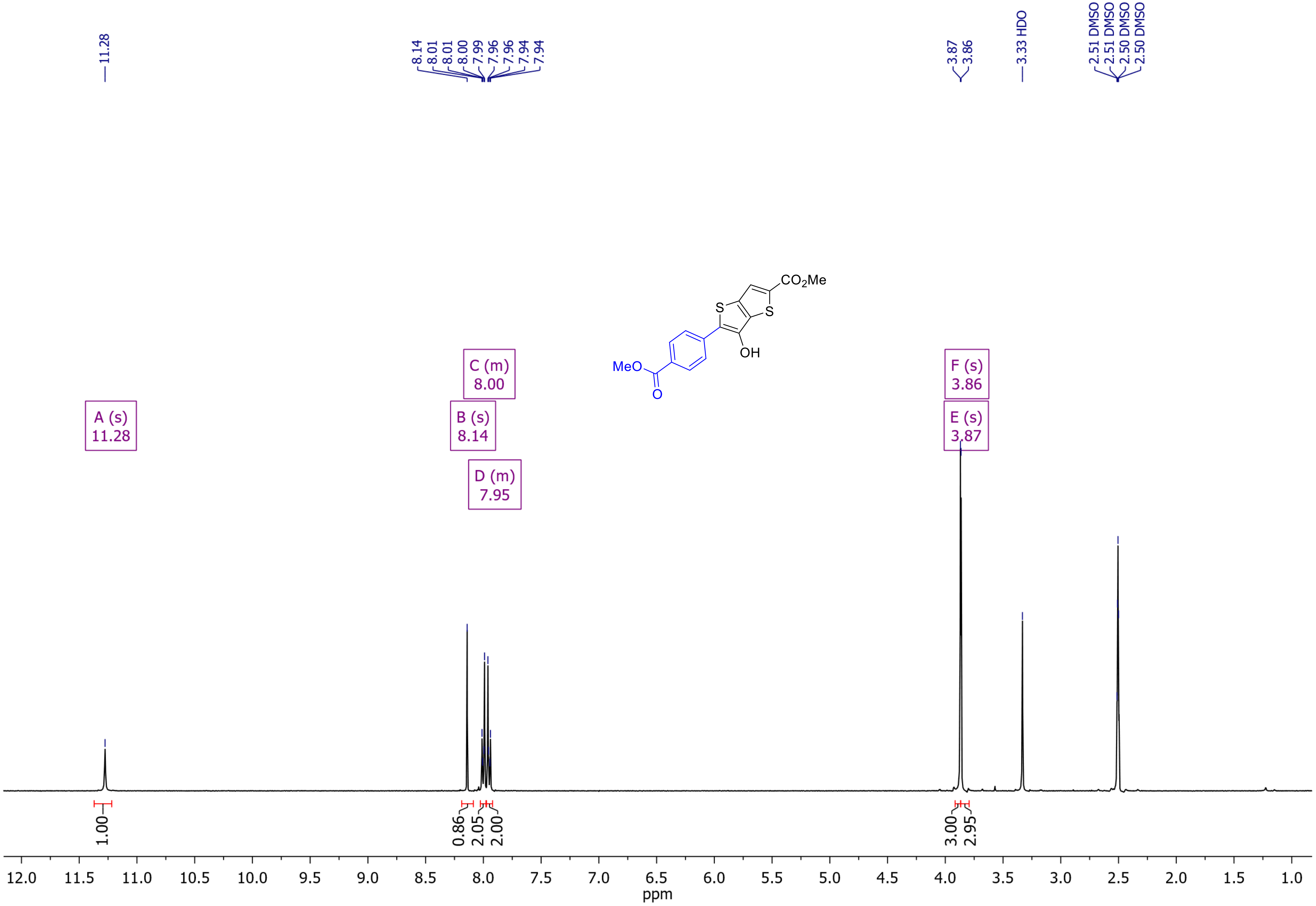

Methyl 6-hydroxy-5-[4-(methoxycarbonyl)phenyl]thieno[3,2-*b*]thiophene-2-carboxylate (**7b**)

^{13}C NMR (126 MHz, DMSO-d_6)

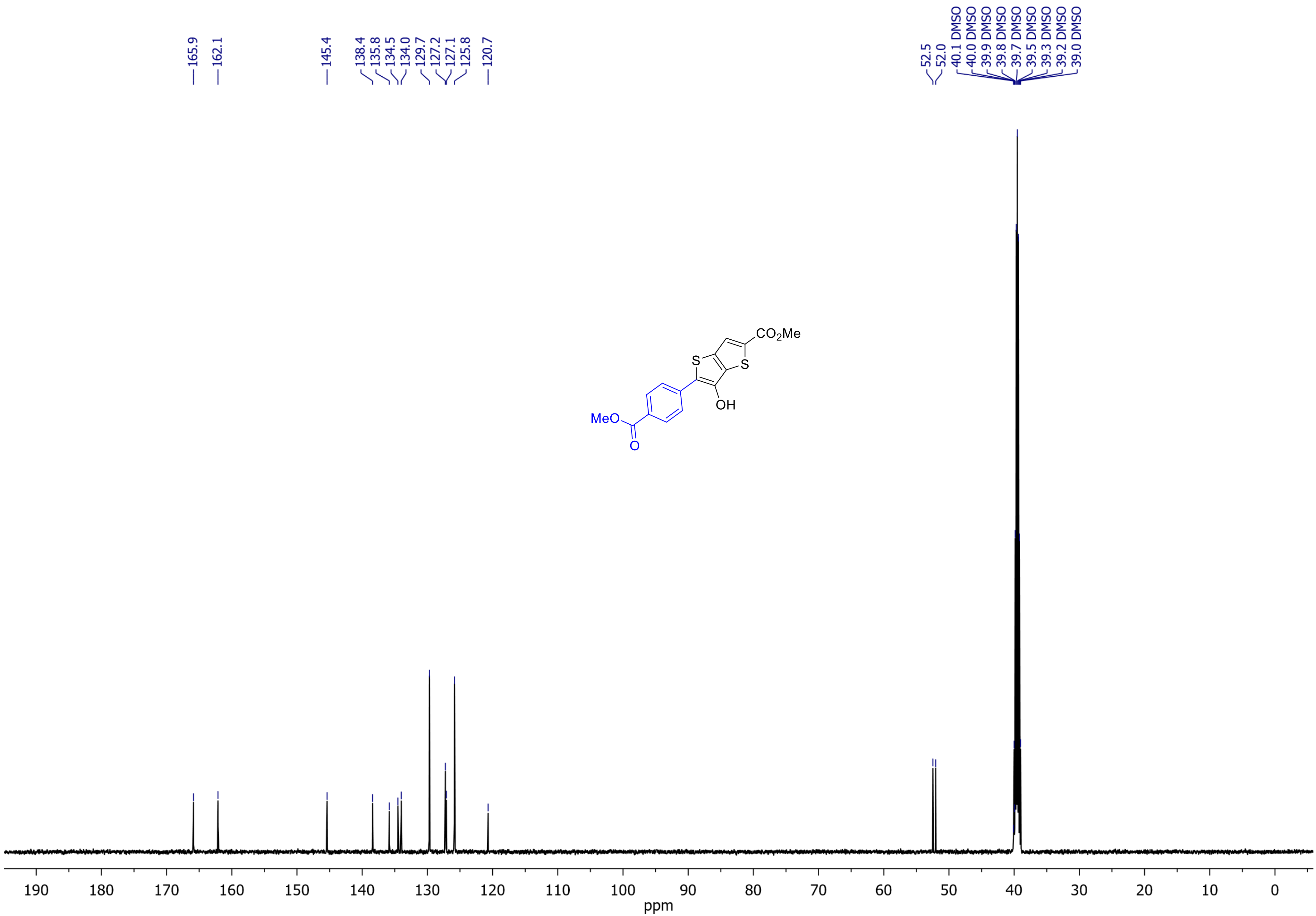

Methyl 5-cyano-6-hydroxythieno[3,2-*b*]thiophene-2-carboxylate (**8a**)

^{1}H NMR (400 MHz, DMSO-d_6)

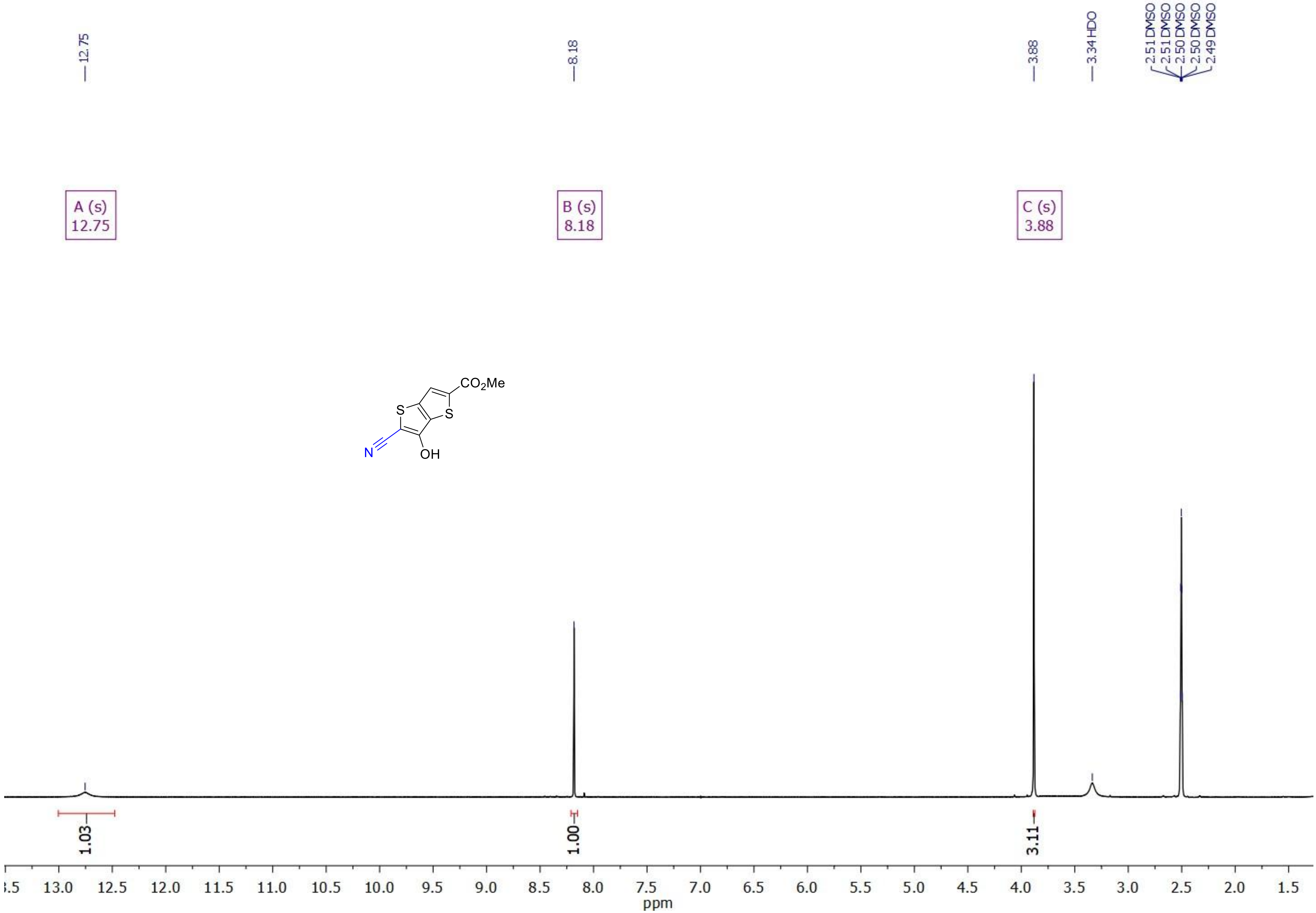

Methyl 5-cyano-6-hydroxythieno[3,2-*b*]thiophene-2-carboxylate (**8a**)

^{13}C NMR (126 MHz, DMSO-d_6)

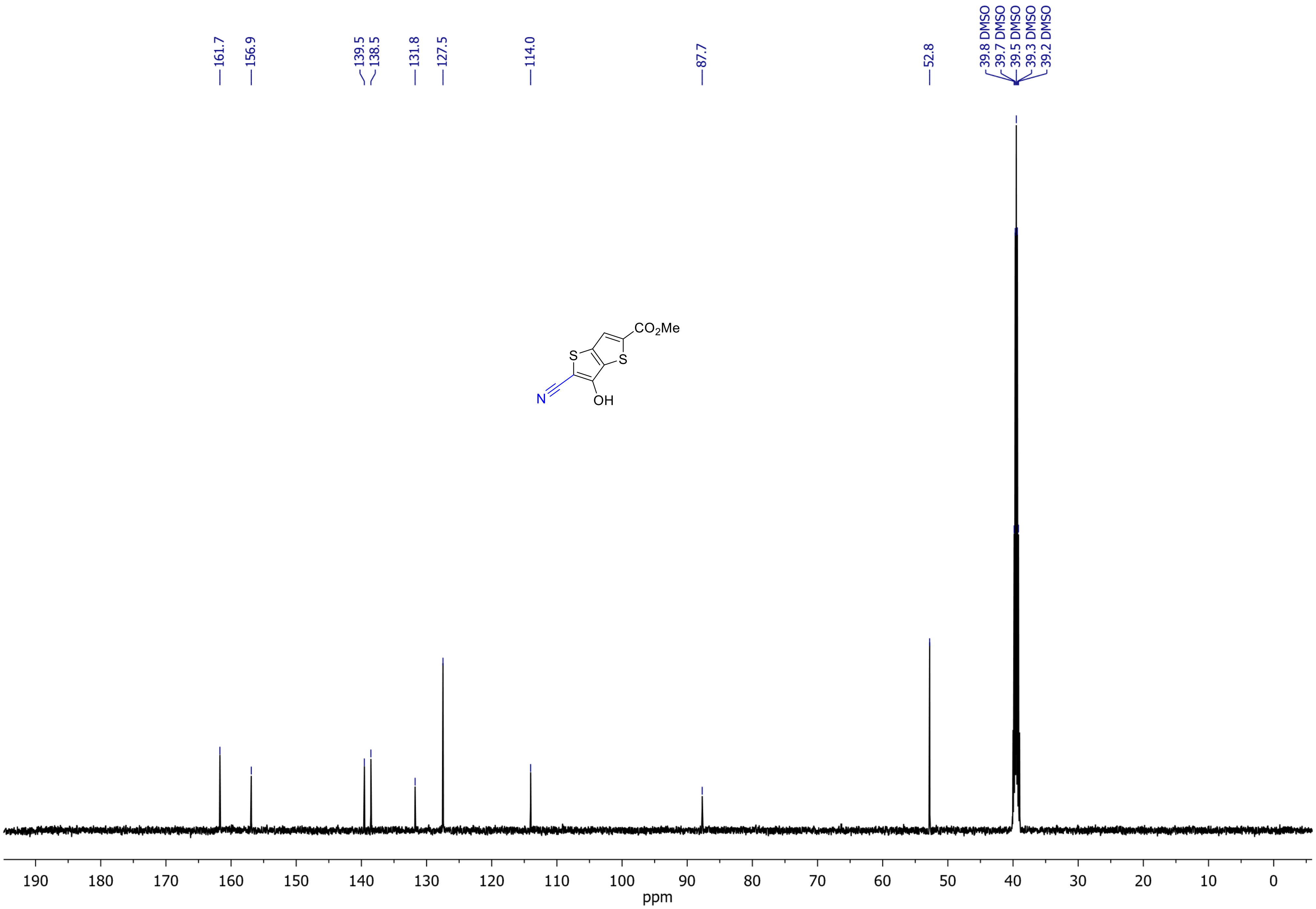

5-(4-Fluorobenzoyl)-6-hydroxythieno[3,2-*b*]thiophene-2-carboxylic acid (**9bA**)

^{1}H NMR (400 MHz, DMSO-d_6)

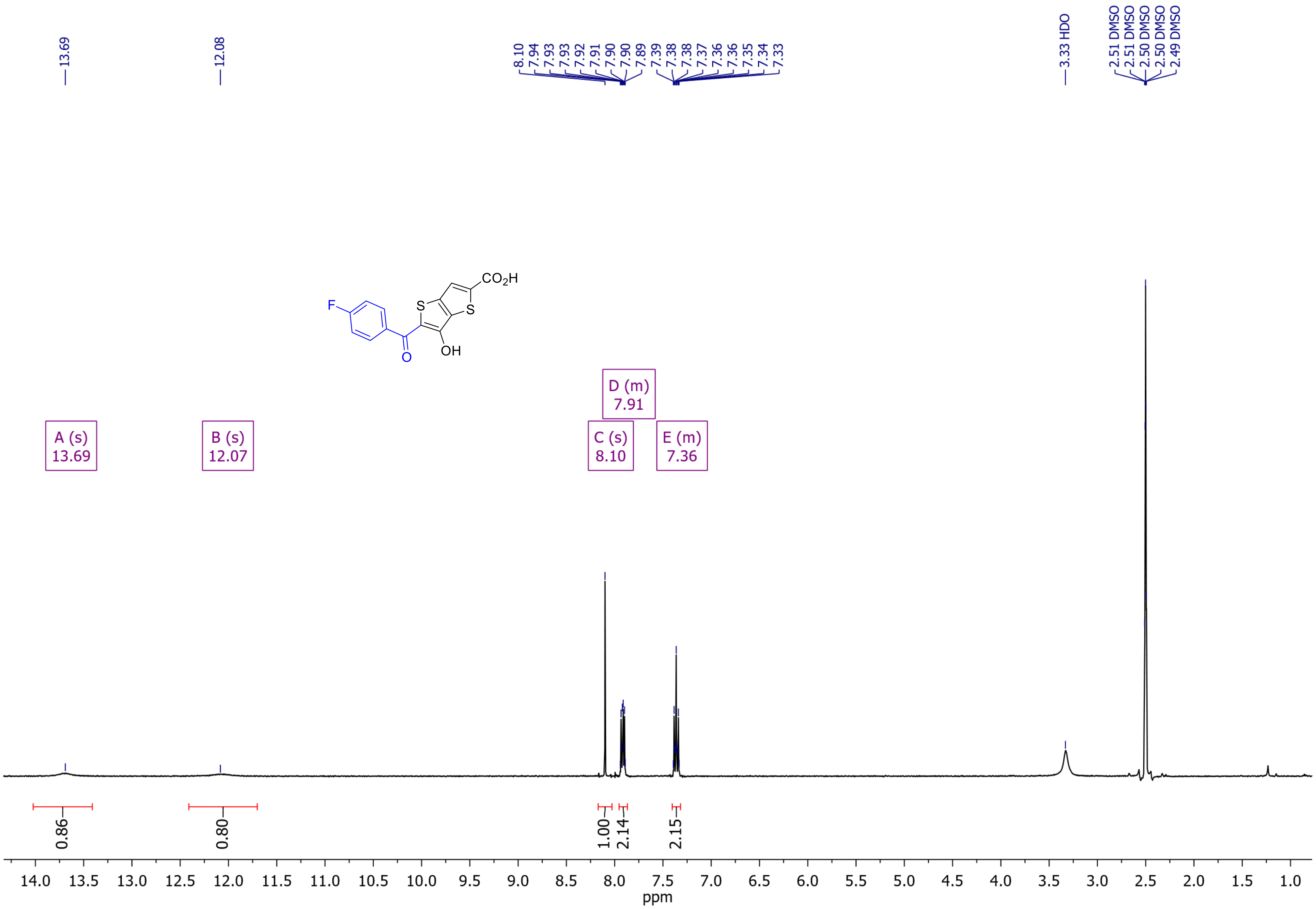

5-(4-Fluorobenzoyl)-6-hydroxythieno[3,2-*b*]thiophene-2-carboxylic acid (**9bA**)
^{19}F NMR (376 MHz, DMSO-d_6)

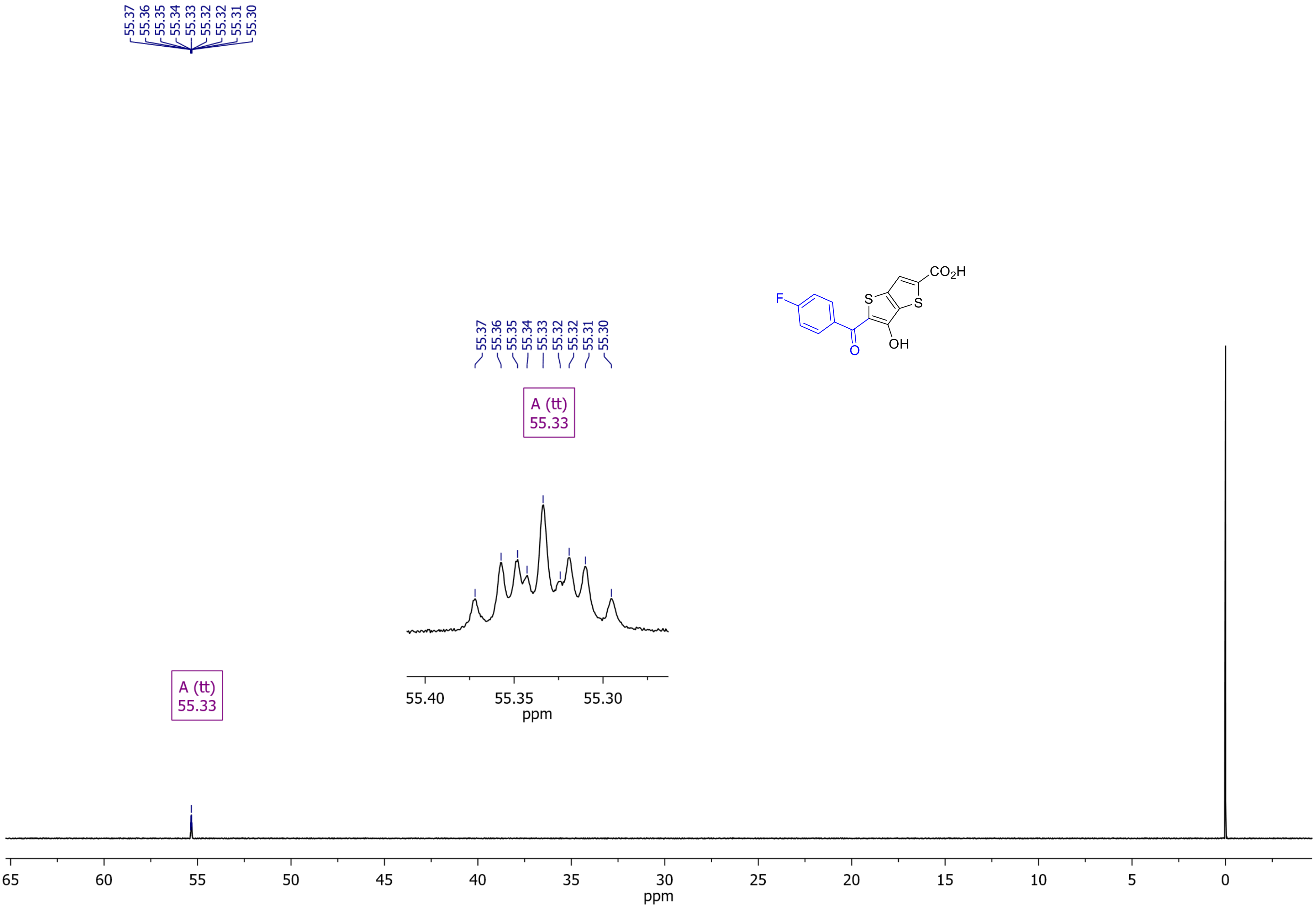

5-(4-Fluorobenzoyl)-6-hydroxythieno[3,2-*b*]thiophene-2-carboxylic acid (**9bA**)
^{13}C NMR (126 MHz, DMSO-d_6)

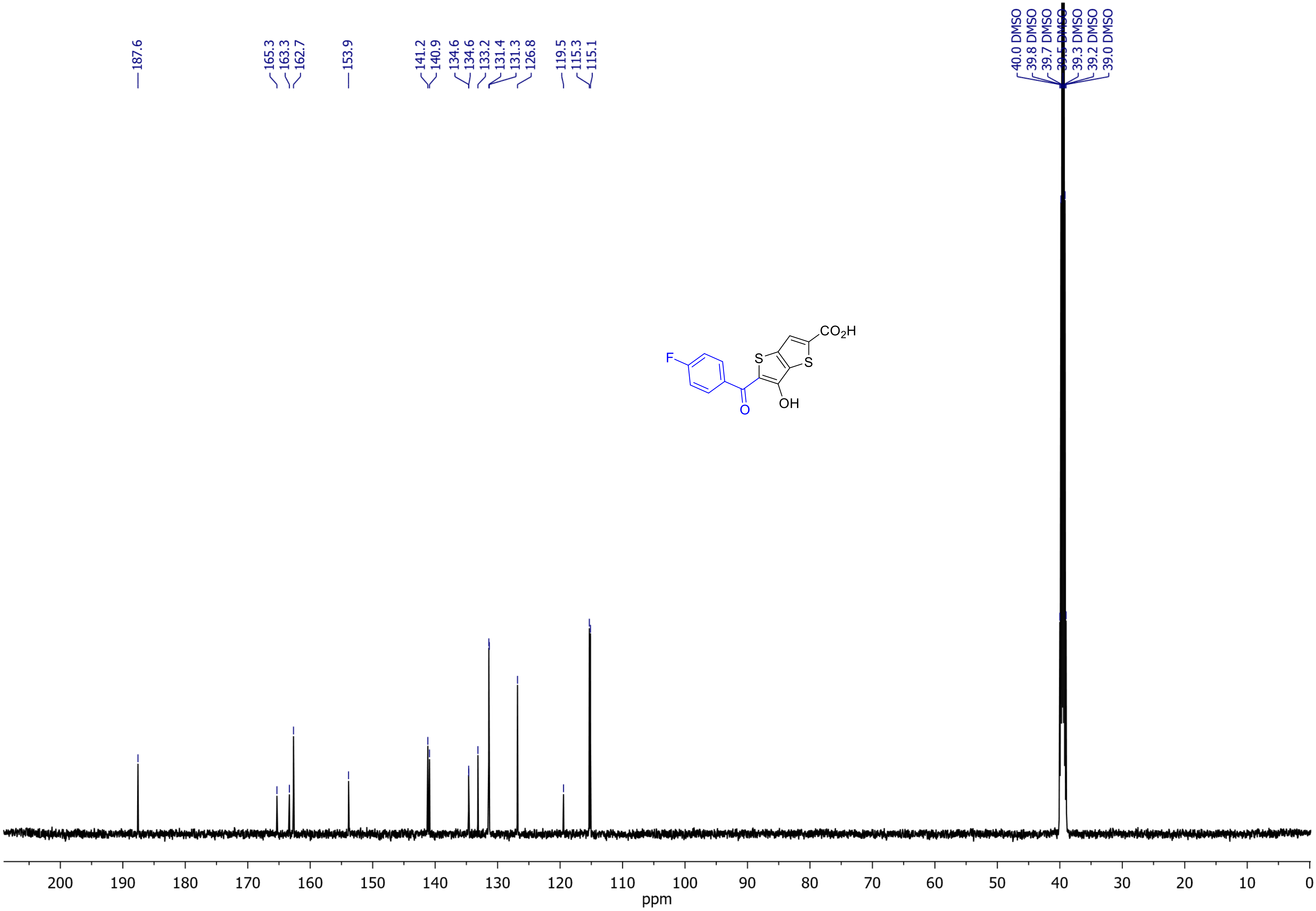

Methyl 5-benzoyl-6-hydroxythieno[3,2-*b*]thiophene-2-carboxylate (**9a**)
^{1}H NMR (400 MHz, DMSO-d_6)

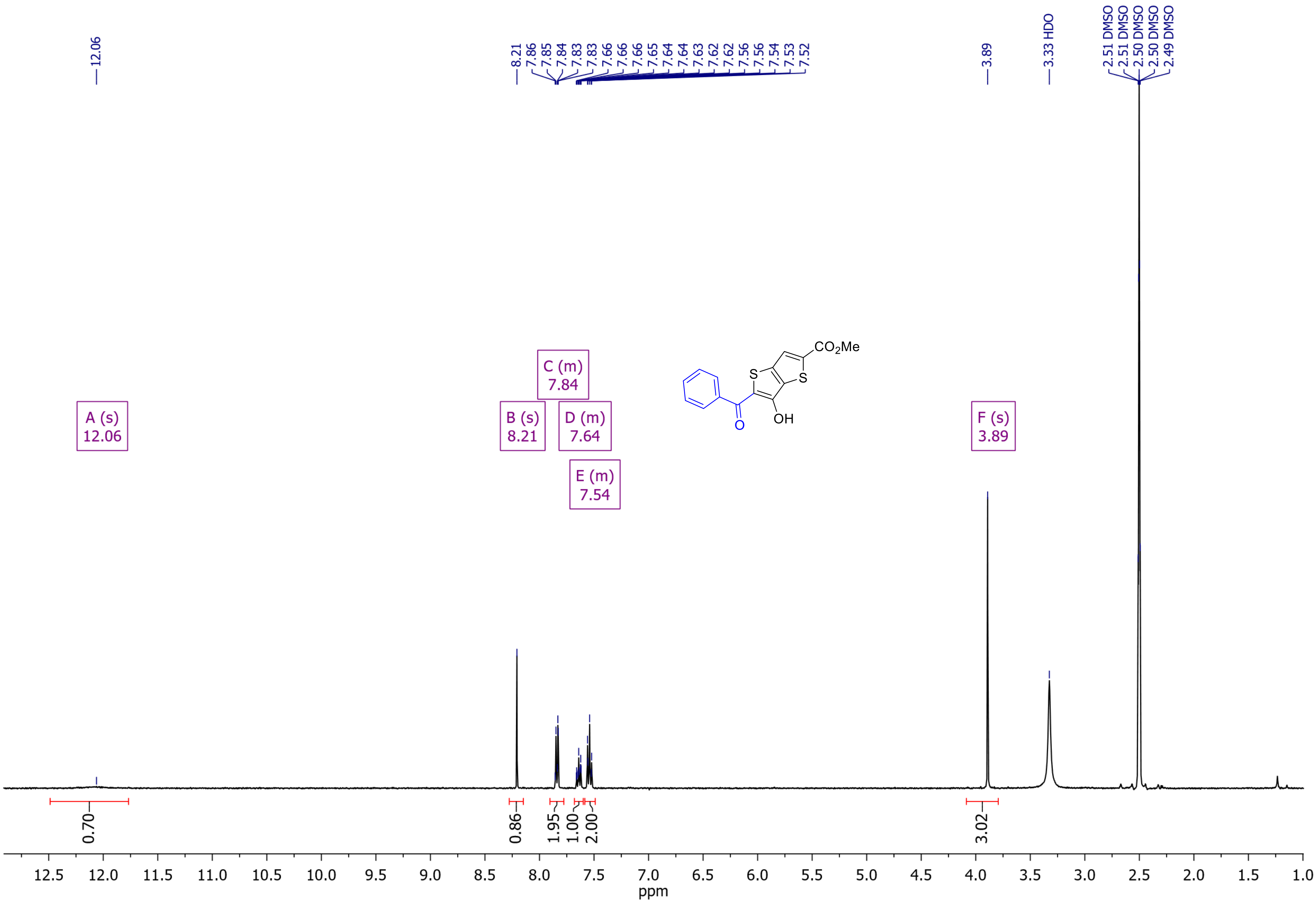

Methyl 5-benzoyl-6-hydroxythieno[3,2-*b*]thiophene-2-carboxylate (**9a**)

^{13}C NMR (126 MHz, DMSO-d_6)

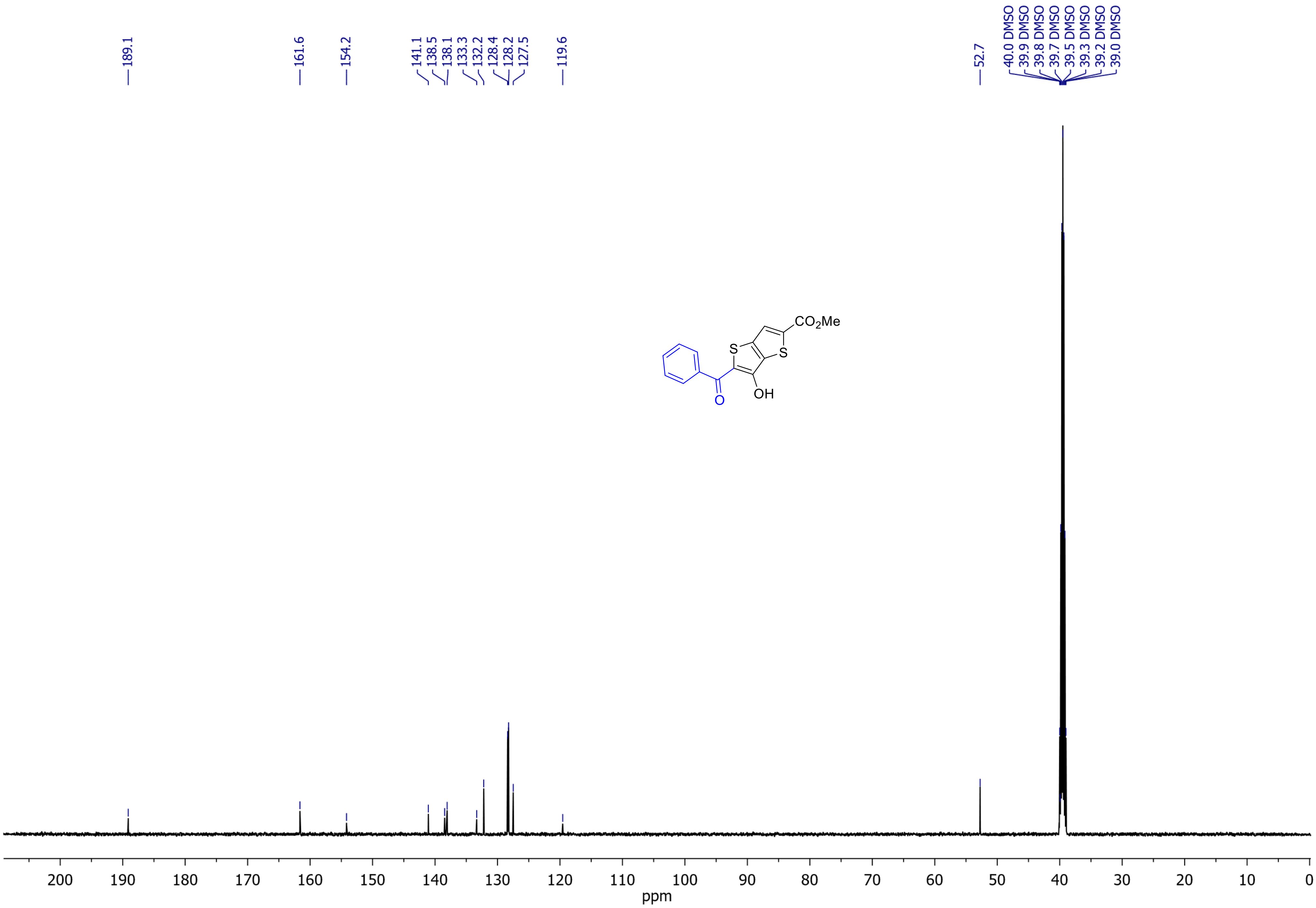

Methyl 5-(4-fluorobenzoyl)-6-hydroxythieno[3,2-*b*]thiophene-2-carboxylate (**9b**)
^{1}H NMR (400 MHz, DMSO-d_6)

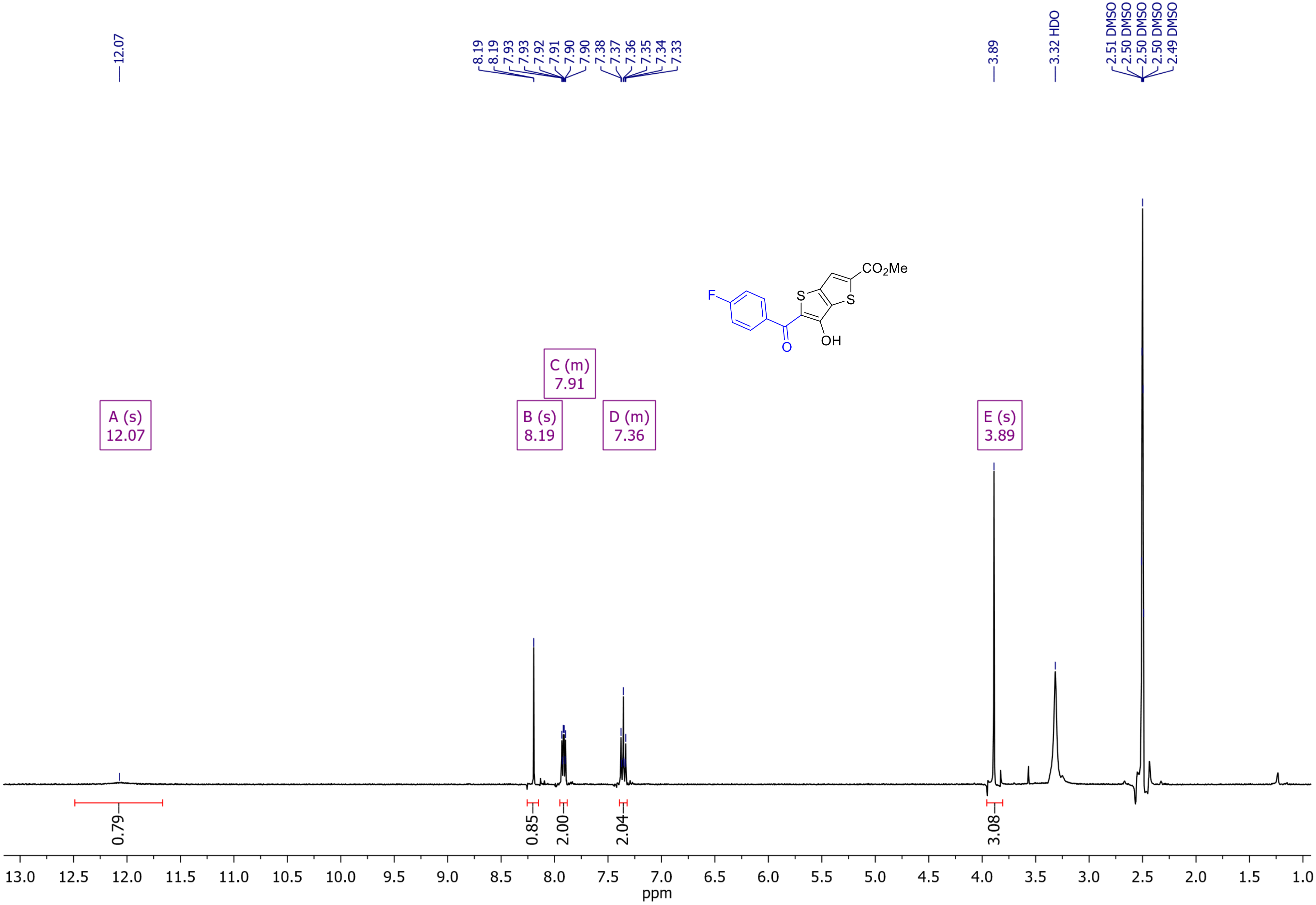

Methyl 5-(4-fluorobenzoyl)-6-hydroxythieno[3,2-*b*]thiophene-2-carboxylate (**9b**)

^{19}F NMR (376 MHz, DMSO-*d*$_6$)

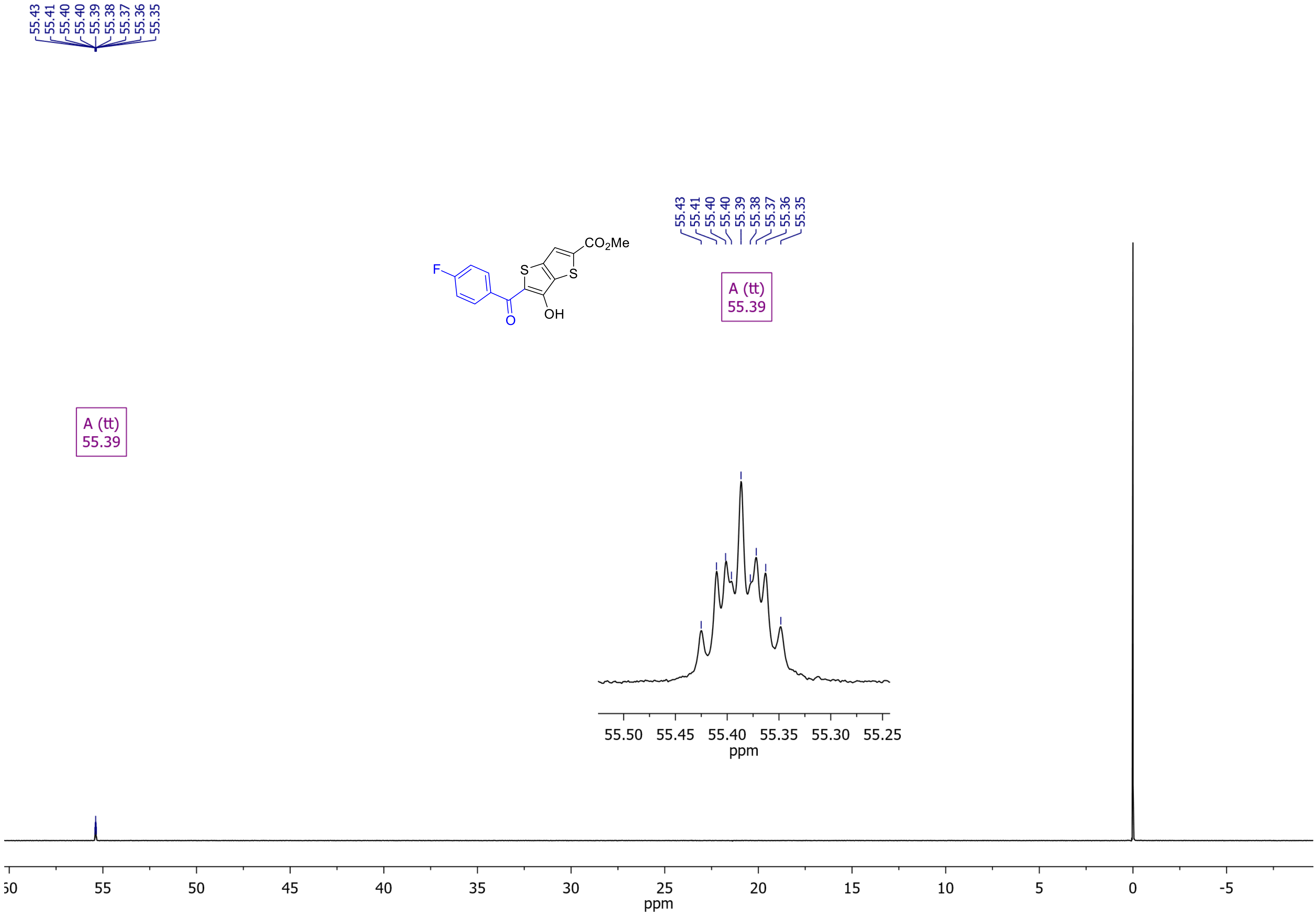

Methyl 5-(4-fluorobenzoyl)-6-hydroxythieno[3,2-*b*]thiophene-2-carboxylate (**9b**)
^{13}C NMR (126 MHz, DMSO-d_6)

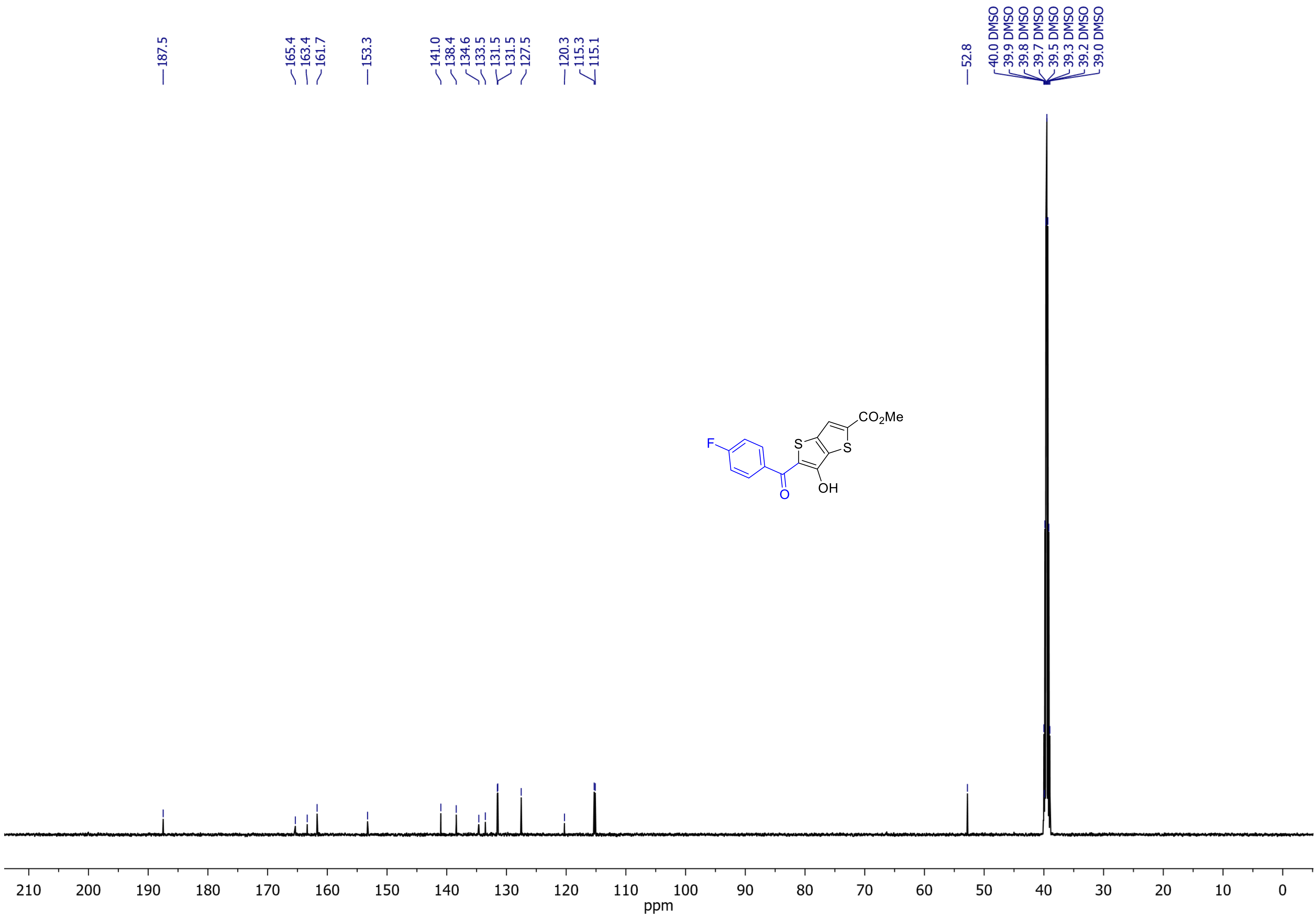

Methyl 6-hydroxy-5-(thiophene-2-carbonyl)thieno[3,2-*b*]thiophene-2-carboxylate (**9c**)

^{1}H NMR (400 MHz, $CDCl_3$)

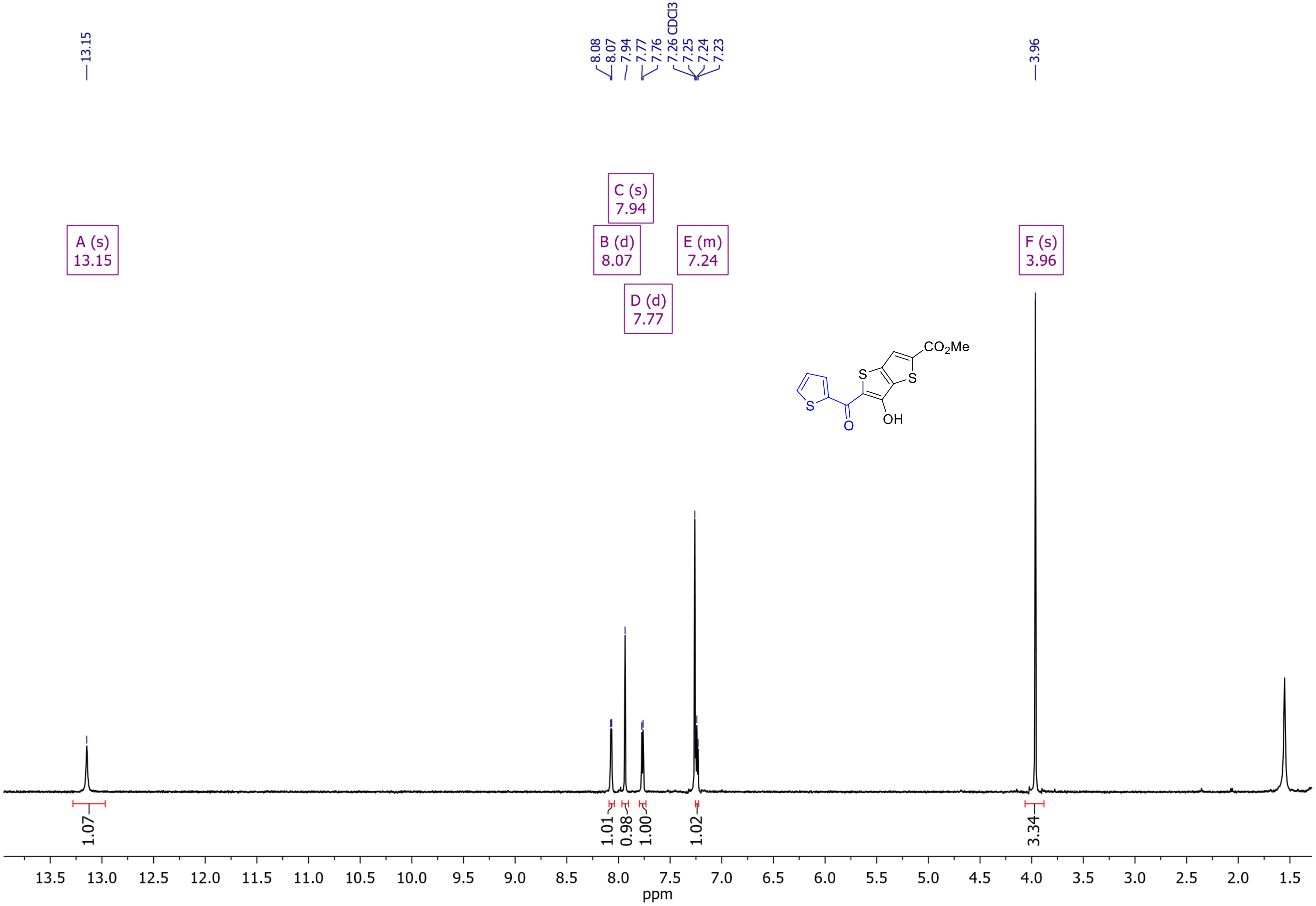

Methyl 6-hydroxy-5-(thiophene-2-carbonyl)thieno[3,2-*b*]thiophene-2-carboxylate (**9c**)

^{1}H NMR (500 MHz, DMSO-d_6)

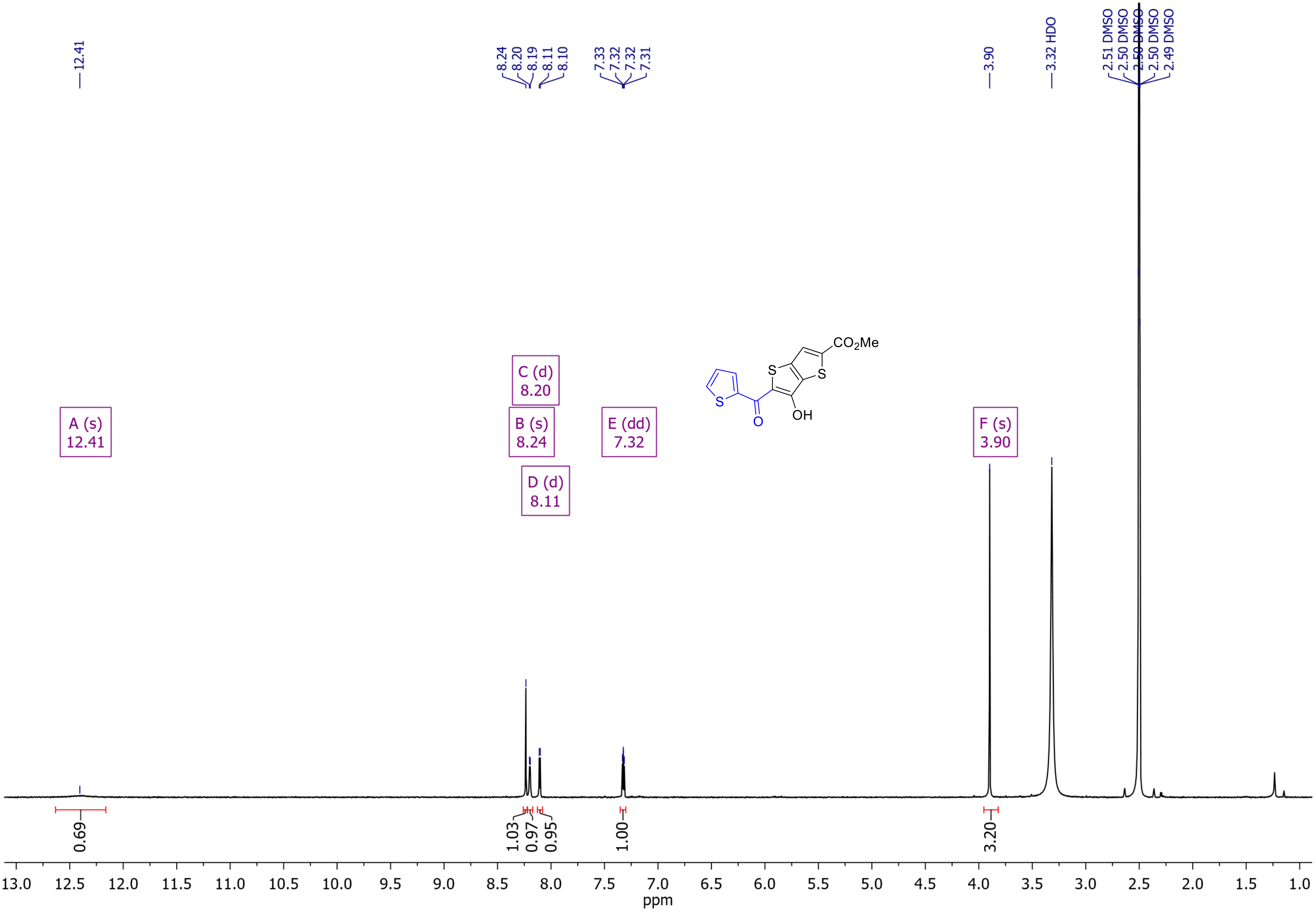

Methyl 6-hydroxy-5-(thiophene-2-carbonyl)thieno[3,2-*b*]thiophene-2-carboxylate (**9c**)

^{13}C NMR (126 MHz, DMSO-d_6)

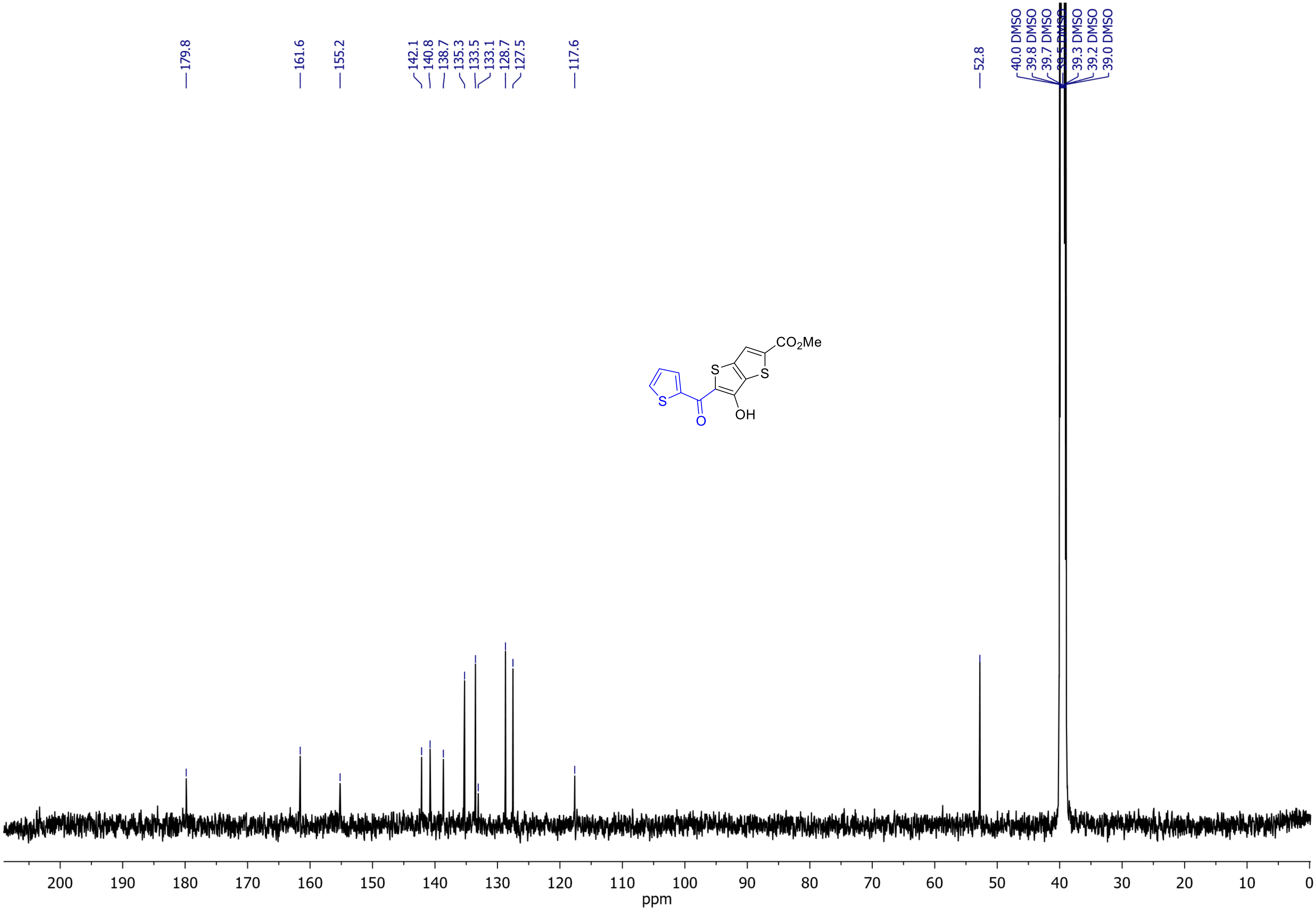

5. Copies of high-resolution mass spectra (HRMS)

Tetramethyl 3,3'-thiobis(thiophene-2,5-dicarboxylate) (**2**)

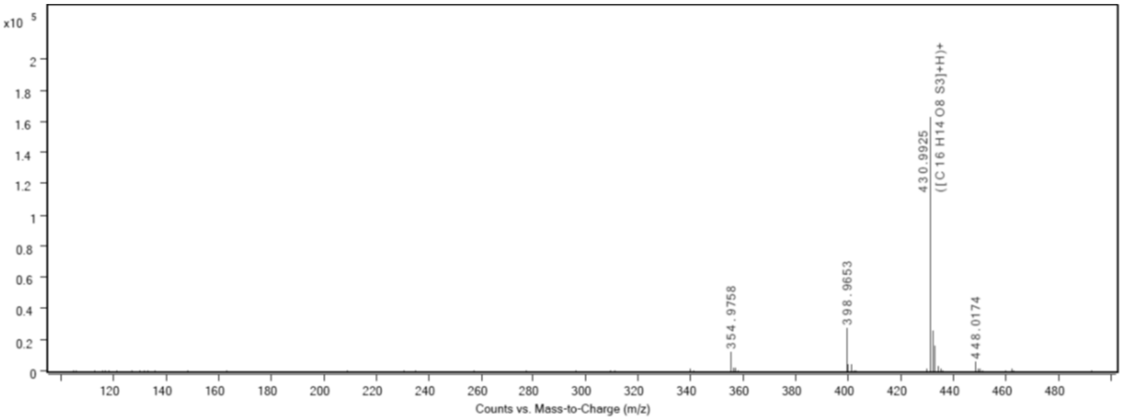

Tetramethyl 3,3'-disulfanediylbis(thiophene-2,5-dicarboxylate) (**3**)

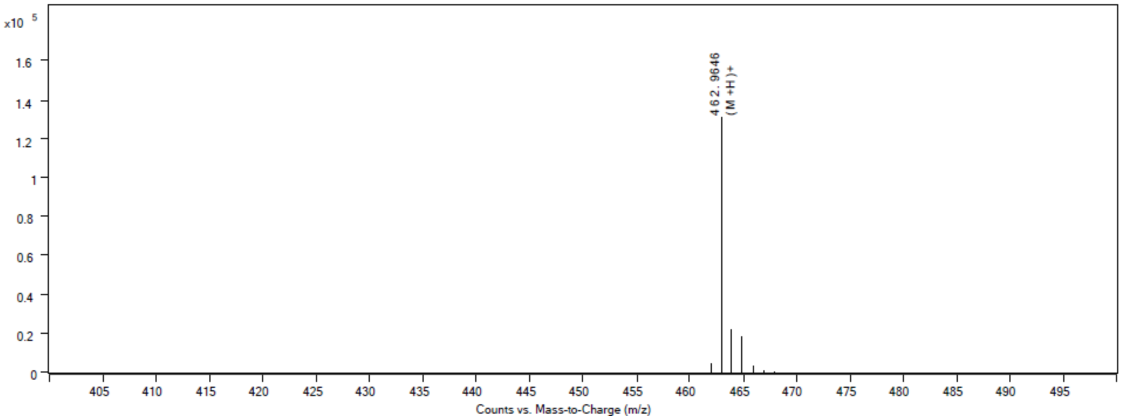

Dimethyl 3-[(4-cyanobenzyl)thio]thiophene-2,5-dicarboxylate (**4a**)

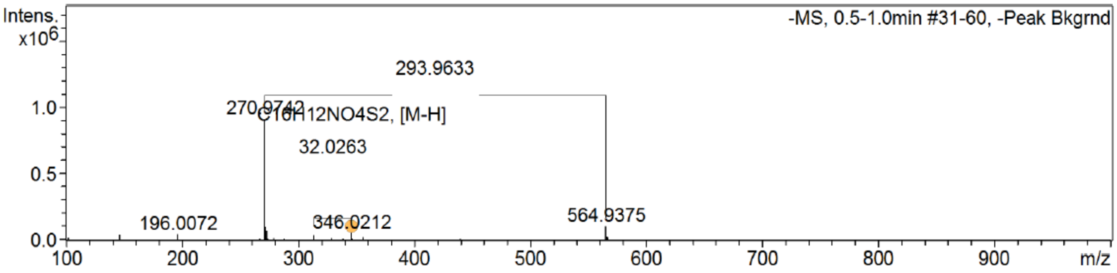

Dimethyl 3-{[4-(methoxycarbonyl)benzyl]thio}thiophene-2,5-dicarboxylate (**4b**)

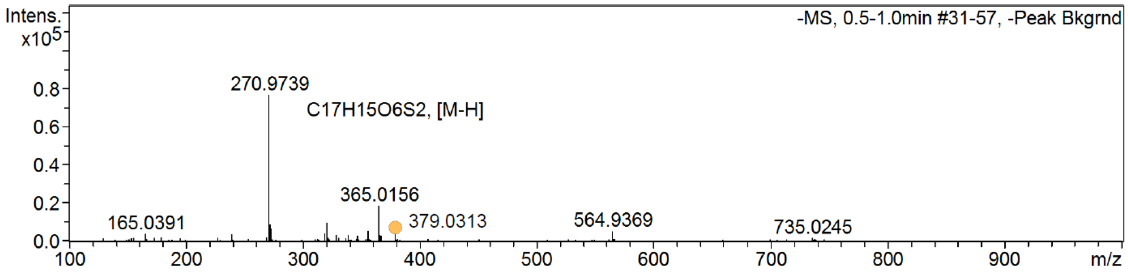

Dimethyl 3-(benzylthio)thiophene-2,5-dicarboxylate (**4c**)

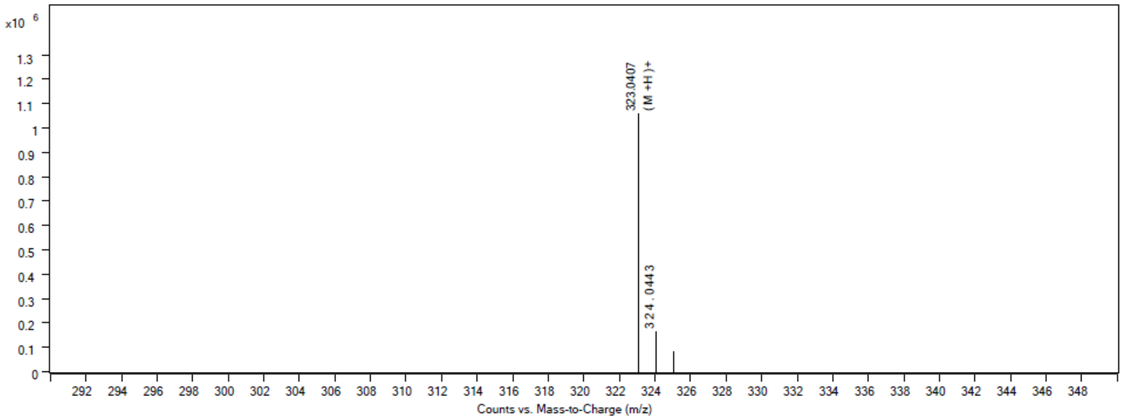

Dimethyl 3-[(4-fluorobenzyl)thio]thiophene-2,5-dicarboxylate (**4d**)

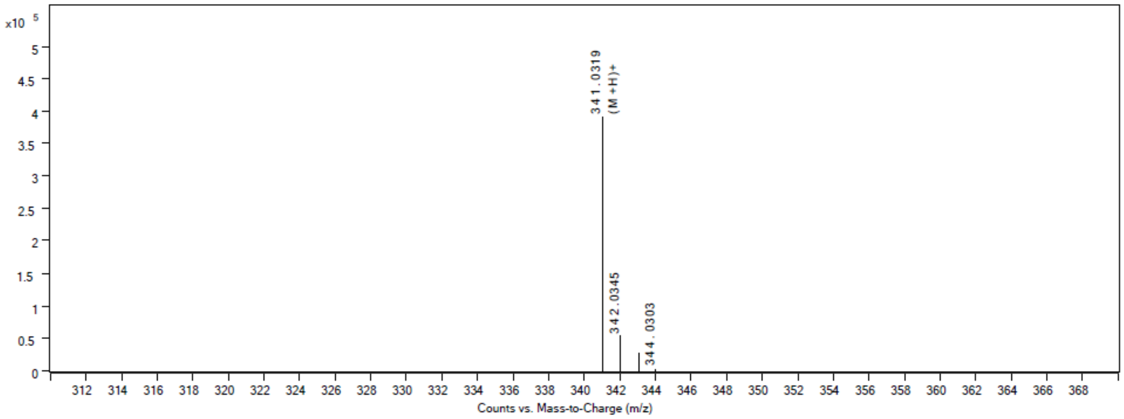

Dimethyl 3-[(3,4-difluorobenzyl)thio]thiophene-2,5-dicarboxylate (**4e**)

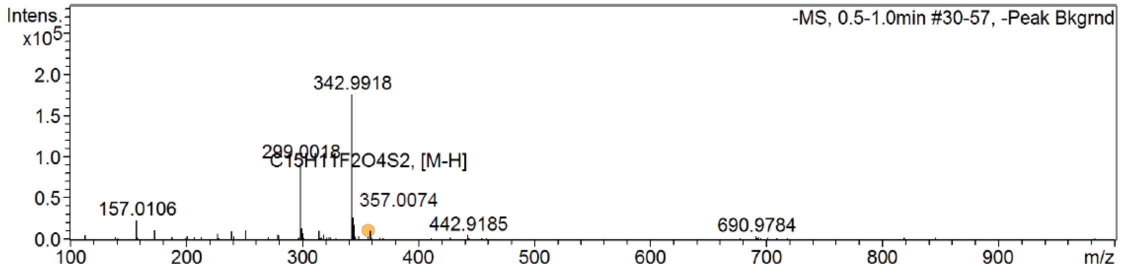

Dimethyl 3-{[4-(trifluoromethyl)benzyl]thio}thiophene-2,5-dicarboxylate (**4f**)

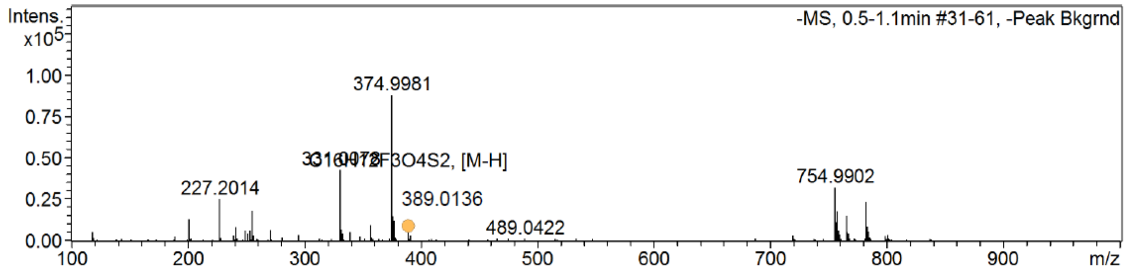

Dimethyl 3-[(benzo[*d*][1,3]dioxol-5-ylmethyl)thio]thiophene-2,5-dicarboxylate (**4g**)

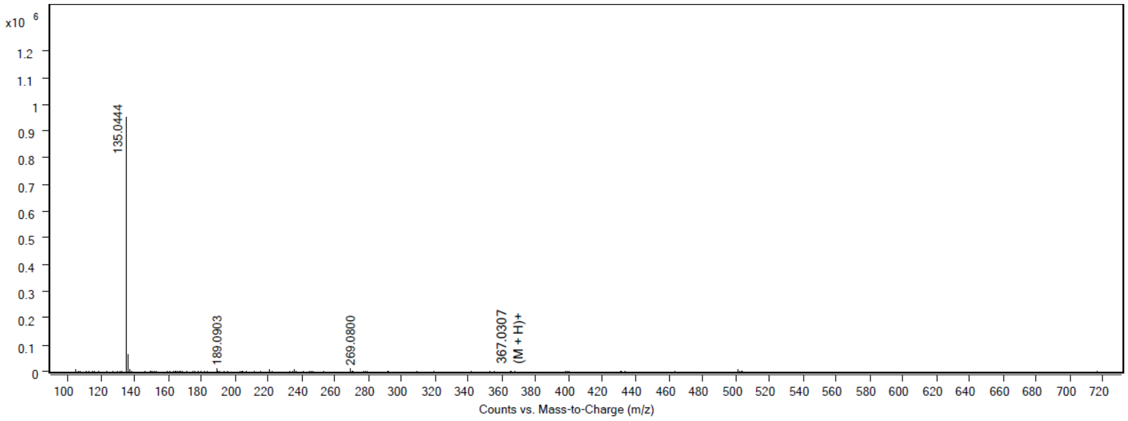

Dimethyl 3-[(thiophen-2-ylmethyl)thio]thiophene-2,5-dicarboxylate (**4h**)

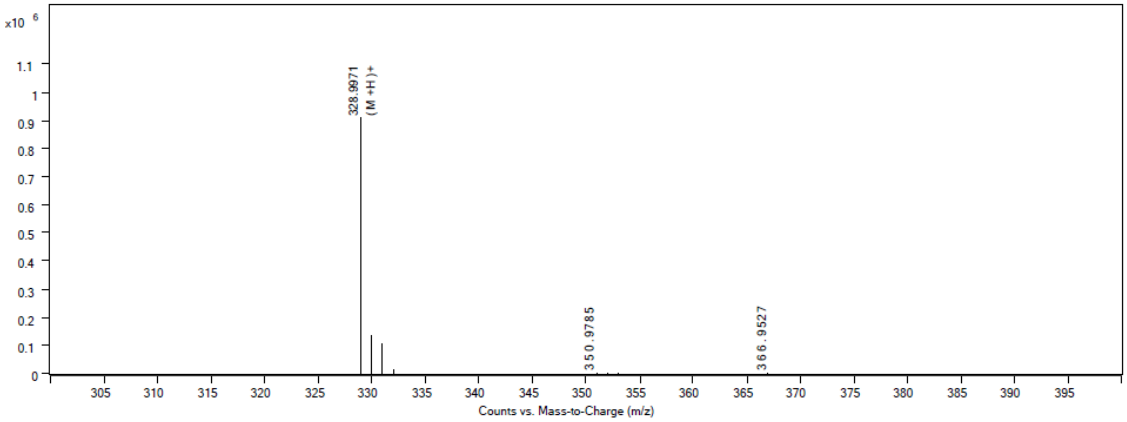

Dimethyl 3-[(cyanomethyl)thio]thiophene-2,5-dicarboxylate (**5a**)

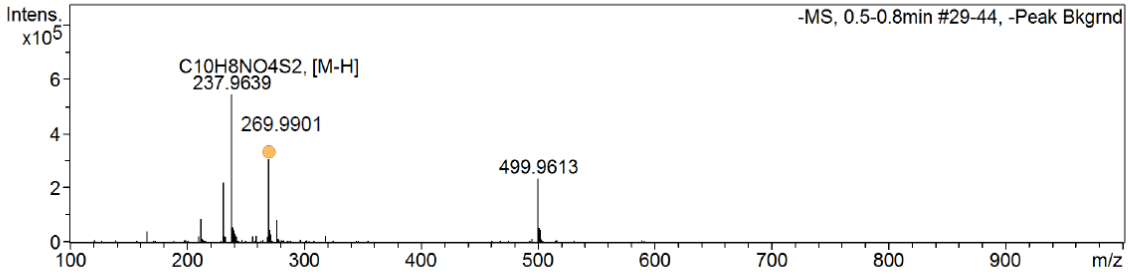

Dimethyl 3-[(2-oxo-2-phenylethyl)thio]thiophene-2,5-dicarboxylate (**6a**)

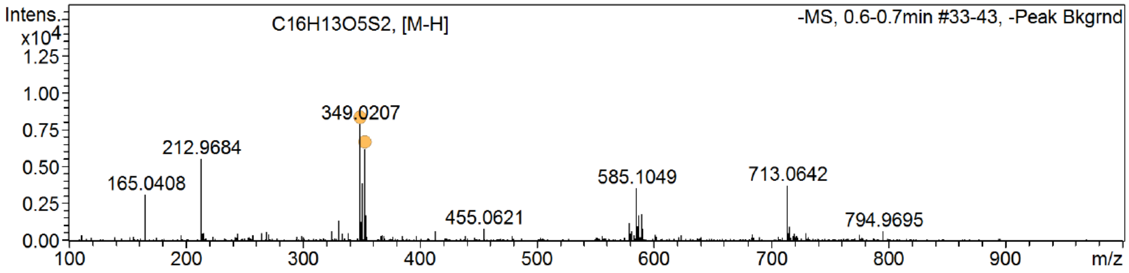

Dimethyl 3-{[2-(4-fluorophenyl)-2-oxoethyl]thio}thiophene-2,5-dicarboxylate (**6b**)

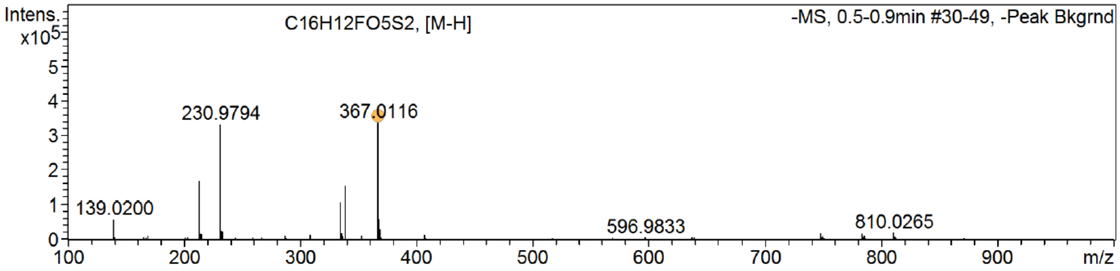

Dimethyl 3-{[2-oxo-2-(thiophen-2-yl)ethyl]thio}thiophene-2,5-dicarboxylate (**6c**)

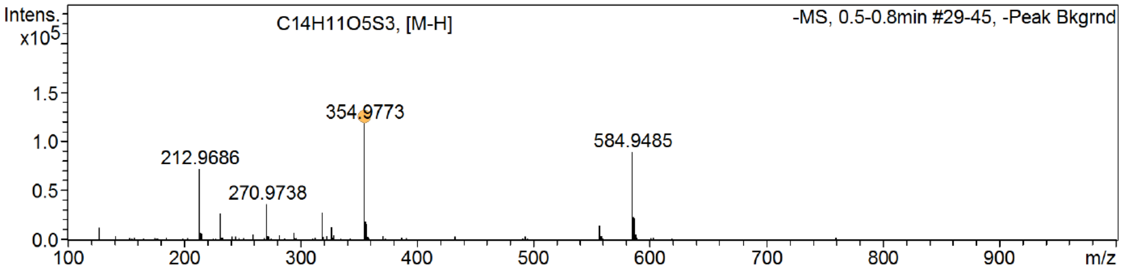

Methyl 5-(4-cyanophenyl)-6-hydroxythieno[3,2-*b*]thiophene-2-carboxylate (**7a**)

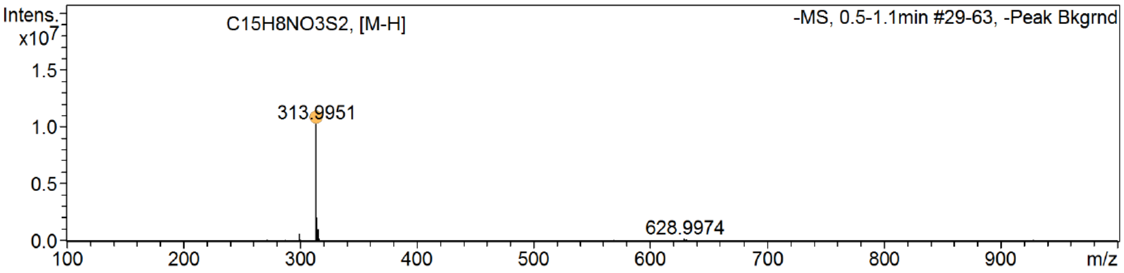

Methyl 6-hydroxy-5-[4-(methoxycarbonyl)phenyl]thieno[3,2-*b*]thiophene-2-carboxylate (**7b**)

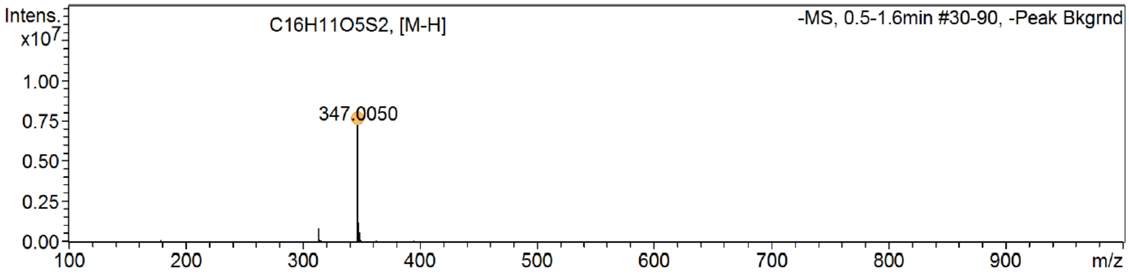

Methyl 5-cyano-6-hydroxythieno[3,2-*b*]thiophene-2-carboxylate (**8a**)

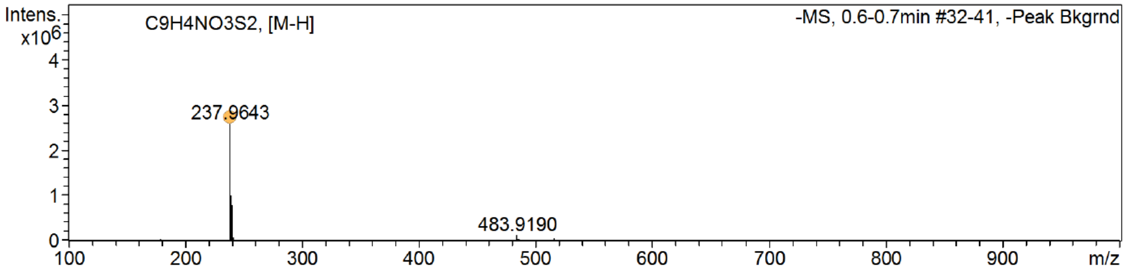

5-(4-Fluorobenzoyl)-6-hydroxythieno[3,2-*b*]thiophene-2-carboxylic acid (**9bA**)

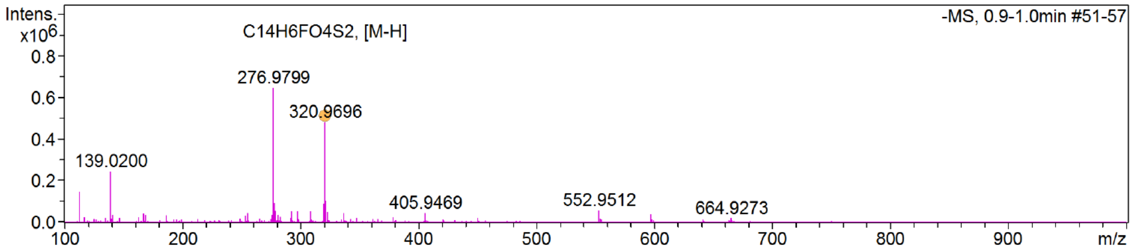

Methyl 5-benzoyl-6-hydroxythieno[3,2-*b*]thiophene-2-carboxylate (**9a**)

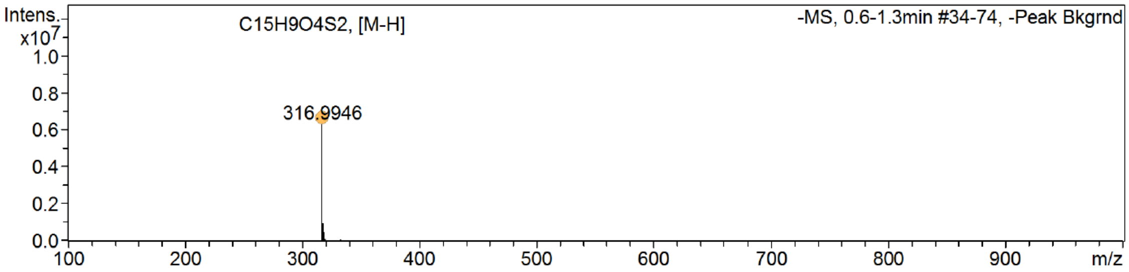

Methyl 5-(4-fluorobenzoyl)-6-hydroxythieno[3,2-*b*]thiophene-2-carboxylate (**9b**)

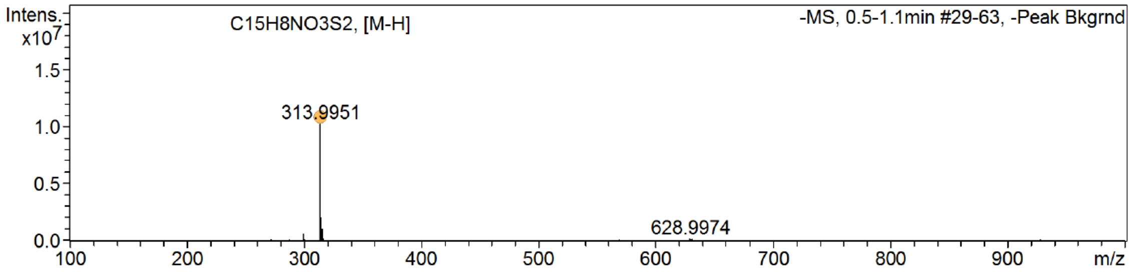

Methyl 6-hydroxy-5-(thiophene-2-carbonyl)thieno[3,2-*b*]thiophene-2-carboxylate (**9c**)

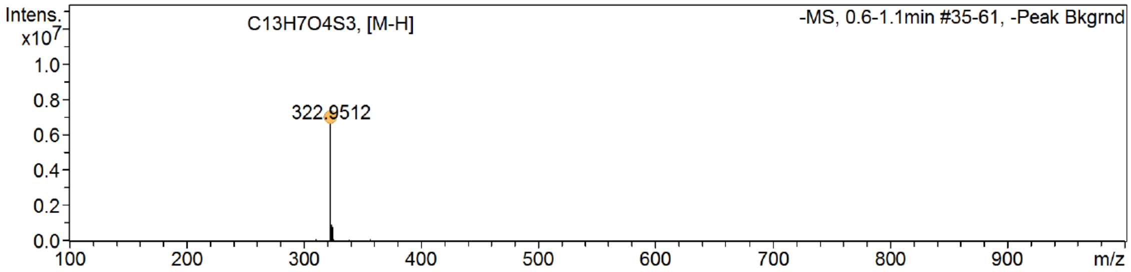

Supplement: File 1 — Full experimental details, characterization data, copies of NMR spectra and HRMS for all new compounds. [file Beilstein_J_Org_Chem-21-2489-s001.pdf]
